# A neural basis for learning sequential memory in brain loop structures

**DOI:** 10.3389/fncom.2024.1421458

**Published:** 2024-08-05

**Authors:** Duho Sihn, Sung-Phil Kim

**Affiliations:** Department of Biomedical Engineering, Ulsan National Institute of Science and Technology, Ulsan, Republic of Korea

**Keywords:** behavioral sequence, cell assembly, loop structure, self-generation, sequential memory

## Abstract

**Introduction:**

Behaviors often involve a sequence of events, and learning and reproducing it is essential for sequential memory. Brain loop structures refer to loop-shaped inter-regional connection structures in the brain such as cortico-basal ganglia-thalamic and cortico-cerebellar loops. They are thought to play a crucial role in supporting sequential memory, but it is unclear what properties of the loop structure are important and why.

**Methods:**

In this study, we investigated conditions necessary for the learning of sequential memory in brain loop structures via computational modeling. We assumed that sequential memory emerges due to delayed information transmission in loop structures and presented a basic neural activity model and validated our theoretical considerations with spiking neural network simulations.

**Results:**

Based on this model, we described the factors for the learning of sequential memory: first, the information transmission delay should decrease as the size of the loop structure increases; and second, the likelihood of the learning of sequential memory increases as the size of the loop structure increases and soon saturates. Combining these factors, we showed that moderate-sized brain loop structures are advantageous for the learning of sequential memory due to the physiological restrictions of information transmission delay.

**Discussion:**

Our results will help us better understand the relationship between sequential memory and brain loop structures.

## 1 Introduction

Behaviors, including movement or language, consist of a sequence of events. To learn and reproduce a sequence of events, cognitive systems often form sequential memory. It has been suggested that brain loop structures play an important role in subserving sequential memory (Gisiger and Boukadoum, 2018; Janacsek et al., 2020; Logiaco et al., 2021). In the present study, loop structures in the brain only refer to a network in which multiple, anatomically segregated, brain areas are connected in a closed-form: for example, brain area *X* is connected to area *Y*, *Y* is connected to *Z*, and *Z* is connected back to *X* (*X* → *Y* → *Z* → *X*).

Loop structures are widely distributed in the brain. Among them, brain loop structures related to movement or language include parallel cortico-basal ganglia-thalamic loops (Alexander et al., 1986; Lee et al., 2020; Foster et al., 2021) and cortico-cerebellar loops (Middleton and Strick, 2000; Kelly and Strick, 2003; Ramnani, 2006). Brain injury research has revealed that damage to these structures impairs movement or language functions (Alexander et al., 1986; Middleton and Strick, 2000; Vargha-Khadem et al., 2005; Ramnani, 2006; Aoki et al., 2019; Chang and Guenther, 2020; Lee et al., 2020). Furthermore, parallel loop structures are related to the parallel modulation of different behaviors. For example, a loop passing through the ventrolateral striatum is involved in licking, and a loop passing through the medial striatum is involved in turning (Lee et al., 2020).

It has been shown that loop structures in the brain support the formation and consolidation of sequential memory related to movement or language (for a review, see Rusu and Pennartz, 2020). This relationship between these structures and sequential memory has mainly been proven by experimental results: that is, the observation of increased neural activity in these structures associated with sequential memory, or disruption of sequential memory by damage to these structures. Recently, computational models for the formation of sequential memory have been proposed using recurrent networks (Maes et al., 2020; Cone and Shouval, 2021). These studies showed that spatiotemporal patterns of inputs can be learned on recurrent structures that are randomly connected to each other by a biologically plausible learning rule. However, from a computational model perspective, it still remains unclear how loop structures can form sequential memory since recurrent networks in the previous studies do not form a loop structure that consisting of multiple, anatomically segregated, brain areas. In fact, while closed loops can occur within a recurrently connected network, this study investigates them at a larger scale with multiple, individual areas. Although the formation of sequential memory can be computationally explained by recurrent networks, loop structures of this type are known to subserve sequential memory within the brain (for a review, see Rusu and Pennartz, 2020). Since recurrent networks may not fully elucidate how sequential memory is formed via these structures in the brain, it is more pertinent to directly investigate the role of loop structures in the creation of sequential memory.

One of the key properties of computational models built for loop structures is the size of the structures, i.e., the number of nodes in a loop. Some computational studies have demonstrated that the size of neuronal networks affects connectivity (Meisel and Gross, 2009) or energy-efficiency (Yu and Yu, 2017). If the number of neurons in a node is fixed, then increasing the size of the neuronal networks can be thought of as increasing the loop size. However, the relationship between the size of the loop structure and the formation of sequential memory is unknown. Another property to be considered for modeling these structures is time delay. Information transfer between nodes in these structures via neuronal signal transmission should accompany time delay in sequential neural activations of nodes. However, it is also unexplored how such time delay in these structures contributes to the formation of sequential memory.

In this study, we assume that the formation of sequential memory draws upon delayed information transmission across loop structures. To investigate the learning of sequential memory over these structures, we devise a basic neural activity model with delayed information transmission. Using this model, we analyze structural conditions under which sequential memory can emerge, particularly focusing on the size of loop structures. We confirm our theoretical prediction through a computer simulation.

## 2 Theory of the learning and retrieval of sequential memory

The theory of the formation of sequential memory will be presented in the following order: Section 2.1: we introduce the conceptual conditions necessary for the formation of sequential memory in brain loop structures; Section 2.2: we introduce a simplified neural model, referred to as the basic neural activity model, which is used to facilitate the mathematical analysis of the conceptual conditions above; Section 2.3: we analyze the mathematical necessary conditions for enabling the formation of sequential memory, building upon the basic neural activity model; and Section 2.4: we mathematically assess the likelihood of the learning of sequential memory.

We first mathematically define terms related to neural representations. Let *X*_*t*_ be a neural representation of the brain area *X* at time *t*. *X* is the set of all cell assemblies (as a representative of a specific subpopulations of excitatory and inhibitory neurons with strong synaptic connections) in the brain area and a neural representation *X*_*t*_ ⊆ *X* is the set of active cell assemblies at time *t*. An active cell assembly is defined as a cell assembly in which, for example, more than 50% of neurons are activated. We assumed that all cell assemblies have separated subpopulations of neurons within the same brain area, but they are interconnected between different brain areas. Nearby neurons are more likely to be connected by synapses (Schnepel et al., 2015; for a review, Boucsein et al., 2011), and cell assemblies are subpopulations of neurons connected by these strong synapses (Sadeh and Clopath, 2021). Therefore, the assumption that cell assemblies within the same brain area are separated implies weak connectivity between neurons in different cell assemblies. The recurrent connections that are pervasive in the brain indicate strong connectivity between neurons within a single cell assembly in our model. As such, we did not define the recurrent connections explicitly in our model as a cell assembly containing the recurrent connections is a basic element. Furthermore, we say that *X*_*t*_ is inhibited at time *t*+1, if and only if *X*_*t*_ ∩ *X*_*t*+1_ is an empty set. Unless otherwise specified, it is assumed that *X*_*t*_ ≠ *X*_*t*+ 1_.

Let *E*_*t*_ represent an event at time *t*, signifying its occurrence alongside its neural representations of *X*_*t*_, *Y*_*t*_, and *Z*_*t*_. These representations belong to brain areas related to *X*, *Y*, and *Z*, forming a loop structure in a sequence, *X*→*Y*→*Z*→*X*. While there may exist time delays between *X*_*t*_ and *Y*_*t*_, as well as between *Y*_*t*_ and *Z*_*t*_, it is important to note that all of *X*_*t*_, *Y*_*t*_, and *Z*_*t*_ constitute delayed neural representations of the same event *E*_*t*_. In other words, *X*_*t*_ is not the sole representation of *E*_*t*_, nor are *Y*_*t*_ and *Z*_*t*_ solely for delayed versions of *X*_*t*_.

### 2.1 Delayed information processing in brain loop structures

**Figure 1:**
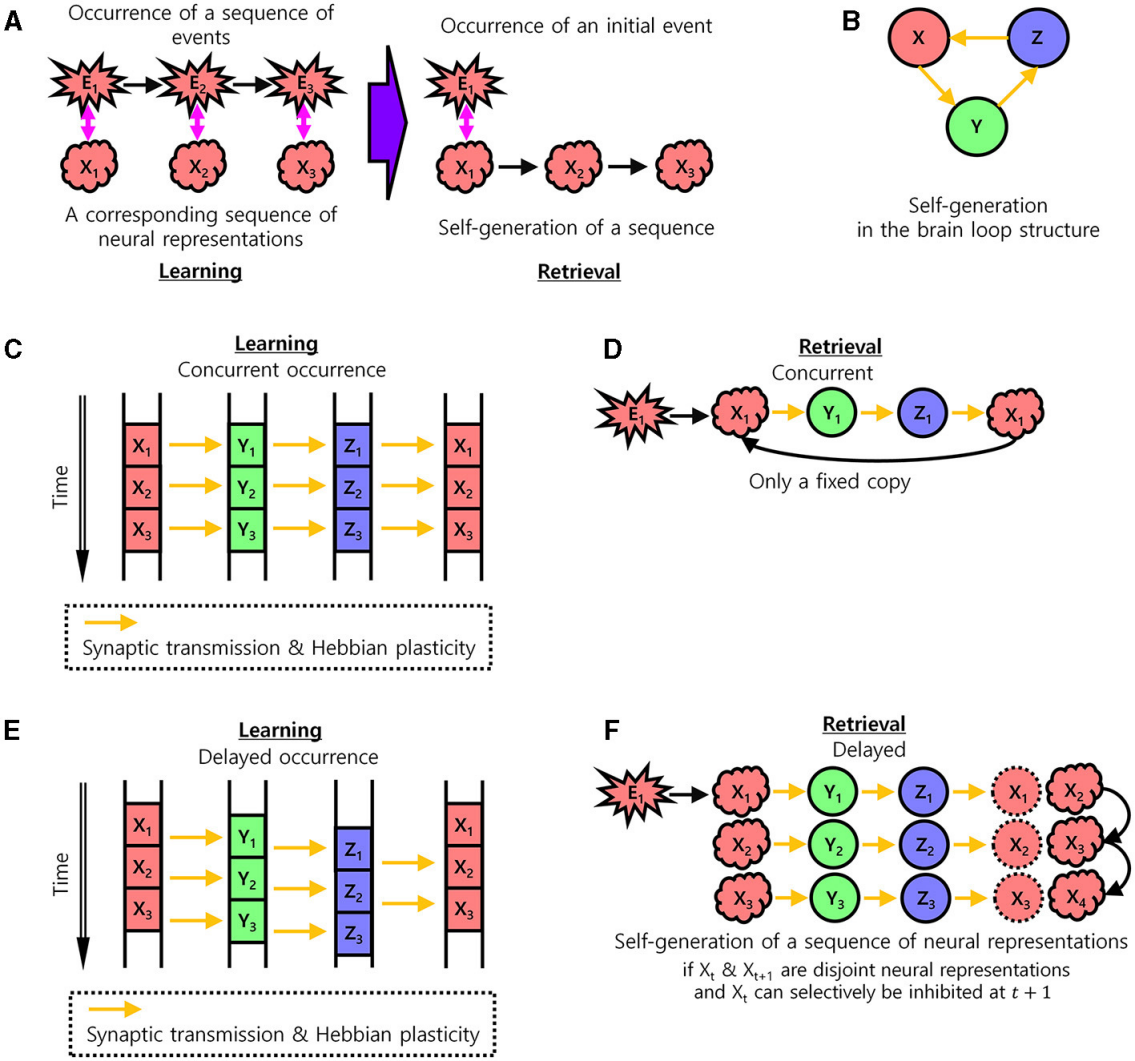
The learning of sequential memory in brain loop structures. **(A)** Self-generation of a sequence of neural representations of behavioral events. In the learning step, a corresponding neural representation occurs for each behavioral event and is learned. In the retrieval step, a sequence of neural representations is self-generated by an initial behavioral event. *X*_*t*_: Neural representation of the brain area *X* at time *t*. Equivalently, *X*: Set of all cell assemblies in the brain area, *X*_*t*_ ⊆ *X*: Set of active cell assemblies in the brain area *X*. *E*_*t*_: Event at time *t*. Therefore, *E*_*t*_ is a simultaneous event with *X*_*t*_, *Y*_*t*_, and *Z*_*t*_, even if *Y*_*t*_, and *Z*_*t*_ are delayed representations. **(B)** The brain loop structure for the self-generation of a sequence of neural representations. **(C, D)** Learning with concurrent occurrence. **(C)** Information transmission and Hebbian plasticity in case of the concurrent occurrence of neural representations during the learning of sequential memory. Arrows indicate pathways through which synaptic transmission takes place and results in Hebbian plasticity. **(D)** No self-generation of the sequence of neural representations in case of the concurrent occurrence of neural representations during the retrieval of sequential memory. **(E, F)** Learning with delays occurrence. Same as **(C, D)** with non-zero delays. **(E)** Information transmission and Hebbian plasticity in case of the delayed occurrence of neural representations during the learning of sequential memory. The learning of sequential memory. **(F)** Self-generation of the sequence of neural representations in case of the delayed occurrence of neural representations (if there is inhibition of the previous neural representation) during the retrieval of sequential memory.

Behaviors related to movement or language consist of a sequence of events. For subsequent successful behavior, a sequence of events must be reproducible. Such a sequence of events may induce the learning of a sequence of corresponding neural representations in the brain. These neural representations can be seen as neural substrates of sequential behaviors, which construct an internal model of the corresponding behavior (McNamee and Wolpert, 2019; Yildizoglu et al., 2020; Mok and Love, 2022). In the present study, the sequential memory problem was stated as how the sequence of neural representations of a sequence of behavioral events can be self-generated in the activation of all the cell assemblies in each consecutive brain area (Figure 1A). Since brain loop structures play important roles in forming sequential memory (Gisiger and Boukadoum, 2018; Logiaco et al., 2021), we assumed that this structure enables the self-generation of a sequence of neural representations (Figure 1B). Self-generation here means that when a part of a sequence of neural representations is stimulated, the rest of the sequence is automatically activated.

Let *X*, *Y*, and *Z* be brain areas forming a loop structure such that *X*→*Y*→*Z*→*X* (Figure 1B). Let *X*_*t*_, *Y*_*t*_, and *Z*_*t*_ be neural representations of a certain behavioral event, *E*_*t*_, occurring at time *t* in each area. If this behavioral event is concurrently represented in this structure with no time delay, i.e., if *X*_*t*_, *Y*_*t*_, and *Z*_*t*_ concurrently occur, then the Hebbian plasticity would associate neural representations, *X*_*t*_ → *Y*_*t*_, *Y*_*t*_ → *Z*_*t*_, and *Z*_*t*_ → *X*_*t*_ (Figure 1C). The Hebbian plasticity can be formulated as:


(1) $$\begin{array}{l} \tau \begin{array}{c} \frac{dw_{ji}}{dt}=\hat{x_{i}}\left(t\right)\hat{y_{j}}\left(t\right) \end{array} \end{array}$$

where *w*_*ji*_ is a synaptic weight from a cell assembly $\hat{x_{i}}$ to another cell assembly $\hat{y_{j}}$ and τ is a time constant. A cell assembly $\hat{x_{i}}$ has the value 1 or 0, when active or non-active, respectively. In this case, when a behavioral event induces the neural representation *X*_*t*_ in the area *X* at time *t*, it would induce *Y*_*t*_ and *Z*_*t*_ concurrently, generating *X*_*t*_ again by *Z*_*t*_ → *X*_*t*+1_*X*_*t*_. Therefore, this concurrent loop would result in the learning of the self-generation of only a fixed neural representation *X*_*t*_ → *X*_*t*_ by the logical transitive relation, without learning to self-generate the next neural representation *X*_*t*+1_, (Figure 1D). As such, the neural representation *X*_*t*+1_ would only be induced by an external associated behavioral event, *E*_*t*+1_, not by the internal loop structure, making it difficult to self-generate sequential memory.

In contrast, if the neural representations of behavioral events are delayed in the brain loop structure, i.e., if *X*_*t*_, *Y*_*t*_, and *Z*_*t*_ are sequentially induced with non-zero delays between them when an event *E*_*t*_ occurs, then the Hebbian plasticity may associate both neural representations, *Z*_*t*_ → *X*_*t*_ and *Z*_*t*_ → *X*_*t*+1_, from *Z* to *X* (Figure 1E). If *X*_*t*_ and *X*_*t*+1_ are disjoint and the previous neural representation *X*_*t*_ can be selectively inhibited at time *t*+1, a sequence of neural representations *X*_*t*_ → *Y*_*t*_ → *Z*_*t*_ → *X*_*t*+1_ can be self-generated. Afterward, the generation of *X*_*t*+1_ would induce *Y*_*t*+1_ and so on. Therefore, this delayed information processing along with the inhibition of previous neural representations of *X*_*t*_ would result in the self-generation of a sequence of disjoint neural representations, *X*_*t*_ → *X*_*t*+1_ → … → *X*_*T*_ in the area *X* (Figure 1F). This serves as a basis for generating the sequential memory of behavioral events, *E*_*t*_ → *E*_*t*+1_ → … → *E*_*T*_, via the internal loop structure without the occurrence of external behavioral events. As such, finding a biologically plausible model that supports this self-generation of a sequence of neural representations in this structure would be important. Below, we present a biologically plausible model for the delayed information processing and the inhibition of the previous neural representation.

To emphasize, learning based on Hebbian plasticity implemented in our model primarily encodes the sequence of neural representations of events rather than precise timing. Consequently, the delay at which each neural representation sequentially appears may vary depending on the specific context. The proposed model learns sequences of neural representations from the examples of neural representations of events. Note that the stages of neural dynamics (*t*, *t*+1) in Figures 1C, E evolve on the order of tens of milliseconds. The time difference of (*t*, *t*+1) (Figures 1C, E) in the neural representation of each event does not indicate a behavioral transition. In fact, a single behavioral transition *X*_*T*_, *X*_2*T*_, and *X*_3*T*_ can occur over multiple stages of neural dynamics *t*, *t*+1, *t*+2, …, *t*+*T*, …, *t*+2*T*, …, and *t*+ 3*T*.

### 2.2 Basic neural activity model

**Figure 2:**
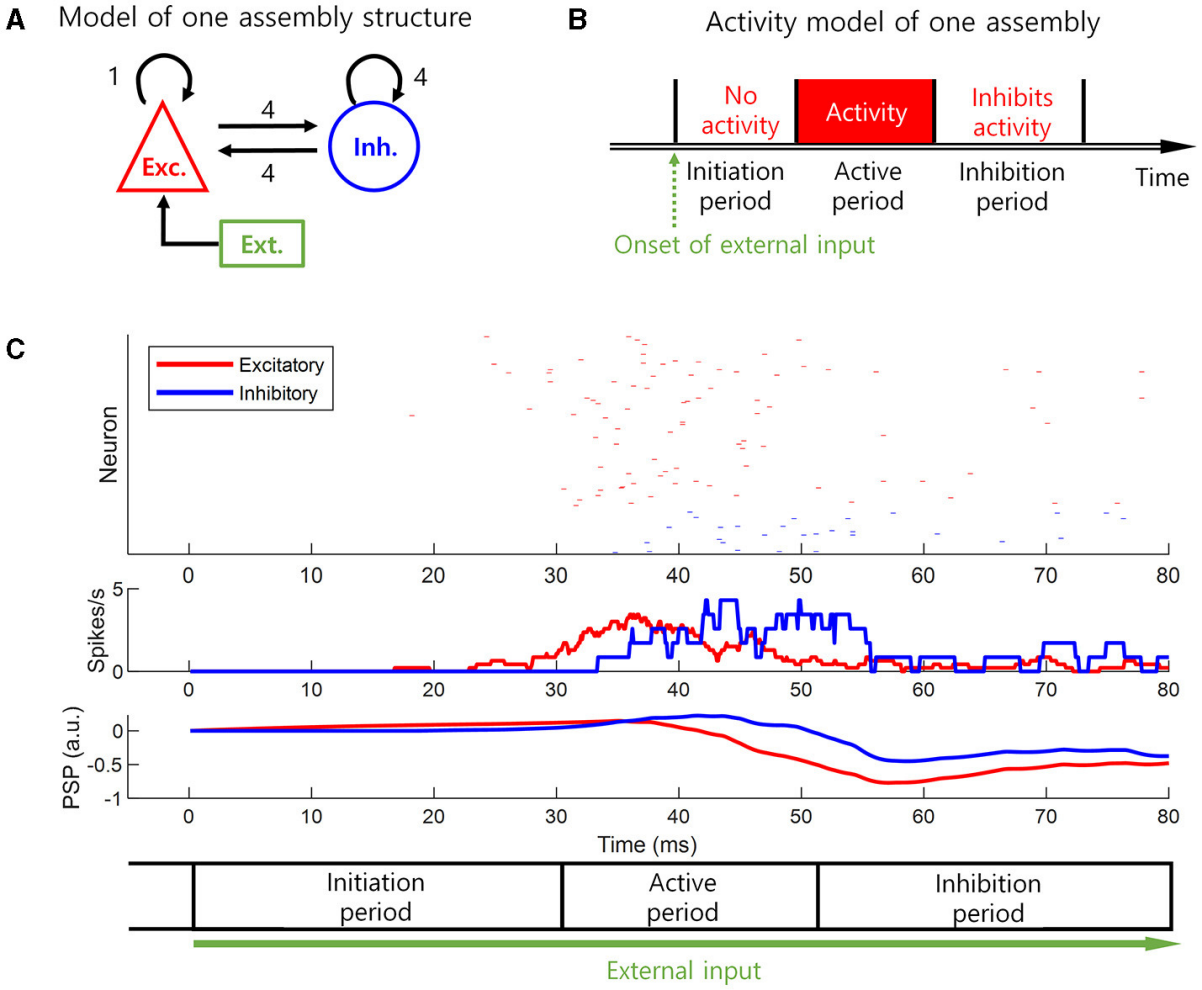
Basic neural activity model. A biologically plausible model of delayed information processing and previous neural representation inhibition. This is a neural activity model of a cell assembly, i.e., subpopulation, in the loop structure. The model is based on the excitatory-inhibitory balanced networks and the flow of postsynaptic potential (PSP) changes. **(A)** The excitatory-inhibitory balanced networks that form the basic neural activity model. **(B)** The basic neural activity model. This model has a value of 1 during the active period, and a value of 0 during the initiation or inhibition periods. **(C)** The simulation result to derive the basic neural activity model based on the setting in **(A)**. The spike rate is smoothed by a 3-ms uniform moving window, where the length of this moving window corresponds to the refractory period. The initiation and inhibition periods are set based on the slow change of PSP. PSP is slowly decayed. The initiation period corresponds to delayed information processing, and the inhibition period corresponds to previous neural representation inhibition.

To present a biologically plausible model of the delayed information processing and the inhibition of previous neural representation, we modeled the neural activity of a cell assembly (i.e., a subpopulation of co-active neurons). This basic neural activity model was based on excitatory-inhibitory balanced networks (Figure 2A) and the flow of postsynaptic potential (PSP) changes (Figure 2C). The flow of PSP changes in response to an external input can be described in three periods (Figures 2B, C). When excitatory external input is received by excitatory neurons, the PSP of the excitatory neurons starts to increase in the beginning until it exceeds a threshold level. We call this first period initiation period with no neural activity of excitatory neurons as the PSP remains subthreshold level. When the PSP of the excitatory neurons crosses the threshold, it increases the PSP of both the excitatory and inhibitory neurons via synaptic interactions. We call this second period active period with apparent neural activity of excitatory neurons. When the PSP of the inhibitory neurons exceeds the threshold, the inhibitory neurons begin to inhibit the excitatory neurons. When the input from the inhibitory neurons is greater than that from the excitatory neurons, the activity of the excitatory neuron is inhibited even though the external input constantly lasts. This period persists for a certain amount of time, even in the absence of excitatory input from the excitatory neuron because of the duration of the PSP of the inhibitory neuron. We call this third period inhibition period with no neural activity of the excitatory neurons (Figures 2B, C). Therefore, our basic neural activity model has three consecutive periods: (1) initiation, (2) active, and (3) inhibition periods. Apparent neural activity is present in the active period but absent in the initiation and inhibition periods (Figure 2B). The length of the initiation period and inhibition period depends on the rise and decay times of the PSP (Figure 2C).

In this subsection, a basic neural activity model, a model of the cell assembly unit, was derived from the population of spiking neurons. From now on, we will perform mathematical analysis related to the formation of sequential memory using the basic neural activity model derived here, rather than the model of spiking neurons.

### 2.3 Necessary conditions for the learning and retrieval of sequential memory

Each brain area in a loop structure consists of multiple cell assemblies, where a cell assembly is defined as the substrate of a neural activity model introduced in the Section 2.2. Suppose that each cell assembly undergoes one cycle of the neural activity model, i.e., initiation-active-inhibition, with a random starting point, i.e., random phase. Let *t*_*N*_, *t*_*A*_, and *t*_*I*_ denote the length of the No-activity (initiation), Activity, and Inhibition periods, respectively. Note that *t*_*N*_ represents a delay from a cell assembly in one area to another cell assembly in the subsequent area. Let *N* be the number of areas in the loop structure.

We assume that *t*_*N*_, *t*_*A*_, and *t*_*I*_ are fixed over all the cell assemblies in each area over the loop structure. This does not have a significant impact on the conclusions of this study, especially when areal heterogeneity is not large. Let us assume that individual neural activity models defined on each cell assembly in an area share the same active period length *t*_*A*_, but start differently at a random time point, i.e., random phase. At an arbitrary time point *t*, each neural activity model undergoes in one of the active, initiation or inhibition periods. Then, right before *t*+*t*_*A*_, some models that start their active period at *t* will still be under the active period. Right after *t*+*t*_*A*_, those models that start their active period at *t* will end the active period and transit to the initiation, inhibition or new active period. If some of those models are under new active periods right after *t*+*t*_*A*_, these models change to new active periods exactly once as the active period spans *t*_*A*_. Therefore, between *t* and *t*+*t*_*A*_, the active period of every model will change to the initiation, inhibition or new active period exactly once. Since neural activity during the active period represents an event, we can assume that a combination of the activities of all cell assemblies representing an event changes into a different combination exactly once during a period of the length *t*_*A*_.

When the sequential activities of cell assemblies return to the first area *X* through the loop, i.e., delay *t*_*N*_ with *N* − 1 times, it must encounter the neural representation of a new event to form a sequential memory by Hebbian plasticity between co-active cell assemblies in the adjacent areas, where the sequential memory should consist of different neural representations, i.e., *X*_*t*_ → *X*_*t*+1_. Hence, it is necessary to have the following condition: the time during which information for sequential events returns through the loop must be >zero, i.e., (*N* − 1)*t*_*N*_ > 0. This condition yields *t*_*N*_ > 0.

When information for sequential events returns through the loop, it must encounter the *next* neural representation, i.e., an intermediate neural representation must not be omitted, in other words, *X*_*t*_ → *X*_*t*+1_ , but not $X_{t}\to X_{t+t^{\prime }}$ where *t*′>1. If neural activity in the loop returns to the first area *X* after the activation period ends, neural activity in the last area of the loop will activate the (t+j)-th neural activity in *X* where j > 1, causing the omission of the (t+1)-th neural activity in *X*. Consequently, the sequence of events would not be accurately represented. Hence it is necessary to have the condition that *Nt*_*N*_ ≤ *t*_*N*_ + *t*_*A*_, where *t*_*A*_ corresponds to the active period of a neural representation. This condition yields:


(2) $$\begin{array}{l} \begin{array}{c} t_{N}\leq \frac{t_{A}}{N-1}. \end{array} \end{array}$$

To inhibit previous neural representation (Figure 1F), the same cell assembly must not be active in succession. Let $\hat{x}$ be a cell assembly in the first area in the loop structure. A neural representation emerges in the first area during a period from *t*_*N*_ to *t*_*N*_ + *t*_*A*_ will be represented in the last area during a period from *Nt*_*N*_ to *Nt*_*N*_ + *t*_*A*_. In this case, the combination of the outputs of the neural activity models in the last area will be associated with $\hat{x}$ in the first area through Hebbian plasticity. That is, the combination of the outputs of the neural activity models in the last area can cause the activation of $\hat{x}$. If *Nt*_*N*_ + *t*_*A*_ is larger than *t*_*N*_ + *t*_*A*_ + *t*_*I*_, $\hat{x}$ may become active in succession. This leads to the loss of opportunity for the next event to be appropriately represented. To prevent this, it is necessary that *Nt*_*N*_ + *t*_*A*_ ≤ *t*_*N*_ + *t*_*A*_ + *t*_*I*_. This condition yields:


(3) $$\begin{array}{l} \begin{array}{c} t_{N}\leq \frac{t_{I}}{N-1}. \end{array} \end{array}$$

The inhibition period's role is to prevent cell assemblies activated in the neural representation of a previous event from consecutively remaining active, ensuring the generation of a distinct neural representation for the next event. However, as per Equation 2, (*N* − 1)*t*_*N*_ ≤ *t*_*A*_ is established, signifying that the activity in the last area is associated with the cell assembly $\hat{x}$ in the first area through Hebbian plasticity. Nevertheless, if Equation 3 is not satisfied—that is, if the activity in the last area persists even after the inhibition period of the first area has concluded—it can result in the continuous activation of cell assembly $\hat{x}$ due to the association facilitated by the aforementioned Hebbian plasticity.

**Figure 3:**
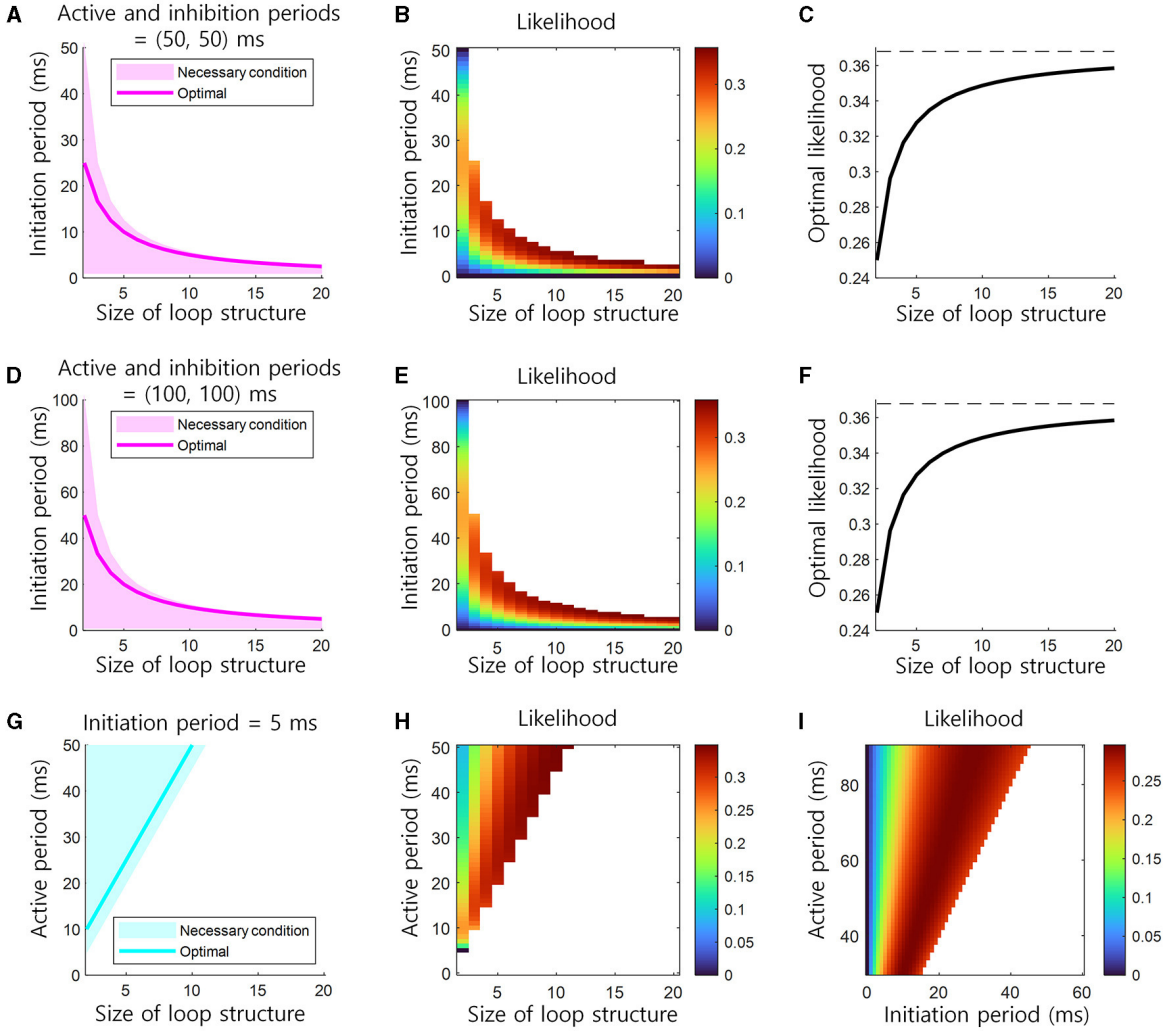
Conditions for the learning and retrieval of sequential memory in brain loop structures. **(A)** Necessary conditions for the learning and retrieval of sequential memory (see Equations 2, 3). The necessary condition of the initiation period as the function of the size of loop structure when the active and inhibition periods are 50 ms. The optimal, i.e., bold magenta line, corresponds to the location of ridge in **(B)**. **(B)** The likelihood of the learning of sequential memory, as the function of the initiation period and the size of loop structure when the active period is 50 ms (see Equation 4). **(C)** The optimal likelihood of the learning of sequential memory, as the function of the size of loop structure (see Equations 11, 12). This corresponds to the height of ridge in **(B)**. Top dashed black line indicates the upper bound of the optimal likelihood, *e*^−1^ ≈ 0.3679. **(D–F)** Similar to **(A–C)**, but the active and inhibition periods are 100 ms. **(G)** The necessary condition of the active period as the function of the size of loop structure when the initiation period are 5 ms. **(H)** The likelihood of the learning of sequential memory, as the function of the active period and the size of loop structure when the initiation period is 5 ms. **(I)** The likelihood as the function of both initiation and active periods, when the inhibition period is 50 ms and the size of loop structure is 3.

The conditions in Equation 2 are necessary for learning via Hebbian plasticity. The condition in Equation 3 is necessary for the inhibition of previous neural representation. If the lengths of the active (*t*_*A*_) or inhibition (*t*_*I*_) periods of a cell assembly are fixed, the possible range of information transmission delay (*t*_*N*_) is bounded by the inverse of the size of the loop structure (1/(*N* − 1)). In other words, as the size of this structure increases, the information transmission delay should decrease (Figures 3A, D). If the information transmission delay (*t*_*N*_) is fixed, the length of the active (*t*_*A*_) period should increase as the size of this structure (*N*) increases (Figure 3G).

We assume that *t*_*N*_, *t*_*A*_, and *t*_*I*_ are fixed over all the cell assemblies in each area over the loop structure. We acknowledge that this is a strong simplification because the brain would be highly heterogeneous in general. Nonetheless, we adopt this assumption to obtain simplified mathematical solutions so as to arrive at simplified conclusions. If we assume that *t*_*N*_, *t*_*A*_, and *t*_*I*_ are heterogeneous for *N* different brain areas, the necessary conditions are from the summation of different (*N* – 1)-terms instead of the (*N* – 1)-summation of the same terms. This would alter our equations as follows: $\overset{N}{\underset{i=2}{\sum }}t_{N,i}>0$, instead of (*N* − 1)*t*_*N*_>0; Equation 2 would become $\overset{N}{\underset{i=1}{\sum }}t_{N,i}\leq t_{N,1}+t_{A,1\;}$, instead of *Nt*_*N*_ ≤ *t*_*N*_ + *t*_*A*_.; and Equation 3 would become $\overset{N}{\underset{i=1}{\sum }}t_{N,i}+t_{A,N\;}\leq t_{N,1}+t_{A,1\;}+t_{I,1}$, instead of *Nt*_*N*_ + *t*_*A*_ ≤ *t*_*N*_ + *t*_*A*_ + *t*_*I*_. However, using the summation of different (*N* – 1)-terms instead of the (*N* – 1)-summation of the same terms does not change the direction of the conclusions of this study because each term has same characteristics.

### 2.4 The likelihood of the learning of sequential memory

The next step is to determine the information transmission delay for which sequential memory can learn with the most likelihood. Here, the likelihood indicates a probability that sequential memory can be learned in a loop structure. For every pair of adjacent areas, we calculated the “probability” of sequential memory learning between them and multiplied all those “probabilities” to form the likelihood. Hence, the likelihood here takes a value between 0 and *e*^−1^≈0.3679, with higher values indicating higher possibilities of sequential memory learning. The value of *e*^−1^≈0.3679 is derived below in this subsection. In the present study, the learning of sequential memory is based on Hebbian plasticity which associates co-active cell assemblies. The likelihood of the learning of sequential memory between the adjacent areas, i.e., *X*_*t*_ → *Y*_*t*_ or *Y*_*t*_ → *Z*_*t*_, except the last to first areas, i.e., *Z*_*t*_ → *X*_*t*+1_, can be described as the proportion of a duration that two areas represent the same event in one cycle of the neural representation of an event. Since *t*_*A*_ is the duration of the representation of an event and *t*_*N*_ is the time limiting the representation of the same event, the likelihood can be written as the ratio: (*t*_*A*_−*t*_*N*_)/*t*_*A*_ = 1−*t*_*N*_/*t*_*A*_. The likelihood from the last to first areas, i.e., *Z*_*t*_ → *X*_*t*+1_, is the proportion of the representation time of two different events that the last area represents a current event and the first area represents the next event. This proportion of time satisfies the condition that the duration of the neural representation of a current event in the last area overlaps the duration of the neural representation of the next event in the first area, which can be written as the ratio of the duration (*N* − 1)*t*_*N*_ to the duration *t*_*A*_ of the next neural representation: (*N* − 1)*t*_*N*_/*t*_*A*_. Therefore, the likelihood in the loop structure is the product of all proportions (Figure 3B):


(4) $$\begin{array}{l} f\left(t_{N},\;t_{A}\right)={\left(1-\frac{t_{N}}{t_{A}}\right)}^{N-1}\frac{\left(N-1\right)t_{N}}{t_{A}}. \end{array}$$

To maximize the likelihood function, we take its partial derivative:


(5) $$\begin{array}{l} \begin{array}{c} \frac{\partial }{\partial t_{N}}f\left(t_{N},\;t_{A}\right)=\frac{\partial }{\partial t_{N}}\left({\left(1-\frac{t_{N}}{t_{A}}\right)}^{N-1}\right)\frac{\left(N-1\right)t_{N}}{t_{A}}\\ +{\left(1-\frac{t_{N}}{t_{A}}\right)}^{N-1}\frac{\partial }{\partial t_{N}}\left(\frac{\left(N-1\right)t_{N}}{t_{A}}\right). \end{array} \end{array}$$

The first term becomes:


(6) $$\begin{array}{l} \begin{array}{c} \frac{\partial }{\partial t_{N}}\left({\left(1-\frac{t_{N}}{t_{A}}\right)}^{N-1}\right)\frac{\left(N-1\right)t_{N}}{t_{A}}\\ =\left(N-1\right){\left(1-\frac{t_{N}}{t_{A}}\right)}^{N-2}\left(-\frac{1}{t_{A}}\right)\frac{\left(N-1\right)t_{N}}{t_{A}}. \end{array} \end{array}$$

The second term becomes:


(7) $$\begin{array}{l} {\left(1-\frac{t_{N}}{t_{A}}\right)}^{N-1}\frac{\partial }{\partial t_{N}}\left(\frac{\left(N-1\right)t_{N}}{t_{A}}\right)={\left(1-\frac{t_{N}}{t_{A}}\right)}^{N-1}\left(\frac{N-1}{t_{A}}\right). \end{array}$$

The summation of these two terms is:


(8) $$\begin{array}{l} \frac{\partial }{\partial t_{N}}f\left(t_{N},\;t_{A}\right)={\left(1-\frac{t_{N}}{t_{A}}\right)}^{N-2}\left(\frac{N-1}{t_{A}}\right)\left(1-N\frac{t_{N}}{t_{A}}\right). \end{array}$$

Since the local maxima of *f*(*t*_*N*_, *t*_*A*_) can be taken when $\frac{\partial }{\partial t_{N}}f\left(t_{N},\;t_{A}\right)=0$, the possible values are *t*_*N*_ = *t*_*A*_ or *t*_*N*_ = *t*_*A*_/*N*. Since *f*(*t*_*N*_, *t*_*A*_) = 0 when *t*_*N*_ = *t*_*A*_ and *f*(*t*_*N*_, *t*_*A*_) > 0 when *t*_*N*_ = *t*_*A*_/*N*, the likelihood function *f*(*t*_*N*_, *t*_*A*_) has the maximum value at (Figure 3A):


(9) $$\begin{array}{l} t_{N}=\frac{t_{A}}{N}. \end{array}$$

This solution for the maximization of *f*(*t*_*N*_, *t*_*A*_) in Equation 9 needs to satisfy the necessary conditions 0 < *t*_*N*_ ≤ *t*_*A*_/(*N* − 1) (Equation 2). If *t*_*A*_ of a cell assembly is fixed, the optimal information transmission delay *t*_*N*_ decreases as the size of the loop structure *N* increases. With this optimal information transmission delay *t*_*N*_, the maximum likelihood in this structure is:


(10) $$\begin{array}{l} \max f\left(t_{N},\;t_{A}\right)={\left(1-\frac{1}{N}\right)}^{N}. \end{array}$$

The maximum likelihood, max*f*(*t*_*N*_, *t*_*A*_), increases as the size of this structure *N* increases and soon saturates to *e*^−1^≈0.3679 (Figure 3C).

If we assume that *t*_*N*_, *t*_*A*_, and *t*_*I*_ are heterogeneous for *N* different brain areas, the likelihood is the product of different *N*-terms so as to make it difficult to obtain a simplified mathematical solution. In Equation 4, we would have $\left(\prod_{i=1}^{N-1}1-\frac{t_{N,i}}{t_{A,i}}\right)\frac{\left(N-1\right)t_{N,N}}{t_{A,N}}$ instead of ${\left(1-\frac{t_{N}}{t_{A}}\right)}^{N-1}\frac{\left(N-1\right)t_{N}}{t_{A}}$. However, in this case, the number of similar terms with the same characteristics increases, so it does not have a significant impact on the conclusions of this study, especially when areal heterogeneity is not large.

## 3 Simulation methods

### 3.1 Simulation to derive the basic neural activity model

To derive the basic neural activity model, we performed the simulation of a neuronal network. All parameters used in this simulation were provided in Table 1. The neuronal network was based on the excitatory-inhibitory balanced networks and the flow of postsynaptic potential (PSP) changes. Neurons in the network belonged to either the excitatory E, inhibitory I, or external Ext populations. The ratio 4:1 of E to I was in accordance with the previous simulation study (Litwin-Kumar and Doiron, 2014). The synaptic inputs from E made the excitatory postsynaptic potential (EPSP), while the synaptic inputs from I made the inhibitory postsynaptic potential (IPSP). The PSP of excitatory neuron *i* was determined as:


(11) $$\begin{array}{l} \frac{d}{dt}V_{i}^{E}\left(t\right)=-\frac{1}{t_{C}}V_{i}^{E}\left(t\right)+g_{i}^{Ext}\left(t\right)+g_{i}^{EE}\left(t\right)-g_{i}^{EI}\left(t\right) \end{array}$$

where *t*_*C*_ is the membrane time constant, $g_{i}^{Ext}\left(t\right)$ is an external input, $g_{i}^{EE}\left(t\right)$ is an excitatory input, and $g_{i}^{EI}\left(t\right)$ is an inhibitory input. The PSP of inhibitory neuron *i* was determined as:


(12) $$\begin{array}{l} \frac{d}{dt}V_{i}^{I}\left(t\right)=-\frac{1}{t_{C}}V_{i}^{I}\left(t\right)+g_{i}^{IE}\left(t\right)-g_{i}^{II}\left(t\right) \end{array}$$

where $g_{i}^{IE}\left(t\right)$ is an excitatory input, and $g_{i}^{II}\left(t\right)$ is an inhibitory input. Then the spiking activity of an E or I neuron was determined as:


(13) $$\begin{array}{c} A_{i}^{P}\left(t\right)=\;\{\begin{array}{cc} 1,\;if\;V_{i}^{P}\left(t\right)>0.2\; & \;\\ \; & \;\\ \; & \;\\ \; & and\;u>0.995\\ 0,\;otherwise & \; \end{array} \end{array}$$

where *P* is a population (E or I) and *u* is a pseudo-random number generated from the uniform distribution between 0 and 1. This was intended to generate irregular spike patterns reflecting probabilistic neural responses (Cannon et al., 2010). Once a neuron was active (1), it became inactive (0) for the next 3 ms, representing the refractory period of the action potential of the neurons. The spiking activity of Ext neuron was determined as:


(14) $$\begin{array}{c} A_{i}^{Ext}\left(t\right)=\;\{\begin{array}{c} 1,\;if\;w>0.125\\ 0,\;otherwise \end{array} \end{array}$$

where *w* is a pseudo-random number generated from the uniform distribution between 0 and 1. The synaptic kernel, which determines the temporal properties of PSP, can be modeled as follows (Litwin-Kumar and Doiron, 2014):


(15) $$\begin{array}{l} K^{P}\left(t\right)=\frac{1}{\tau_{d}^{P}-\tau_{r}^{P}}\left(\exp\left(-\frac{t}{\tau_{d}^{P}}\right)-\exp\left(-\frac{t}{\tau_{r}^{P}}\right)\right) \end{array}$$

where *P* is excitatory population *E* or inhibitory population *I*, $\tau_{r}^{P}$ is the rise time for synapses of the certain population *E* or *I*, and $\tau_{d}^{P}$ is the decay time for synapses of the certain population *E* or *I*. The current PSP is a convolution between the synaptic kernel *K*^*P*^(*t*) and previous neural activity. The rise and decay times of the current PSP is determined by $\tau_{r}^{P}$ and $\tau_{d}^{P}$. The synaptic input from E or I neurons was the weighted sum of the spiking activities multiplied by the synaptic kernel as follows:


(16) $$\begin{array}{l} g_{i}^{XP}\left(t\right)=K^{P}{\left(t\right)}^{*}\underset{j}{\sum }J_{ij}^{XP}A_{j}^{P}\left(t\right) \end{array}$$

where *P* is a certain population, E or I, *K*^*P*^(*t*) is the synaptic kernel introduced in Equation 15, $J_{ij}^{XP}$ is the synaptic efficacy from P to E population (Table 1), and ^*^ is a convolution operator. The parameters used in the synaptic kernel were similar to the previous study (Litwin-Kumar and Doiron, 2014). The ratio of E-I to E-E synaptic efficacy was set to 4 (Table 1). This balance of the excitatory-inhibitory populations used in the simulation was similar to the previous study, which represents strong lateral inhibition (Sadeh and Clopath, 2021). The synaptic input from Ext to E neurons was as follows:


(17) $$\begin{array}{l} g_{i}^{Ext}\left(t\right)=\underset{j}{\sum }J_{ij}^{Ext}A_{j}^{Ext}\left(t\right) \end{array}$$

where $J_{ij}^{Ext}$ is the synaptic efficacy from Ext to E population (Table 1).

**Table 1:** Parameters for simulation to derive basic neural activity model.

**Symbol**	**Description**	**Value**
*N*_*E*_	Number of excitatory (E) neurons	160
*N*_*I*_	Number of inhibitory (I) neurons	40
*N*_*Ext*_	Number of external (Ext) neurons	160
*J* ^*EE*^	Synaptic efficacy from E to E	[0, 1] × 0.8/*N*_*E*_
*J* ^*EI*^	Synaptic efficacy from I to E	[0, 1] × 3.2/*N*_*E*_
*J* ^*IE*^	Synaptic efficacy from E to I	[0, 1] × 3.2/*N*_*I*_
*J* ^*II*^	Synaptic efficacy from I to I	[0, 1] × 3.2/*N*_*I*_
*J* ^*Ext*^	Synaptic efficacy from Ext to E	[0, 1] × 0.7
*P*_*Conn*_	Connection probability between neurons	0.2
τ_*C*_	Membrane time constant	20 ms
τ_*ref*_	Refractory period	3 ms
τ_*sim*_	Simulation time resolution	0.1 ms
$\tau_{r}^{E}$	Rise time for E synapse	1 ms
$\tau_{d}^{E}$	Decay time for E synapse	6 ms
$\tau_{r}^{I}$	Rise time for I synapse	0.5 ms
$\tau_{d}^{I}$	Decay time for I synapse	2 ms

### 3.2 Simulation for the learning and the retrieval of sequential memory

For simplicity, we set the size of the loop structure to *N* = 3, in addition, *N* = 2, 4, 5, 6. The initiation period range was set to *t*_*N*_ = [20, 60] ms, which was comparable to the previous study (Siegle et al., 2021; for a review, Wang, 2022). The range of initiation period was distributed around the biologically plausible initiation period of 40 ms. The active period was set to *t*_*A*_ = 120−*t*_*N*_ ms, which is equal to or longer than the range of the initiation period. The inhibition period range was set to *t*_*I*_ = [0, 120] ms, which were also within a reasonable range compared to the range of the initiation period to cover both the initiation and active periods. These lengths of the initiation, active, and inhibition periods, *t*_*N*_, *t*_*A*_, and *t*_*I*_, respectively, were the parameters of our basic neural activity model. In spiking neural networks, the lengths of the initiation, active, and inhibition periods depend on other parameters such as time constant, synaptic efficacy, spiking threshold, and also varies across brain areas. However, here, a range of three periods was used to verify the theoretical predictions in Section 2. In these three parameter ranges, there are parameters satisfying the necessary conditions up to *N* = 2, … , 6. Therefore, simulations were performed at these structure sizes.

We modeled one cell assembly by one basic neural activity model, which constituted a fundamental unit in the simulation. Here, unit refers to cell assembly. The value of a cell assembly was 1 for the active period or 0 for the initiation or inhibition periods. It simplifies the activity of cell assemblies as 1 or 0. These values are used to calculate Hebbian plasticity described in Equation 1. We set five cell assemblies to represent a common event in each area. Here, event refers to an external event and is represented by the activities, e.g., *X*_*t*_ and *Y*_*t*_ in Section 2, of cell assemblies. In this simulation, *X*_*t*_ and *Y*_*t*_ are pre-assigned to represent each external event such that all external events are represented as disjoint sets, e.g., *X*_*t*_ and *X*_*t*+1_, of the same number of cell assemblies, with each set assigned to appear at a specific time delay. The five cell assemblies in the same area had the same cycle of periods with the same starting point. We also assumed that different events were represented distinctly by different cell assemblies. As we presented ten consecutive events to the loop structure, a total of fifty cell assemblies were created in each area. Ten consecutive events were applied to each area with sequentially added time delays. Time delays indicate the size of the initiation period, reflecting a situation in which external events are sequentially represented in the brain.

When ten consecutive neural representations were induced by external events in the first area, the same events were presented in the second area with a delay of *t*_*N*_. Synaptic weights between the cell assemblies of connected areas were learned by Hebbian plasticity where a synaptic weight was strengthened if both pre- and post-synaptic neurons were activated. The sum of all synaptic weights on a cell assembly was fixed to implement the synaptic normalization during 1,000 iterations of the presentation of ten events. The value of each cell assembly was set to 1 if the weighted sum of the values of cell assemblies in other areas and the corresponding synaptic weights exceeded 0.15; otherwise, it was set to 0.

A difference between the assumptions in theory and simulation was the distribution of the phase of the cell assembly within an area. While theory assumes that the phases of assembly activity are randomly distributed, simulation assumes that all assembly activities are in the same phase to quantify the results.

### 3.3 Simulation for the retrieval of sequential memory in spiking neural networks

For consistency with the simulations in the previous section, we set the size of the loop structure to *N* = 2, …, 6, containing two to six brain areas. Neurons in the network belonged to either the excitatory E or inhibitory I populations. We employed an adaptive exponential leaky integrate-and-fire neuron model (Brette and Gerstner, 2005) as the spiking neuron model and adopted model parameters from the previous study (Litwin-Kumar and Doiron, 2014). All parameters used in these simulations were provided in Table 2.

**Table 2:** Parameters for spiking neural network simulations.

**Symbol**	**Description**	**Value**
*N*_*E*_	Number of excitatory (E) neurons in each area	500
*N*_*I*_	Number of inhibitory (I) neurons in each area	125
$R_{A}^{E}$	E resting potential	−70 mV
$R_{A}^{I}$	I resting potential	−62 mV
$R_{B}^{E}$	E reversal potential	0 mV
$R_{B}^{I}$	I reversal potential	−75 mV
δ_θ_	Slope factor	2 mV
*P*_*Conn*_	Connection probability between neurons	0.2
τ_*C*_	Membrane time constant	20 ms
τ_*ref*_	Refractory period	1 ms
*C*	Capacitance	300 pF
τ_θ_	Threshold time scale	30 ms
*V*_θ_	Threshold potential	−52 mV
τ_*W*_	Spike-triggered adaptation time scale	150 ms
*c*_*W*_	Subthreshold adaptation	4 nS
*b*_*W*_	Adaptation current increase	0.805 pA
*V*_*upper*_	Upper bound potential	20 mV
*V*_*lower*_	Lower bound potential	−60 mV
*J* ^*EE*^	Synaptic efficacy from E to E	0–30 pF
*J* ^*EI*^	Synaptic efficacy from I to E	0–2,500 pF
*J* ^*IE*^	Synaptic efficacy from E to I	0–20 pF
*J* ^*II*^	Synaptic efficacy from I to I	0–250 pF
*J* ^*Ext*^	Synaptic efficacy from Ext to E	1.78 pF
$\tau_{r}^{E}$	Rise time for E synapse	7–25 ms
$\tau_{d}^{E}$	Decay time for E synapse	12.6–45 ms
$\tau_{r}^{I}$	Rise time for I synapse	1.5–15 ms
$\tau_{d}^{I}$	Decay time for I synapse	6–60 ms

All the equations and parameters used in the spiking neural network model were taken from Litwin-Kumar and Doiron (2014). Specifically, the dynamics $V_{i}^{E}\left(t\right)$ of the potential of E neuron *i* at time *t* was as follows:


(18) $$\begin{array}{l} \begin{array}{c} \frac{d}{dt}V_{i}^{E}\left(t\right)=\frac{1}{\tau_{C}}\left(R_{A}^{E}-V_{i}^{E}\left(t\right)+\delta_{\theta }e^{\frac{V_{i}^{E}\left(t\right)-V_{\theta ,i}\left(t\right)}{\delta_{\theta }}}\right)\\ +\frac{g_{i}^{EE}\left(t\right)}{C}\left(R_{B}^{E}-V_{i}^{E}\left(t\right)\right)+\frac{g_{i}^{EI}\left(t\right)}{C}\left(R_{B}^{I}-V_{i}^{E}\left(t\right)\right)-\frac{W_{i}\left(t\right)}{C} \end{array} \end{array}$$

where *V*_θ, *i*_(*t*) is the threshold dynamics, $g_{i}^{EE}\left(t\right)$ and $g_{i}^{EI}\left(t\right)$ are the E to E and I to E synaptic conductance, respectively, and *W*_*i*_(*t*) is the adaptation current. The dynamics $V_{i}^{I}\left(t\right)$ of the potential of I neuron *i* at time *t* was as follows:


(19) $$\begin{array}{l} \begin{array}{c} \frac{d}{dt}V_{i}^{I}\left(t\right)=\frac{1}{\tau_{C}}\left(R_{A}^{I}-V_{i}^{I}\left(t\right)+\delta_{\theta }e^{\frac{V_{i}^{I}\left(t\right)-V_{\theta ,i}\left(t\right)}{\delta_{\theta }}}\right)\\ +\frac{g_{i}^{IE}\left(t\right)}{C}\left(R_{B}^{E}-V_{i}^{I}\left(t\right)\right)+\frac{g_{i}^{II}\left(t\right)}{C}\left(R_{B}^{I}-V_{i}^{I}\left(t\right)\right) \end{array} \end{array}$$

where $g_{i}^{IE}\left(t\right)$ and $g_{i}^{II}\left(t\right)$ are the E to I and I to I synaptic conductance, respectively. The threshold dynamics *V*_θ, *i*_(*t*) was given as follows:


(20) $$\begin{array}{l} \frac{d}{dt}V_{\theta ,i}\left(t\right)=\frac{1}{\tau_{\theta }}\left(V_{\theta }-V_{\theta ,i}\left(t\right)\right). \end{array}$$

An E neuron was influenced by the dynamics of the adaptation current *W*_*i*_(*t*) given by:


(21) $$\begin{array}{l} \frac{d}{dt}W_{i}\left(t\right)=\frac{1}{\tau_{W}}\left(c_{W}\left(V_{i}^{E}\left(t\right)-R_{A}^{E}\right)-W_{i}\left(t\right)\right). \end{array}$$

A neuron discharged a spike when its potential increased above *V*_*upper*_. At the same time, *W*_*i*_(*t*) was increased by *b*_*w*_. After spiking, the potential was set to *V*_*lower*_ during the refractory period.

The dynamics $g_{i}^{XP}\left(t\right)$ of the synaptic conductance from population *P* to *X* at time *t* was given as follows:


(22) $$\begin{array}{l} g_{i}^{XP}\left(t\right)={\left(J_{ext}^{XP}A_{ext}^{XP}\left(t\right)+\underset{j}{\sum }J_{ij}^{XP}A_{j}^{P}\left(t\right)\right)}^{*}K^{P}\left(t\right) \end{array}$$

where *P* and *X* are E or I, $J_{ext}^{YX}$ is the synaptic efficacy from external neurons, $J_{ij}^{XP}$ is the synaptic efficacy from population *P* to population *X*, $A_{ext}^{XP}\left(t\right)$ is the number of spikes of external neurons, $A_{j}^{X}\left(t\right)=1\;or\;0$ represents spiking activity of neuron *j*, ^*^ is the convolution, and *K*^*P*^(*t*) is the synaptic kernel in Equation 16. The specific parameters of the synaptic kernel were $\tau_{r}^{E}$, $\tau_{d}^{E}$, $\tau_{r}^{I}$, and $\tau_{d}^{I}$, which indicate the rise *r* and decay *d* time for synapses of the *E* and *I* populations, respectively. These parameters were adjusted to control the initiation and inhibition periods of the simulation (see Table 2). Consequently, the initiation duration, representing the appearance of the first spike, fell within the range [25.5, 55.5] *ms*. An approximate inhibition duration was calculated as $2\times \left(\tau_{r}^{I}+\tau_{d}^{I}\right)$, resulting in a range of [15, 150] *ms*.

We presented 10 consecutive events within the loop structure, and the synaptic weights were preset to ensure that this sequence of events was self-generating. There were 10 assemblies in each area. E-I, I-E, and I-I populations were connected only within each assembly. The E-E populations were not only connected within an assembly in an area, but also connected between areas. The E-E connections from the *n*th to the (*n* + 1)-th areas had similar structures, while the E-E connection matrix from the (*N* + 1)-th to the first area was shifted circularly downward by 50 rows—one assembly—along the vertical axis to enable the self-generation of spiking activity. The external stimulus was applied only to the first assembly in each area. The external stimulus was given during 0–120 ms to the first area, 40–160 ms to the second area and 80–200 ms to the third area, with a 40-ms delay between subsequent areas. The spiking activity could then propagate sequentially to other assemblies, resulting in self-generation.

There exists a trial-to-trial variability in neural activity, which can be viewed as spontaneous neuronal noise. It is therefore important to know whether the sequential retrieval of “Self-generation” would be affected in the presence of such spontaneous neuronal noise. To confirm this, we re-performed the simulation of the spiking neural network with an initiation period = 39.5 ms and inhibition period = 105 ms. However, at this time, neuronal noise of various magnitudes, i.e., 0–4/6 noise level, was applied to 10% of randomly selected neurons. We set an external stimulus as 12-kHz spikes with the synaptic efficacy of *J*^*Ext*^. We also generated external noise as noise level × 12-kHz spikes with the same synaptic efficacy. Note that the magnitude of external stimulus and noise represent the aggregation of the firing rates of all presynaptic neurons. For the issue of the aggregation of the firing rates of different neurons, we assumed that the firing processes of neurons are mutually independent Poisson processes. Due to this assumption, the firing rate of different neurons can be added together linearly. Both the external stimulus and external noise were input to a fixed set of neurons or a set of randomly selected neurons, respectively, each of which occupied 10% of the whole neurons. If the noise level is equal to 1/6, the noise is provided to the same number of neurons as those receiving the stimulus but with as 1/6 less intensity as that of the stimulus.

## 4 Simulation results

### 4.1 Simulation results for the learning and the retrieval of sequential memory

**Figure 4:**
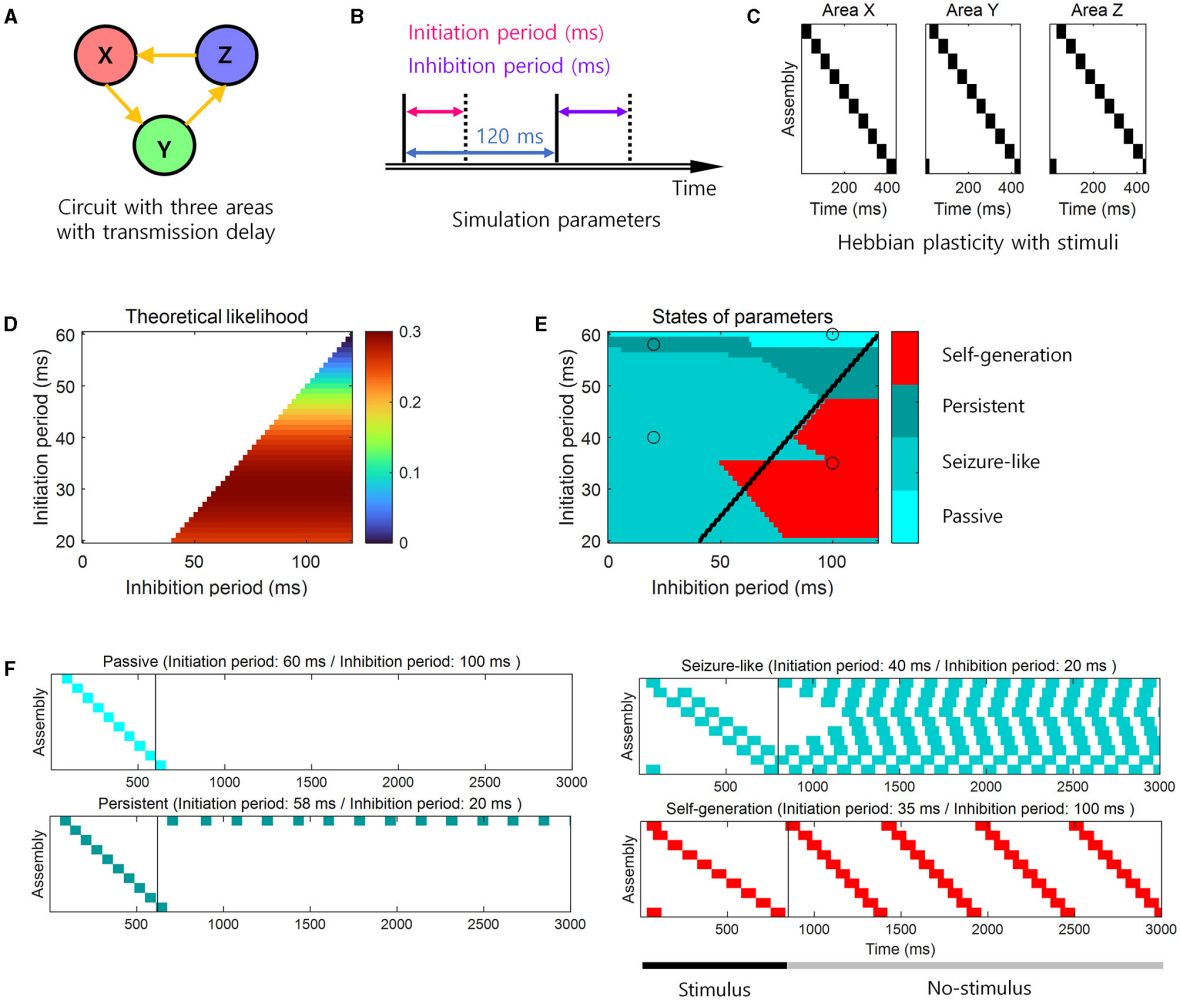
Simulation for the learning and the retrieval of sequential memory. **(A)** The loop structure. **(B)** Simulation parameters for the basic neural activity model. The active period was set to 120 ms minus the length of the initiation period. **(C)** Stimuli that exhibit neural representations to the learning via Hebbian plasticity. **(D)** The theoretical prediction of the learning of sequential memory for simulation parameters. The color-coded parameter indicates that this parameter satisfies the necessary conditions for the learning of sequential memory (see Equations 2, 3). The color codes the likelihood of the learning of sequential memory (see Equation 4). **(E)** The simulation results for the theoretical prediction. The color codes the 4 states of neural activity. We determined the state in the simulation in the following way. The “Passive” state was determined if there was no active cell assembly when the stimulus ended, representing the absence of activity. The “Seizure-like” state was determined if more than 40% of all cell assemblies were active when the stimulus ended, representing the excessive activity. If the active cell assemblies existed and occupies <40% when the stimulus ended, the state was determined as the “Self-generation” or “Persistent” state. Among these two states, if cell assemblies were activated by the order of stimuli, then the state was determined as “Self-generation”, representing the generation of the stimulus sequence without the stimulus presentation. Otherwise, the state was alternatively determined as “Persistent”, representing the incapability of generating the stimulus sequence. Black line represents the boundary of areas that satisfy the necessary conditions. **(F)** Examples of four states of neural activity. The left side of the black vertical bar indicates the neural activity during the stimulus presentation. The right side of the black vertical bar indicates the neural activity during the no-stimulus presentation. Each panel corresponds to the black open circle in e.

To confirm the theoretical predictions, we performed the simulation study when *N* = 3 (Figures 4A–C). When the theoretical likelihood (Equation 4) was high within the range of the parameters that satisfied the necessary conditions (Equations 2, 3), the simulation result showed that the neural representations were self-generated and sequential memory emerged, i.e., “Self-generation” (Figure 4F). When the theoretical likelihood was low within the range that satisfied the necessary conditions, cell assemblies responded only when a stimulus was given, i.e., “Passive” (Figure 4F). In the parameter range where necessary conditions were not satisfied, the last stimulated cell assemblies continued to respond, i.e., “Persistent” (Figure 4F), or all cell assemblies responded, i.e., “Seizure-like” (Figure 4F). These simulation results confirmed the theoretical predictions for the learning of sequential memory (Figures 4D–F). Specifically, self-generation of sequential activities mainly occurred within a parameter range where the theoretical likelihood was >0.2 while satisfying the necessary conditions (Figures 4D,E). For example, when the initiation period was 35 ms and the inhibition period was 100 ms, we observed “Self-generation” (Figure 4F). In contrast, when the inhibition period was smaller, e.g., 20 ms, but with the similar initiation period, the necessary conditions were not satisfied, resulting in the “Seizure-like” state (Figure 4F). Additionally, when the initiation period was 60 ms and the inhibition period was 100 ms, the cell assemblies exhibit “Passive” state (Figure 4F). When the inhibition period was 58 ms and the necessary conditions were not met, the cell assemblies were in “Persistent” state (Figure 4F).

**Figure 5:**
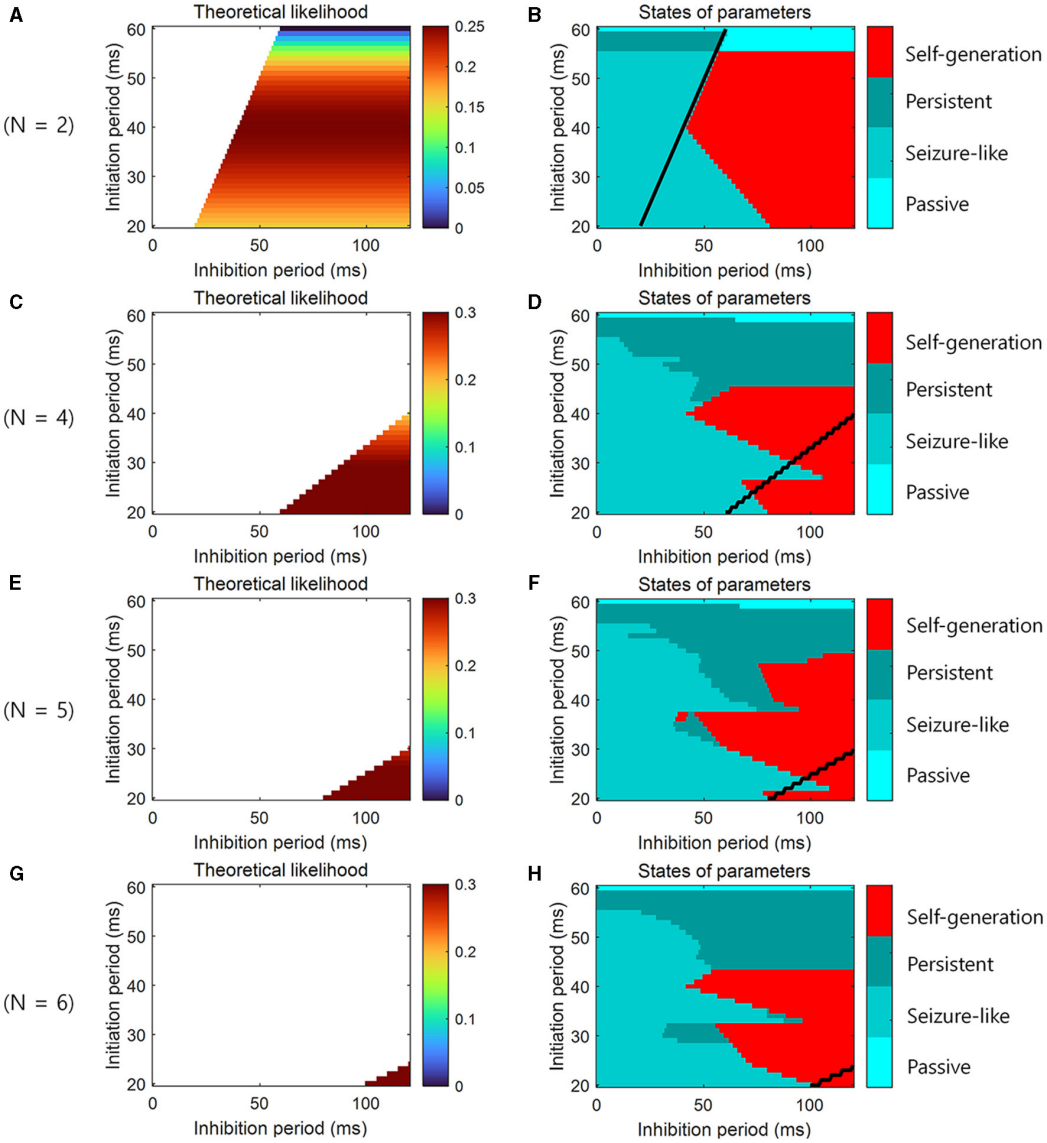
Simulation for the learning and the retrieval of sequential memory in loop structures of various sizes. **(A)** The theoretical prediction of the learning of sequential memory for simulation parameters when *N* = 2. The color-coded parameter indicates that this parameter satisfies the necessary conditions for the learning of sequential memory (see Equations 2, 3). The color codes the likelihood of the learning of sequential memory (see Equation 4). **(B)** The simulation results for the theoretical prediction when *N* = 2. The color codes the 4 states of neural activity. **(C)** Similar to **(A)**, but *N* = 4. **(D)** Similar to **(B)**, but *N* = 4. **(E)** Similar to **(A)**, but *N* = 5. **(F)** Similar to **(B)**, but *N* = 5. **(G)** Similar to **(A)**, but *N* = 6. **(H)** Similar to **(B)**, but *N* = 6. We determined the state in the simulation in the same way as for Figure 4E. Black line represents the boundary of areas that satisfy the necessary conditions.

To confirm theoretical predictions in loop structures of different sizes, additional simulations were performed not only at *N* = 3 but also at *N* = 2, 4, 5, 6. When the theoretical likelihood (Equation 4) was high within the range of the parameters that satisfied the necessary conditions (Equations 2, 3), the simulation result showed that the neural representations were self-generated, and sequential memory emerged (Figures 5A–H).

Note that our theoretical model assumes that neural activity has a random phase such that every cell assembly in an area changes its activity exactly once during *t*_*A*_ (see Section 2.3). However, in the simulations, only a few cell assemblies were activated simultaneously; otherwise they displayed seizure-like behavior. Therefore, for the sake of visualization and effective simulations, this assumption is not always met. That is, it may be possible to change the neural activity of every cell assembly before *t*_*A*_. Conversely, the neural activity of cell assemblies can be unchanged during as well as after *t*_*A*_ due to prolonged lack of activity. Let α be such timing variation. In the former case, α has a negative value; in the latter, α has a positive value. The conditions to obtain Equation 2 are then modified as follows: *Nt*_*N*_ ≤ *t*_*N*_ + *t*_*A*_ + α. This condition yields:


(23) $$\begin{array}{l} \begin{array}{c} t_{N}\leq \frac{t_{A}+\alpha }{N-1}. \end{array} \end{array}$$

The conditions to obtain Equation 3 can be modified as follows: *Nt*_*N*_ + *t*_*A*_ ≤ *t*_*N*_ + *t*_*A*_ + *t*_*I*_ + α. This condition yields:


(24) $$\begin{array}{l} t_{N}\leq \frac{t_{I}+\alpha }{N-1}. \end{array}$$

This is why “self-generation” appears in the simulation (Figures 4E, 5A–H), although it does not satisfy the necessary conditions (Equations 2, 3).

### 4.2 Simulation results for the retrieval of sequential memory in spiking neural networks

**Figure 6:**
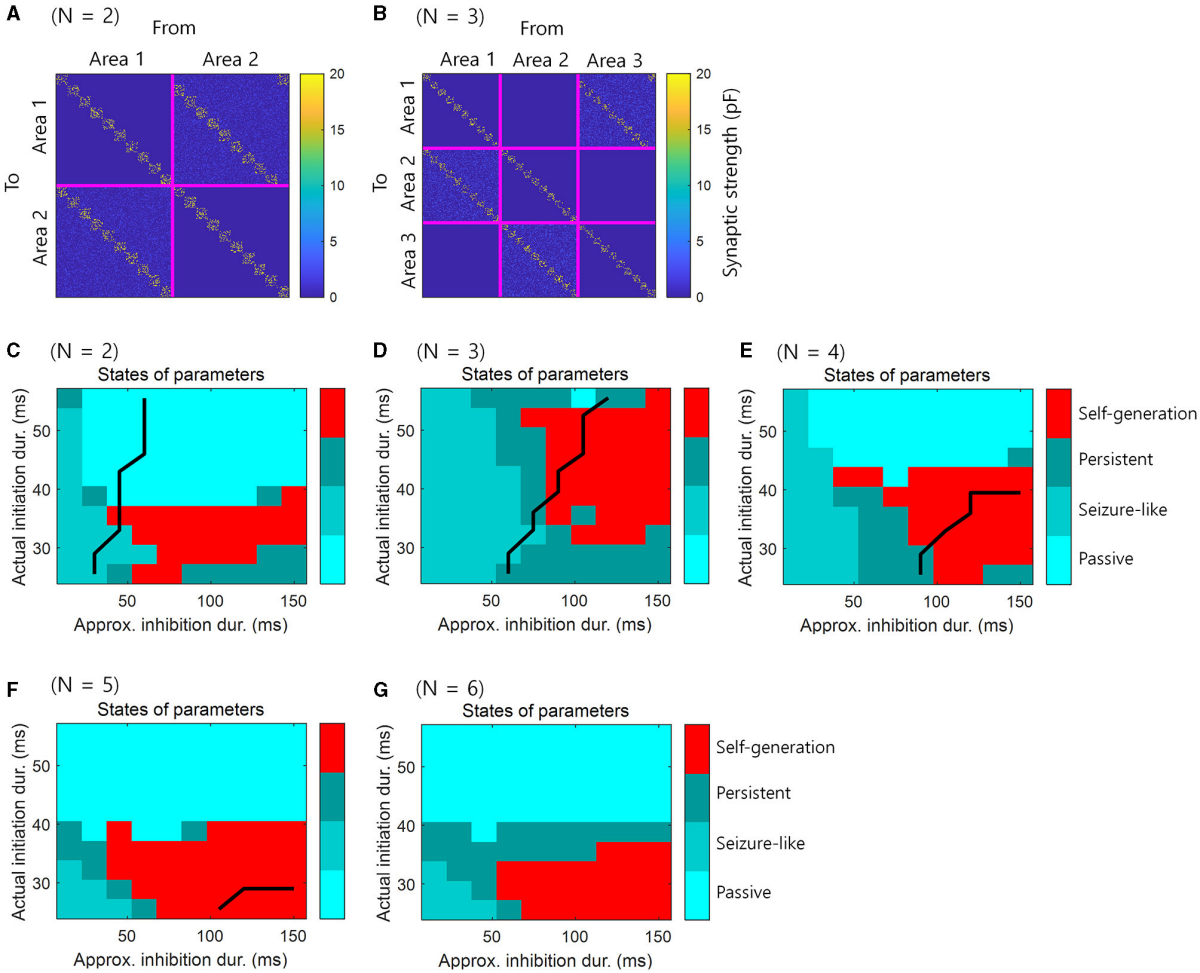
Simulation for the retrieval of sequential memory in loop structures of various sizes in spiking neural networks. **(A)** Pre-assigned synaptic weights for simulation of retrieval of sequential memory in spiking neural networks when *N* = 2. **(B)** Similar to **(A)** with *N* = 3. **(C)** The simulation results in spiking neural networks when *N* = 2. **(D)** Similar to **(C)**, but *N* = 3. **(E)** Similar to **(C)**, but *N* = 4. **(F)** Similar to **(C)**, but *N* = 5. **(G)** Similar to **(C)**, but *N* = 6. Black line represents the boundary of areas that satisfy the necessary conditions when *t*_*A*_ = 120 ms virtually. The right-lower areas of black line satisfy the necessary conditions.

For consistency with the simulations in the previous section, we set the size of the loop structure to *N* = 2, …, 6, containing two to six brain areas. We presented 10 consecutive events within this structure, and the synaptic weights were preset to ensure that this sequence of events was self-generating. There were 10 assemblies in each area and one assembly contains 50 neurons. The E-E populations were not only connected within assembly, but also connected between areas. The E-E connections from *n* to *n* + 1 areas had similar structures, while, the E-E connection from *N* + 1 to 1 area was shifted by one assembly to enable the self-generation of spiking activity (Figures 6A, B). The external stimulus was applied during 120 ms to the first assembly in each area, with a 40 ms delay added cumulatively to each area. The spiking activity could then propagate sequentially to other assemblies, resulting in self-generation.

What differs from previous simulations is that we controlled the initiation period and inhibition period by adjusting the parameters of the synaptic kernel. The specific parameters of the synaptic kernel were $\tau_{r}^{E}$, $\tau_{d}^{E}$, $\tau_{r}^{I}$, and $\tau_{d}^{I}$, which indicate the rise *r* and decay *d* time for synapses of the *E* and *I* populations. These parameters were adjusted to control the initiation and inhibition periods of this simulation (see, Table 2). Consequently, the actual initiation duration, representing the appearance of the first spike, fell within the range [25.5, 55.5] *ms*. An approximate inhibition duration was calculated as $2\times \left(\tau_{r}^{I}+\tau_{d}^{I}\right)$, resulting in a range of [15, 150] *ms*. Unlike previous simulations, the longer the initiation period was, the longer the active period was in this simulation. The purpose of this simulation is not to use a simplified basic neural activity model, but to use spiking neural networks in a real environment to verify “Self-generation”.

Simulation results showed that the phenomenon of “self-generation” was also evident in spiking neural networks of loop structure with various sizes *N*. We assumed that the actual initiation duration corresponds to the initiation period and the approximated inhibition duration corresponds to the inhibition period. The simulation results demonstrated that when the theoretical likelihood was high within the range of parameters satisfying the necessary conditions (Figures 4D, 5A, C, E, G), neural representations were self-generated, indicating the retrieval of sequential memory (Figures 6C–G).

**Figure 7:**
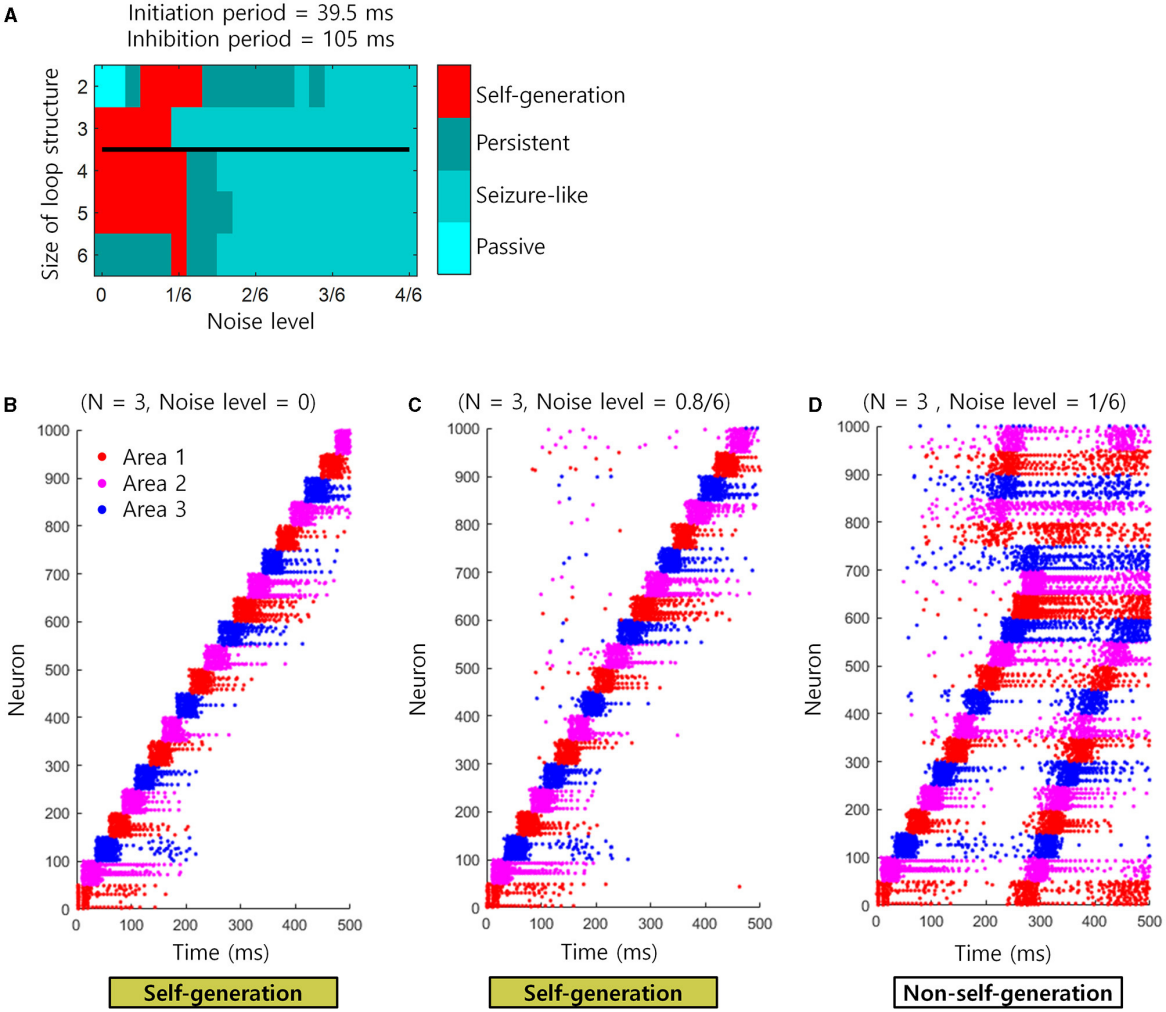
Simulation for noise injection in spiking neural networks. **(A)** The state of the loop structures (self-generation, seizure-like, persistent or passive) depending on the noise level and the size of loop structures. Black line represents the boundary of areas that satisfy the necessary conditions when *t*_*A*_ = 120 ms virtually. The upper areas of black line satisfy the necessary conditions. **(B)** An example of spike trains when the noise level is 0. Each colored dot indicates each spike. Red dots indicate spikes of neuron in the area 1. Magenta dots indicate spikes of neuron in the area 2. Blue dots indicate spikes of neuron in the area 3. **(C)** Similar to **(B)**, but the noise level is 0.8/6. **(D)** Similar to **(B)**, but the noise level is 1/6.

There exists a trial-to-trial variability in neural activity, which can be viewed as spontaneous neuronal noise. It is therefore important to know whether the sequential retrieval of “Self-generation” would be affected in the presence of such spontaneous neuronal noise. To confirm this, we re-performed the simulation of the spiking neural network with an initiation period = 39.5 ms and inhibition period = 105 ms. However, at this time, neuronal noise of various magnitudes, i.e., 0–4/6 noise level, was applied to 10% of randomly selected neurons. As a result, the “self-generation” of the sequence was retrieved well until the noise level was ≤ 1/6, but disrupted when the noise level exceeded 1/6 (Figure 7A). This phenomenon may occur because as the magnitude of the noise increases beyond a 1/6 level, the cell assembly can be activated regardless of the sequence (Figures 7B–D). That is, below a 1/6 noise level, spontaneous neuronal noise can be corrected and ignored by the loop structure, but above that level, these errors appear to propagate globally (Figures 7C, D). See the previous section for more information about applying noise.

## 5 Discussion

In the present study, we investigated how brain loop structures subserve the learning of sequential memory. We assumed that the sequential memory emerges by delayed information transmission in these structures (Figure 1) and presented a basic neural activity model for the delayed information transmission (Figure 2). Based on this model, we described necessary conditions for the learning of sequential memory in these structures (Figure 3). Through the simulation, it was confirmed that sequential memory emerged in this structure under the theoretically predicted conditions and the neural representations of sequential events were self-generated (Figure 4).

If the active or inhibition periods of a cell assembly are fixed, the possible range of information transmission delay is bounded by the inverse of the size of the loop structure. In other words, as the size of this structure increases, the information transmission delay should decrease (Equations 2, 3; Figures 3A, D). The range of information transmission delay may be bounded according to the cellular properties of the tissue. Therefore, the information transmission delay cannot be infinitely reduced. This means that for the learning of sequential memory, (1) the size of brain loop structure must be limited so that it does not become too large. The optimal likelihood of the learning of sequential memory increases as the size of this structure increases and soon saturates theoretically (Equation 10; Figure 3C). Yet, it is noteworthy that it remains challenging to verify these characteristics of the optimal likelihood by simulations because while the possibility of sequential memory learning can be confirmed through simulation, it is difficult to determine “how well sequential memory is learned” by simulations. This means that for the learning of sequential memory, (2) the size of brain loop structure must be relatively large, but not necessarily infinitely large. Combining (1) and (2), we come to a conclusion that a moderate level of this structure size, e.g., *N* = 5, is advantageous for the learning of sequential memory. This may explain why biological brain loop structures are made up of a moderate number of areas, such as cortico-basal ganglia-thalamic loops (Alexander et al., 1986; Lee et al., 2020; Foster et al., 2021) and cortico-cerebellar loops (Middleton and Strick, 2000; Kelly and Strick, 2003; Ramnani, 2006).

The basic neural activity model in this study is based on cell assemblies. During the inhibition and initiation periods, cell assemblies exhibit low activity levels, whereas they exhibit high activity levels during the active period, indicating oscillatory patterns. If the lengths of the inhibition plus initiation periods and the active period are unequal (Figures 4, 5), they can resemble sawtooth oscillations, similar to those observed in hippocampal theta oscillations (Montgomery et al., 2009).

The firing timing of hippocampal neurons is known to be related to the oscillatory phase of theta oscillations, and the theta sequence—the order of neuron firing timings associated with theta oscillations—is known to correlate with behavioral sequences (Foster and Wilson, 2007; Gupta et al., 2012). This exemplifies how spatially and temporally correlated neural activity, i.e., oscillations, is linked to sequences of behaviors.

In our model, if the activities of cell assemblies are oscillatory and spatially correlated, the neural activity represented by the active period spreads spatially like a traveling wave. In this case, because neural activity propagates collectively, the sequential activation of cell assemblies is less likely to be disrupted and may therefore appear more stable. These oscillatory propagations are thought to facilitate information transmission across many brain areas and frequency bands (Rubino et al., 2006; Bhattacharya et al., 2022; Zabeh et al., 2023; for reviews, see Bauer et al., 2020). This collective information transmission through loop structures enables stable sequential activation of cell assemblies, allowing the brain to better represent behavioral sequences.

Recent studies showed that a sequence of activities can be generated in randomly connected structures (Rajan et al., 2016; Rajakumar et al., 2021), other than loop structures. The present study does not exclude this possibility. Moreover, it has been shown that the neural representations of sequential events can be self-generated even when input is absent in recurrent structures (Klos et al., 2018). However, a key difference between the present study and other studies is whether it handles loop structures, which are known to be important in sequential memory.

The present study used a basic model to simplify the derivation of mathematical results, and therefore, all connections between each area were assumed to be excitatory. However, inhibitory connections are also present in cortico-basal ganglia-thalamic loops (Lanciego et al., 2012). Our results can still be applied to inhibitory connections if cell assemblies of each area selectively represent each neural representation. However, it is a limitation of this study that the diversity of these excitatory-inhibitory connections was not directly incorporated into the model. This point can be addressed in future studies.

There exists a trial-to-trial variability in neural activity, which can be viewed as spontaneous neuronal noise. It is therefore important to know whether the sequential retrieval of “Self-generation” would be affected in the presence of such spontaneous neuronal noise. In this study, we showed that the activities of cell assemblies for self-generation were robust to spontaneous neuronal noise when the noise level was < 1/6 of the external stimulus. However, this study did not provide comprehensive simulations of the intensities of various external stimuli and the number of stimulated neurons. This limits our understanding of how robust cell assemblies are to noise. Future studies should be conducted to understand the effects of spontaneous neuronal noise on the self-generation of sequential memory through the loop structure of cell assemblies. Moreover, 1/6 of the external stimulus can be considered as a low level of spontaneous neural noise. The simulation results show that “self-generation” can fail even under such small noises (Figure 7). This can be seen as a limitation of this study. Therefore, future studies are needed, such as additional theoretical predictions based on cell assembly models that can include the presence of spontaneous noise, or additional simulation studies under conditions that can be more robust to spontaneous noise.

Nonetheless, this study elucidated the relationship between the size of brain loop structures and the formation of sequential memories. By revealing that a moderate-sized structure is most suitable for the formation of sequential memory, it provided insight into why the biological brain loop structure is moderate-sized. The results of this study may help to advance our understanding of the relationship between brain structures and functions involved in sequential memory.

## Data availability statement

The original contributions presented in the study are included in the article/Supplementary material, further inquiries can be directed to the corresponding author.

## Author contributions

DS: Conceptualization, Data curation, Formal analysis, Funding acquisition, Investigation, Methodology, Project administration, Resources, Software, Supervision, Validation, Visualization, Writing – original draft, Writing – review & editing. S-PK: Funding acquisition, Project administration, Supervision, Visualization, Writing – original draft, Writing – review & editing.
