# Supplementary material for: A neural basis for learning sequential memory in brain loop structures
Source: Front Comput Neurosci. 2024 Aug 5;18:1421458. doi: 10.3389/fncom.2024.1421458 (PMC11330804; doi:10.3389/fncom.2024.1421458)
Supplement: Supplementary file 1 [file Data_Sheet_1.docx]

Supplementary Material (A neural basis for learning sequential memory in brain loop structures)

# Disclosure of the use of generative AI technology

During the preparation of the manuscript, the authors used Google Translate and ChatGPT 4.0 in order to improve readability and English writings. After using these tools, the authors reviewed and edited the texts as needed and took a full responsibility for the contents. We have provided all input prompts to ChatGPT 4.0 and outputs received from it in the supplementary figure as follows:


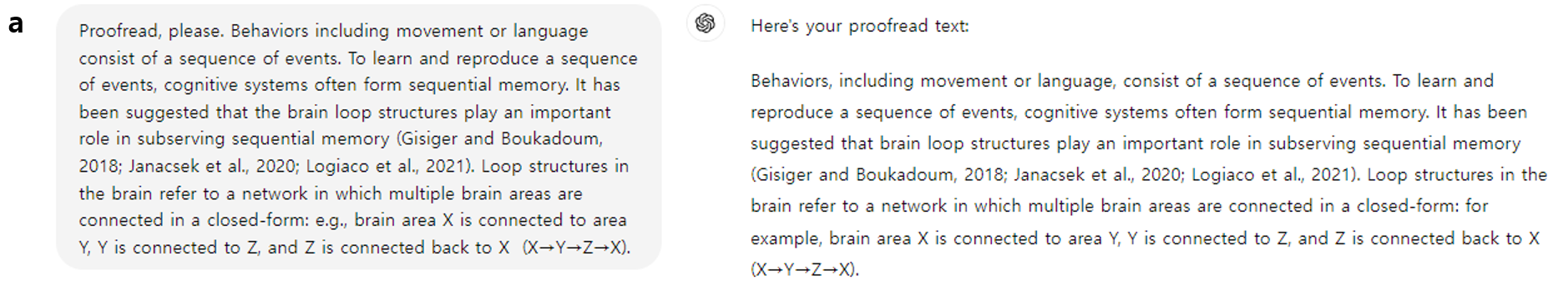


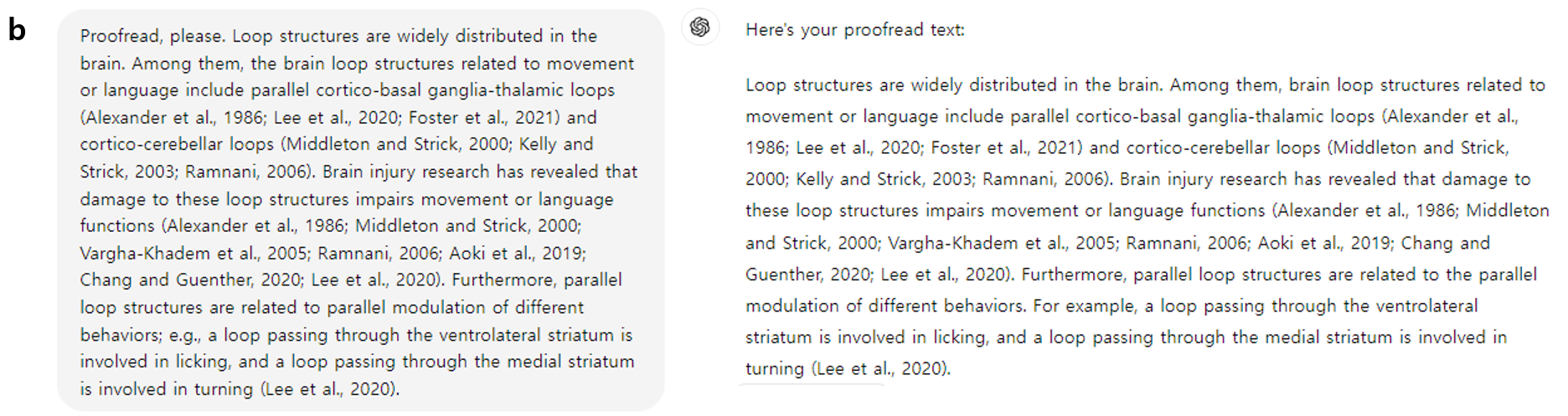


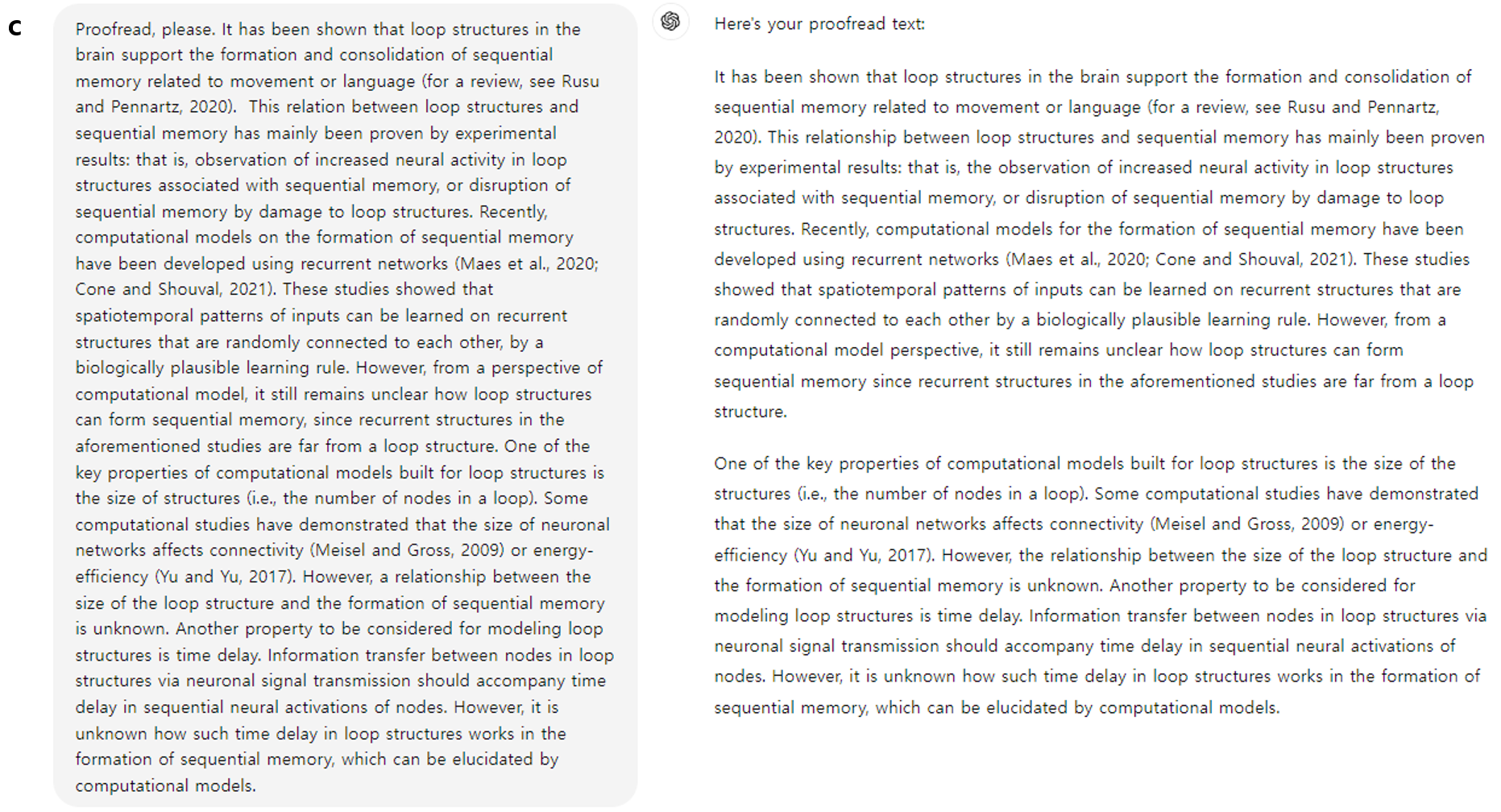


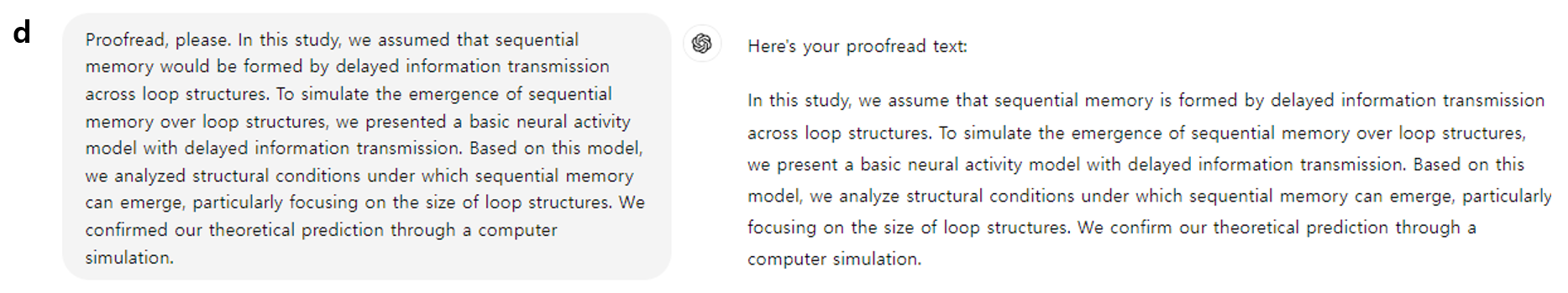


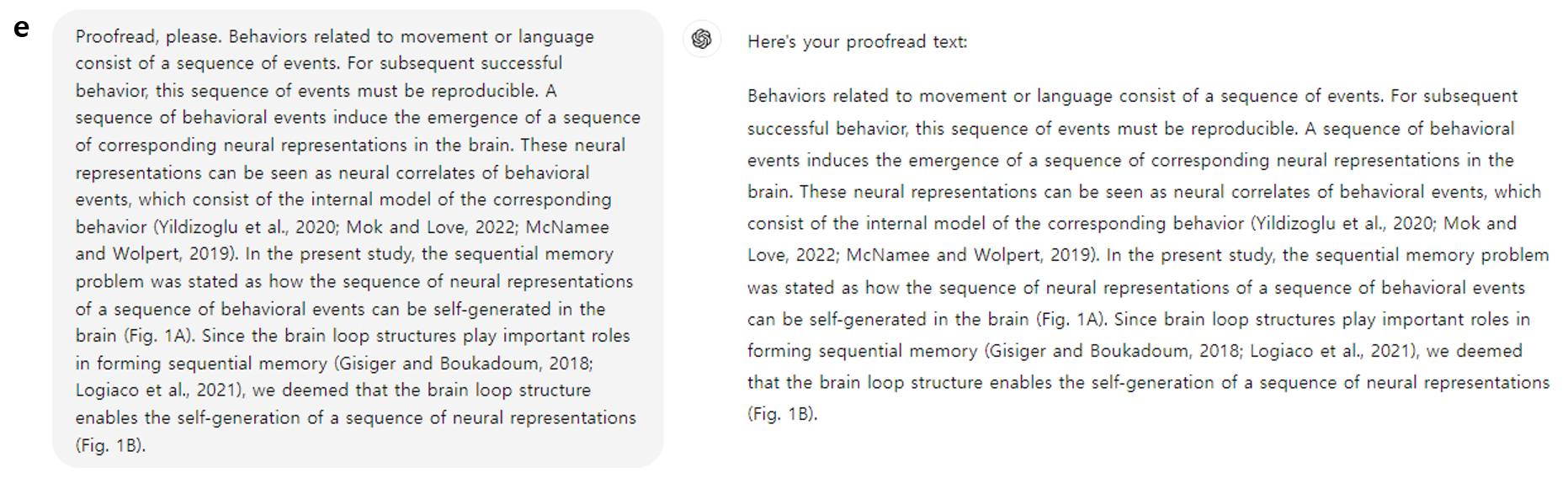


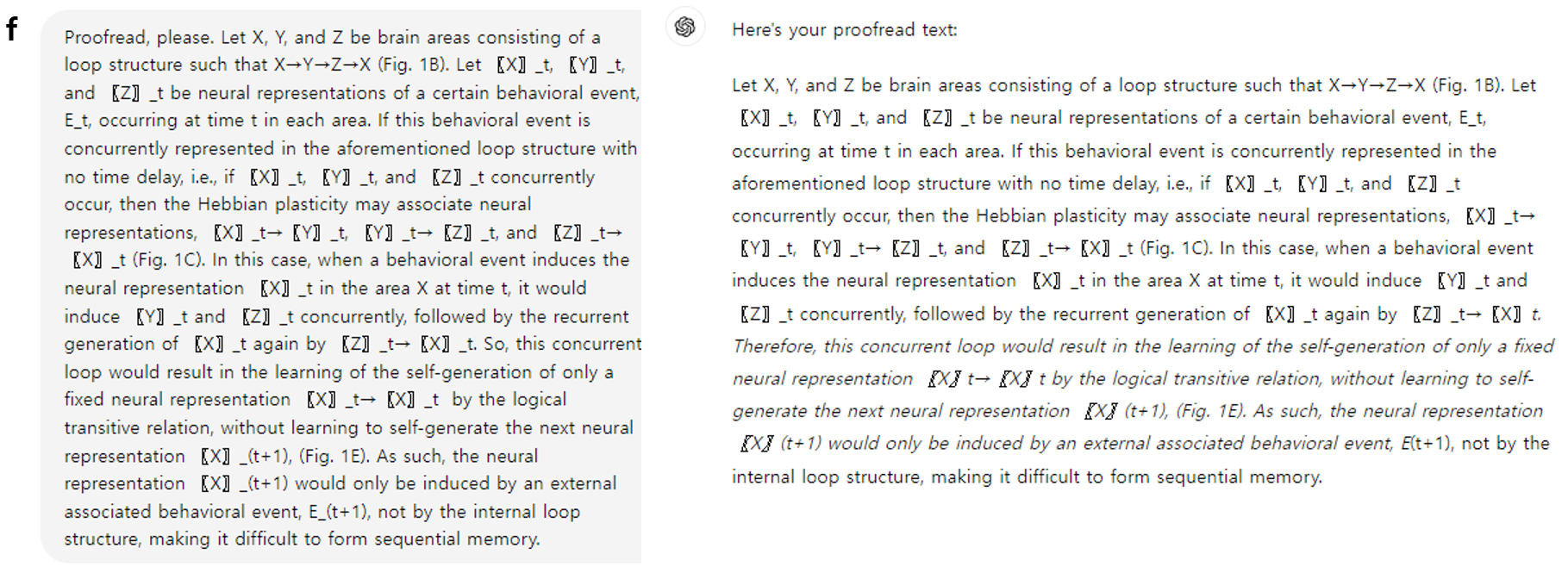


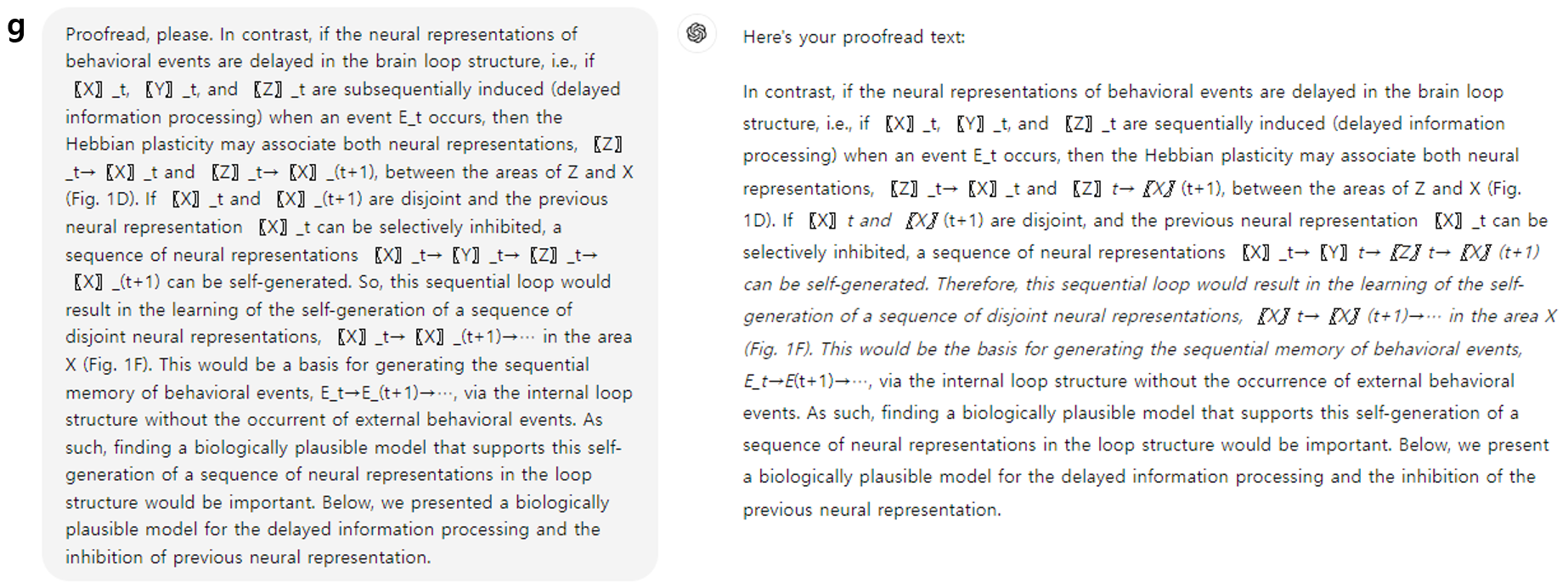


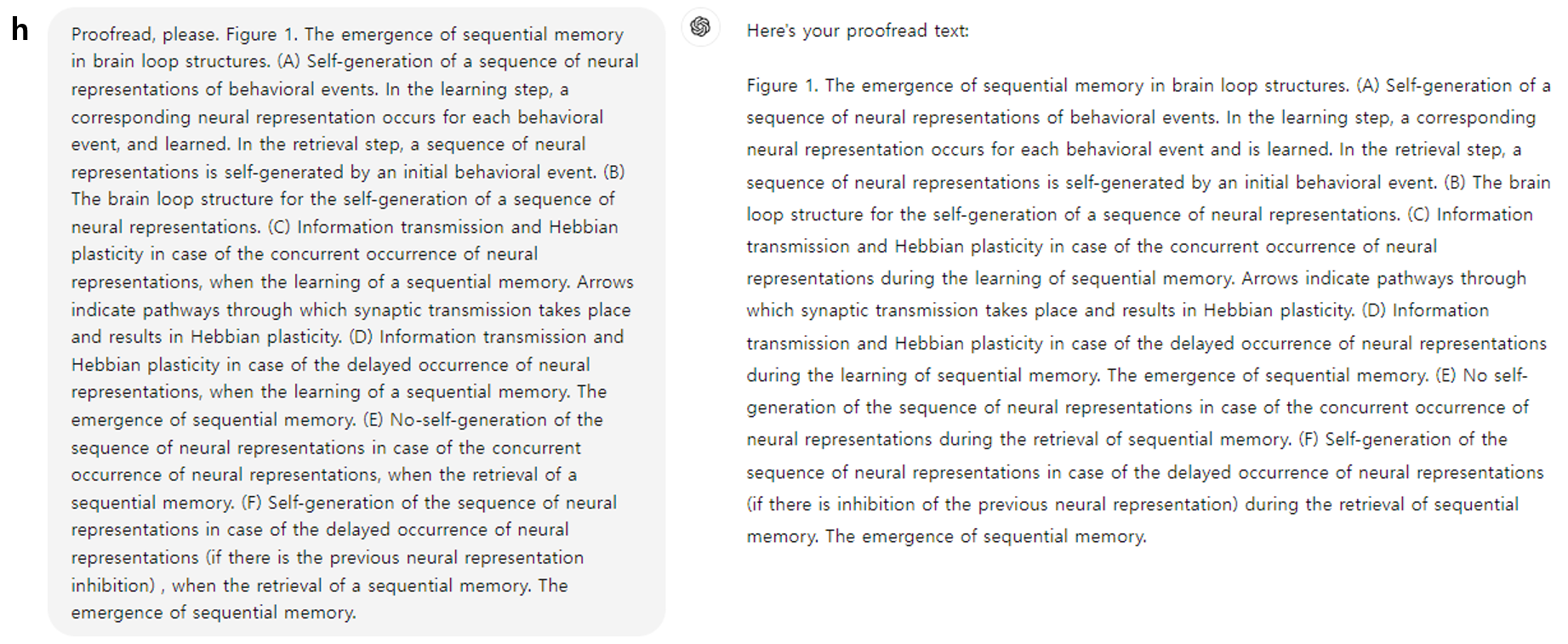


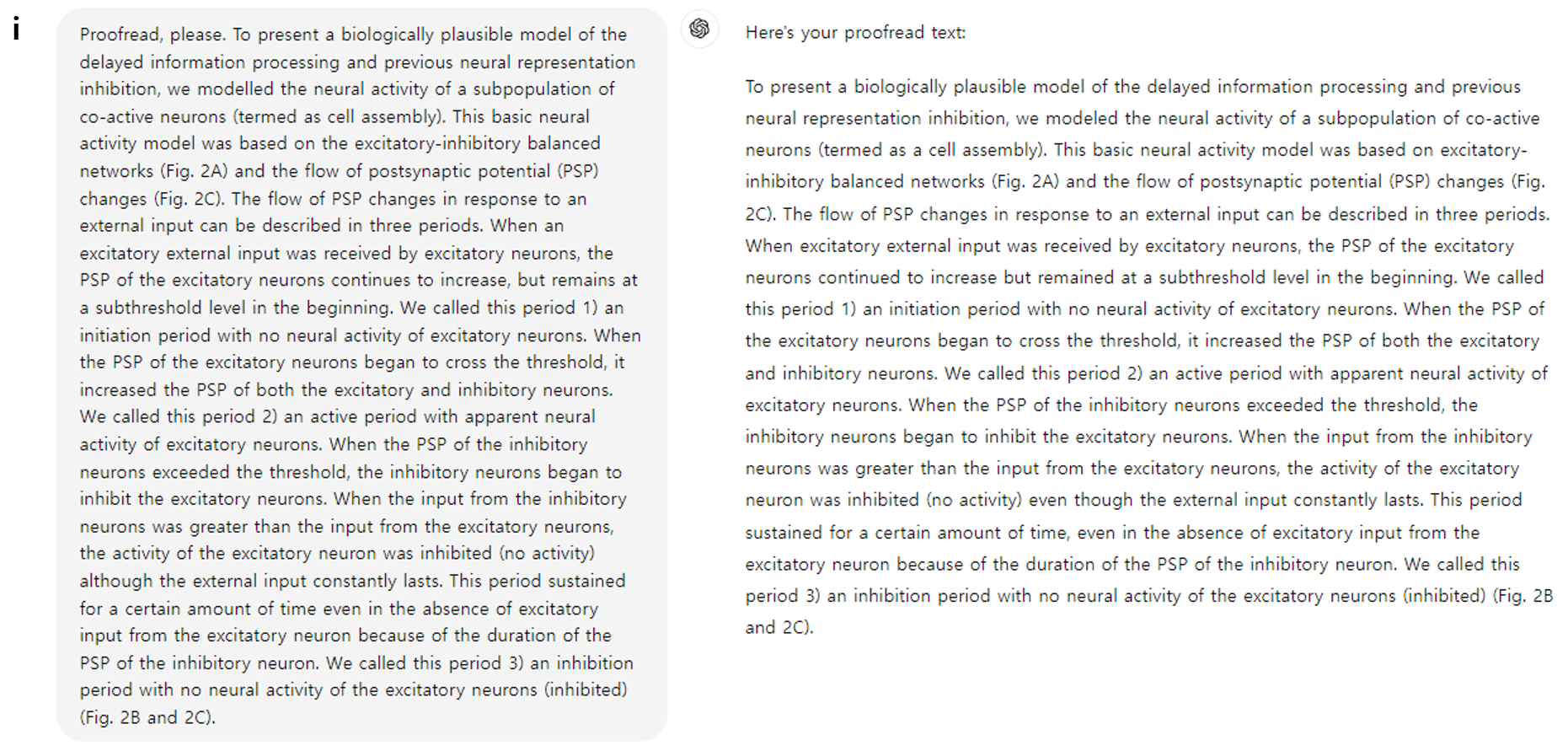


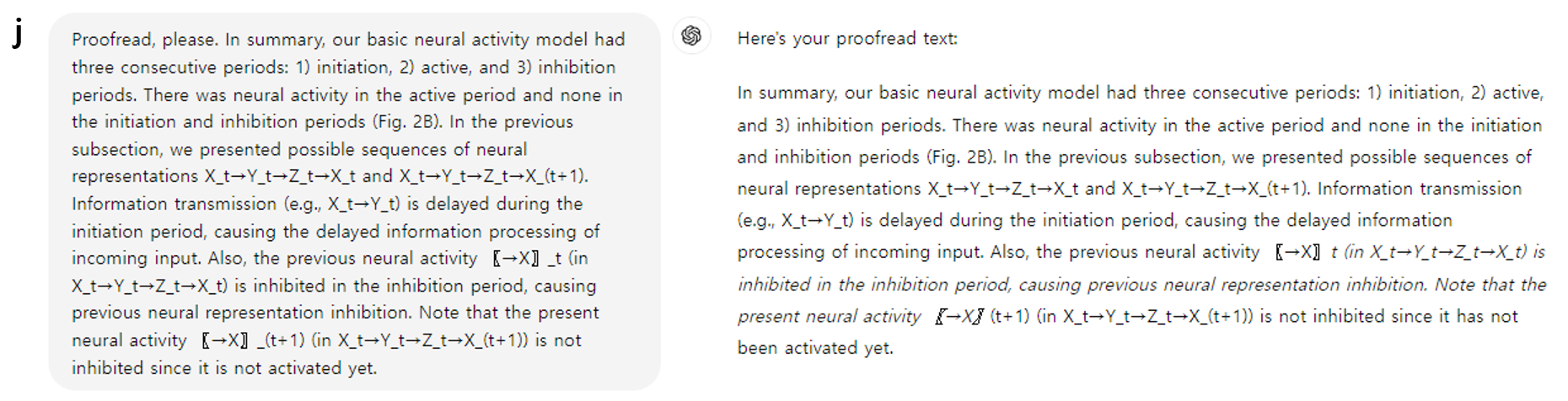


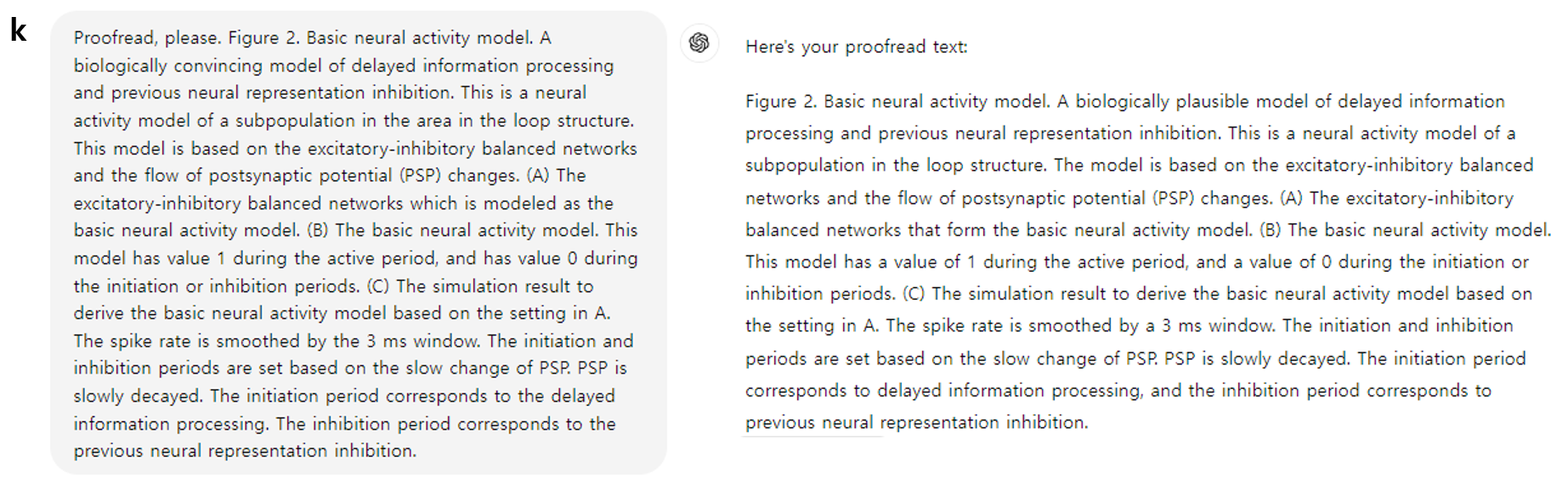


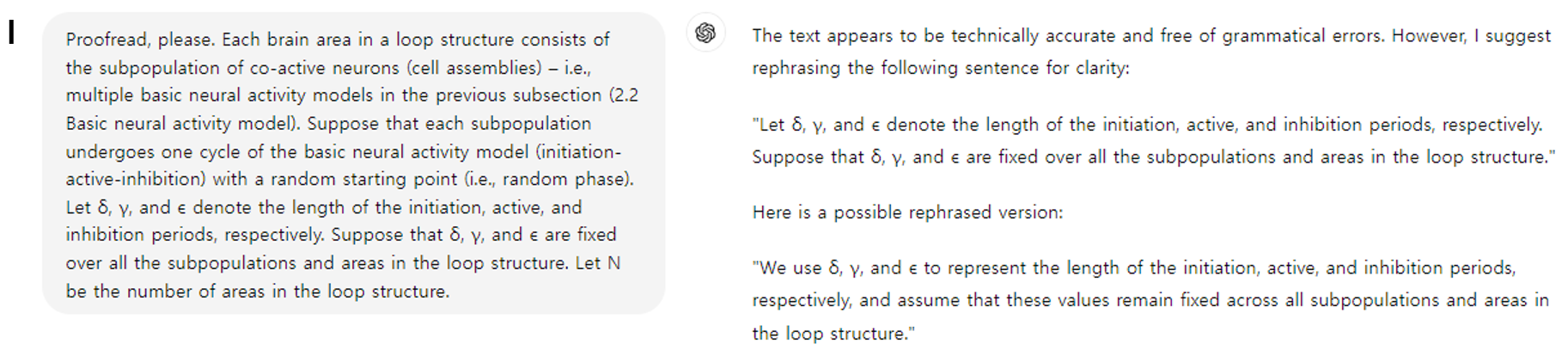


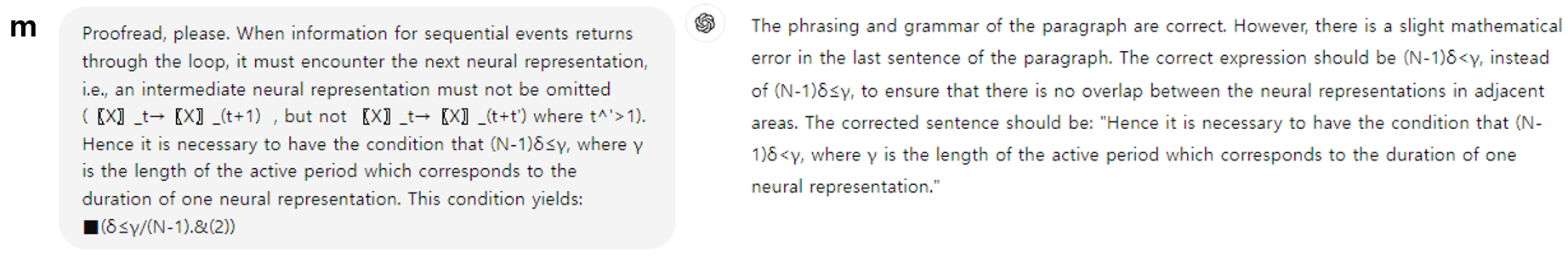


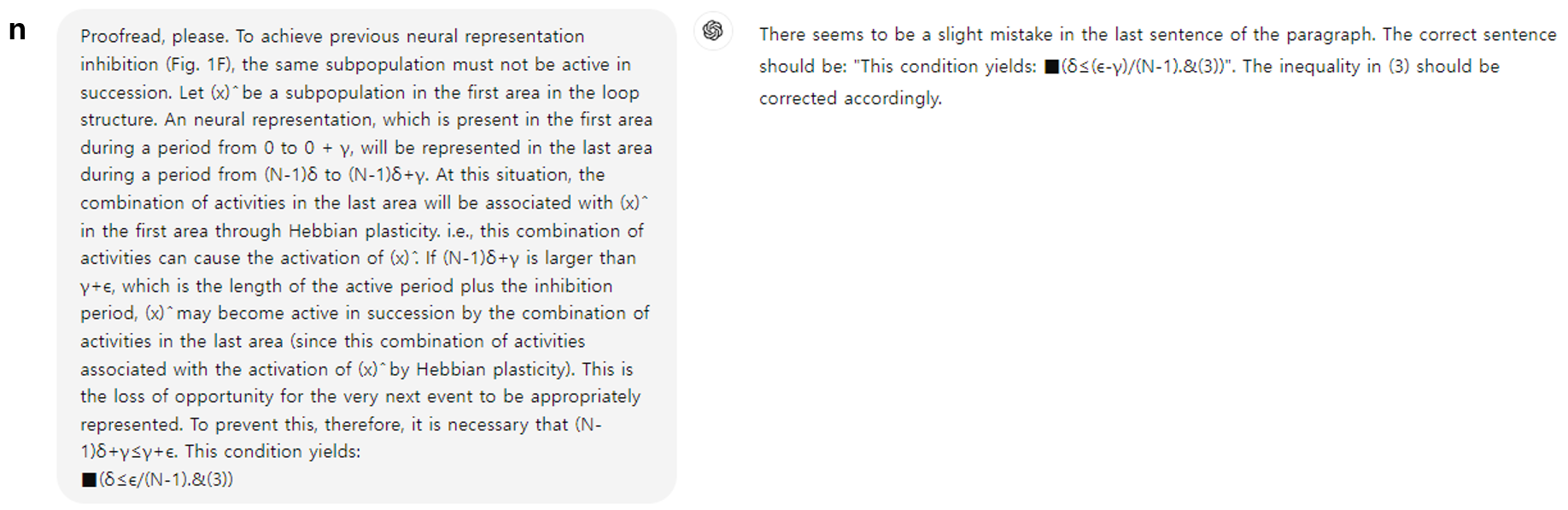


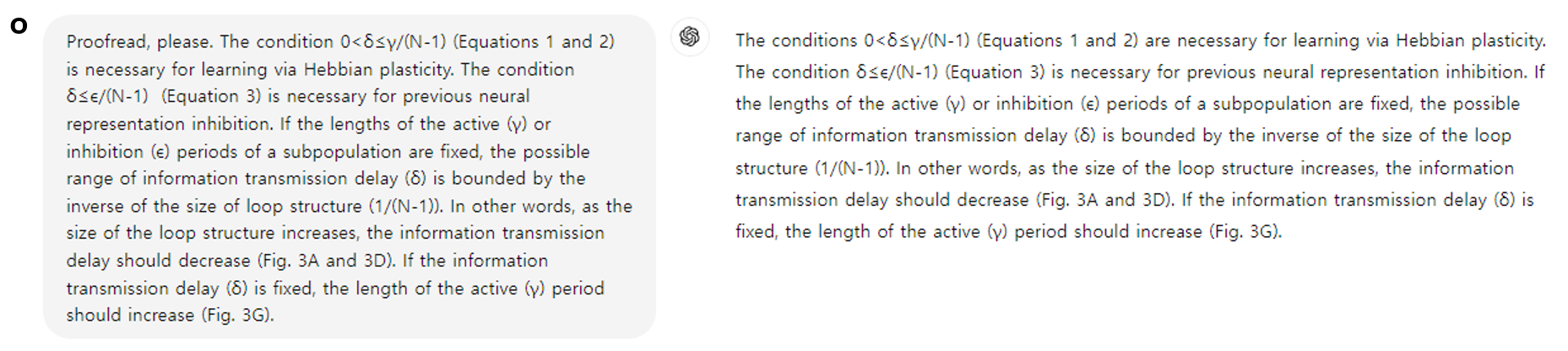


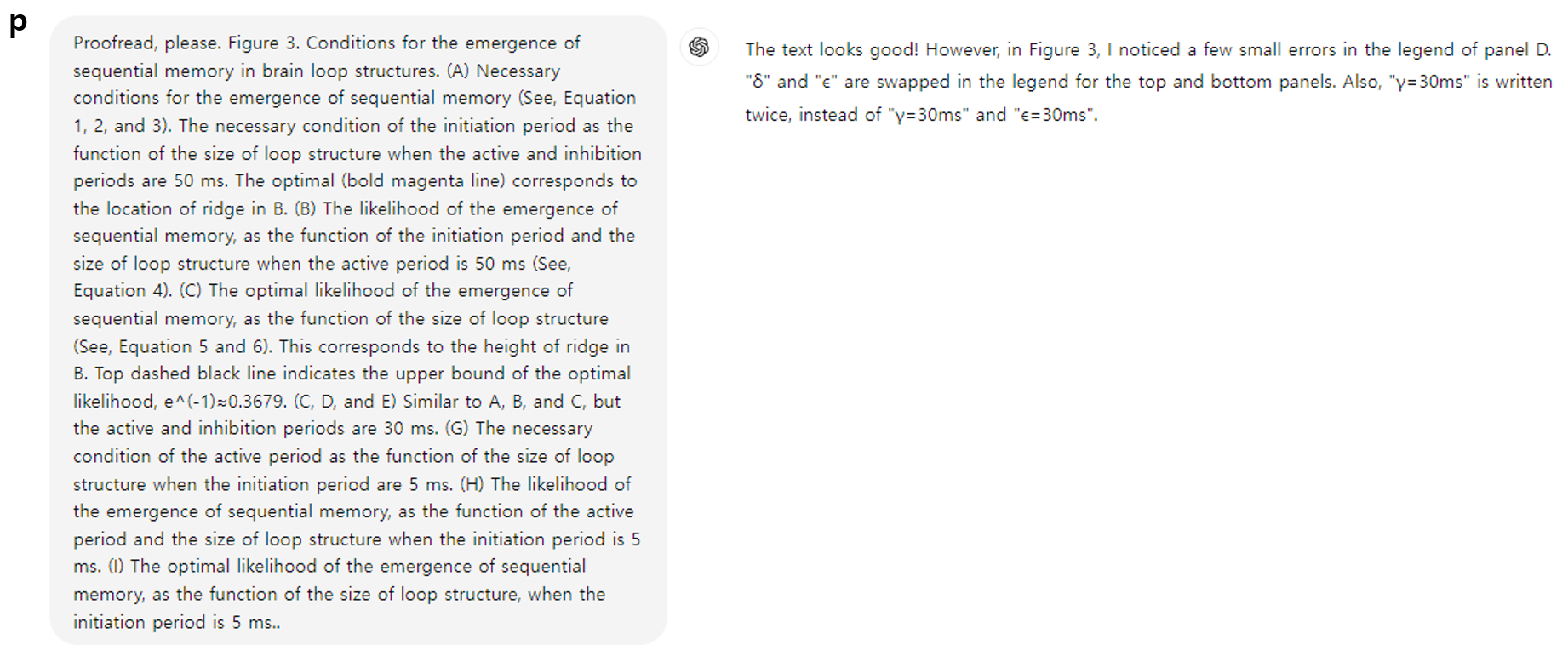


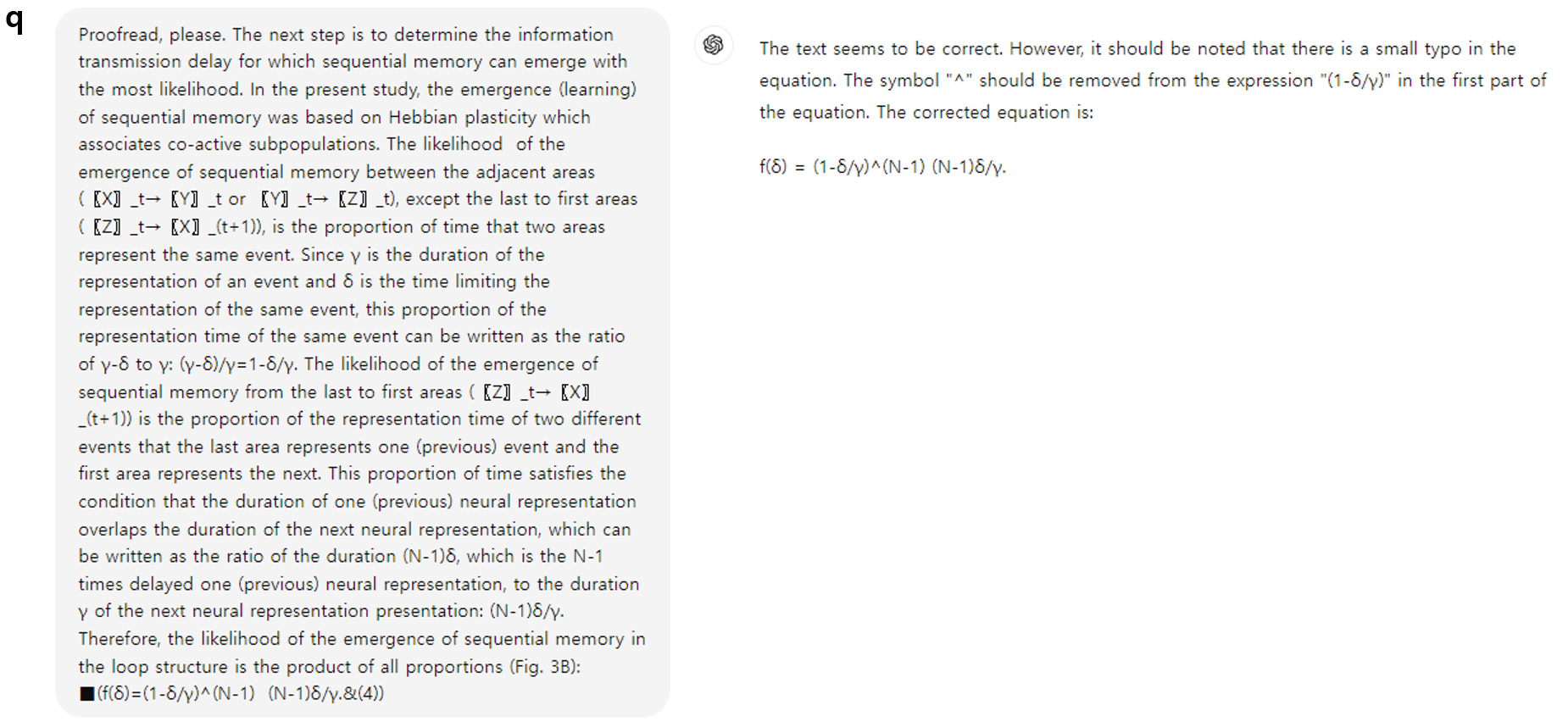


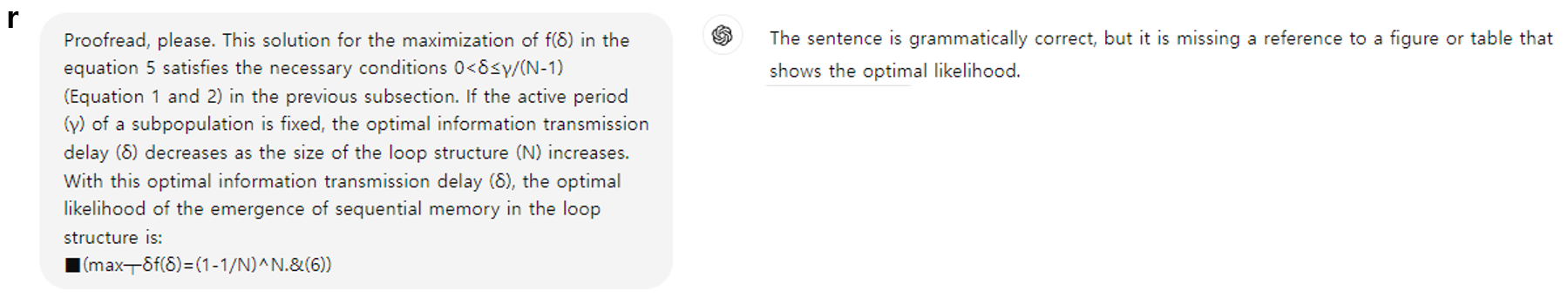


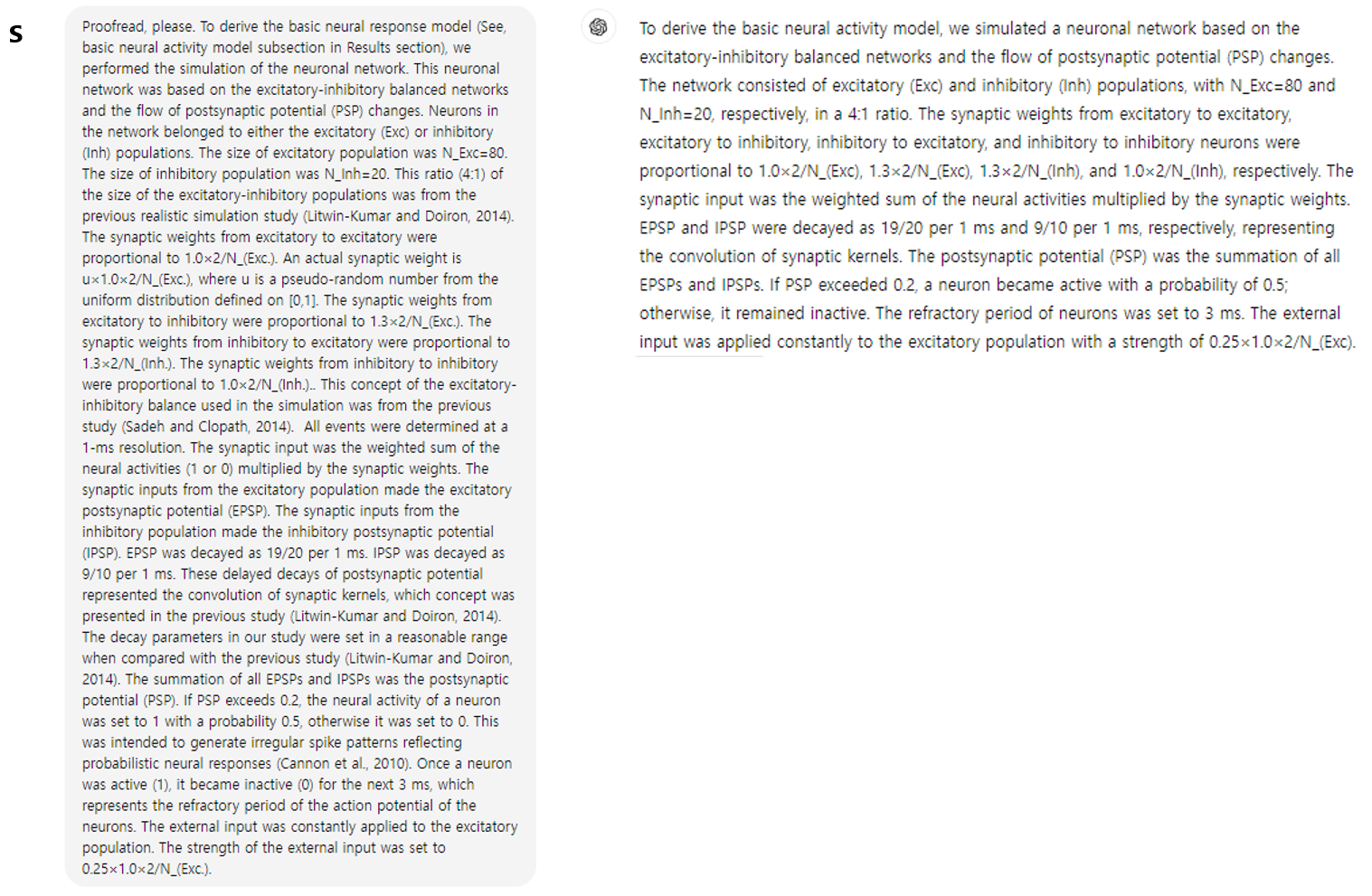


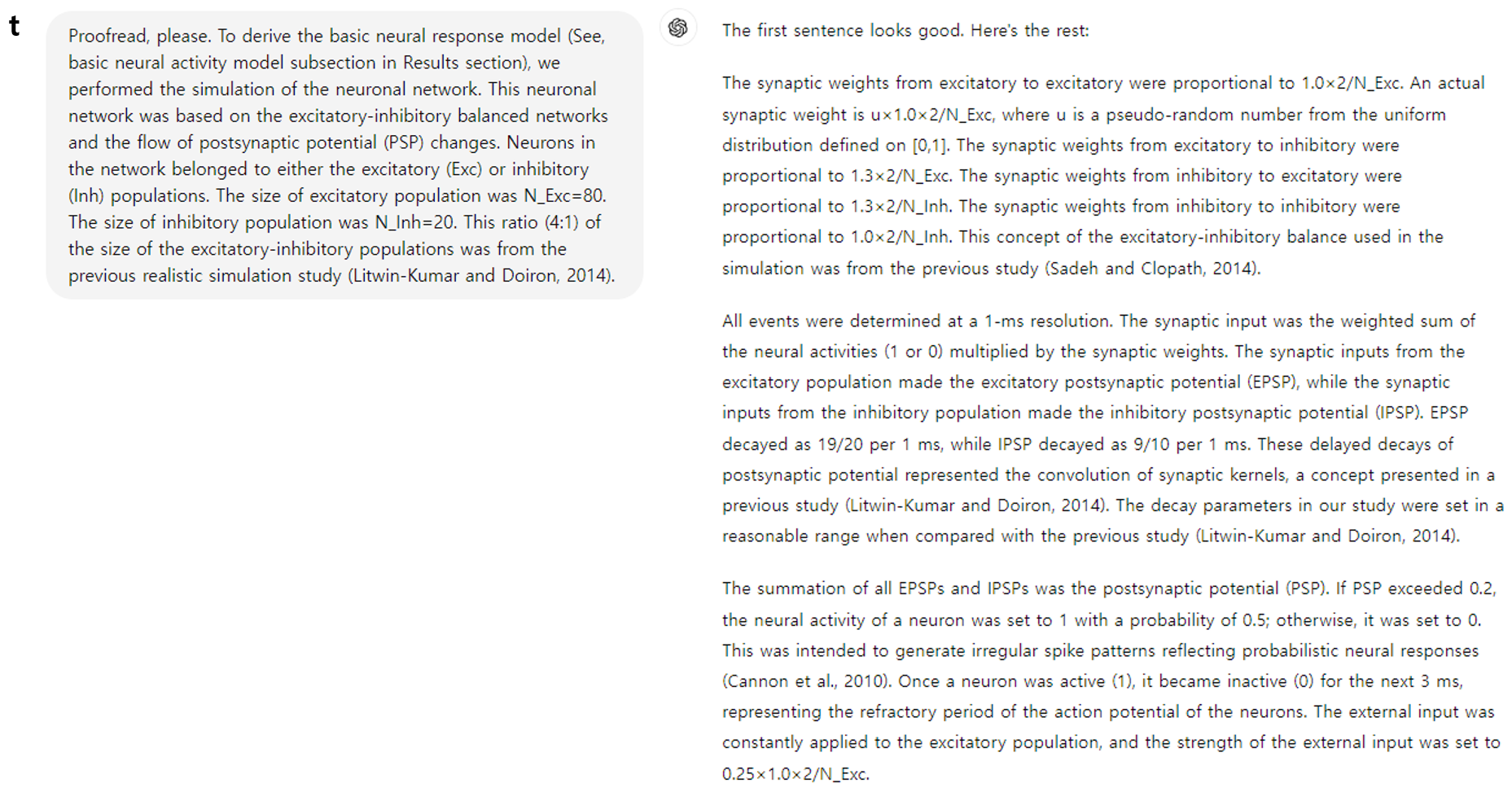


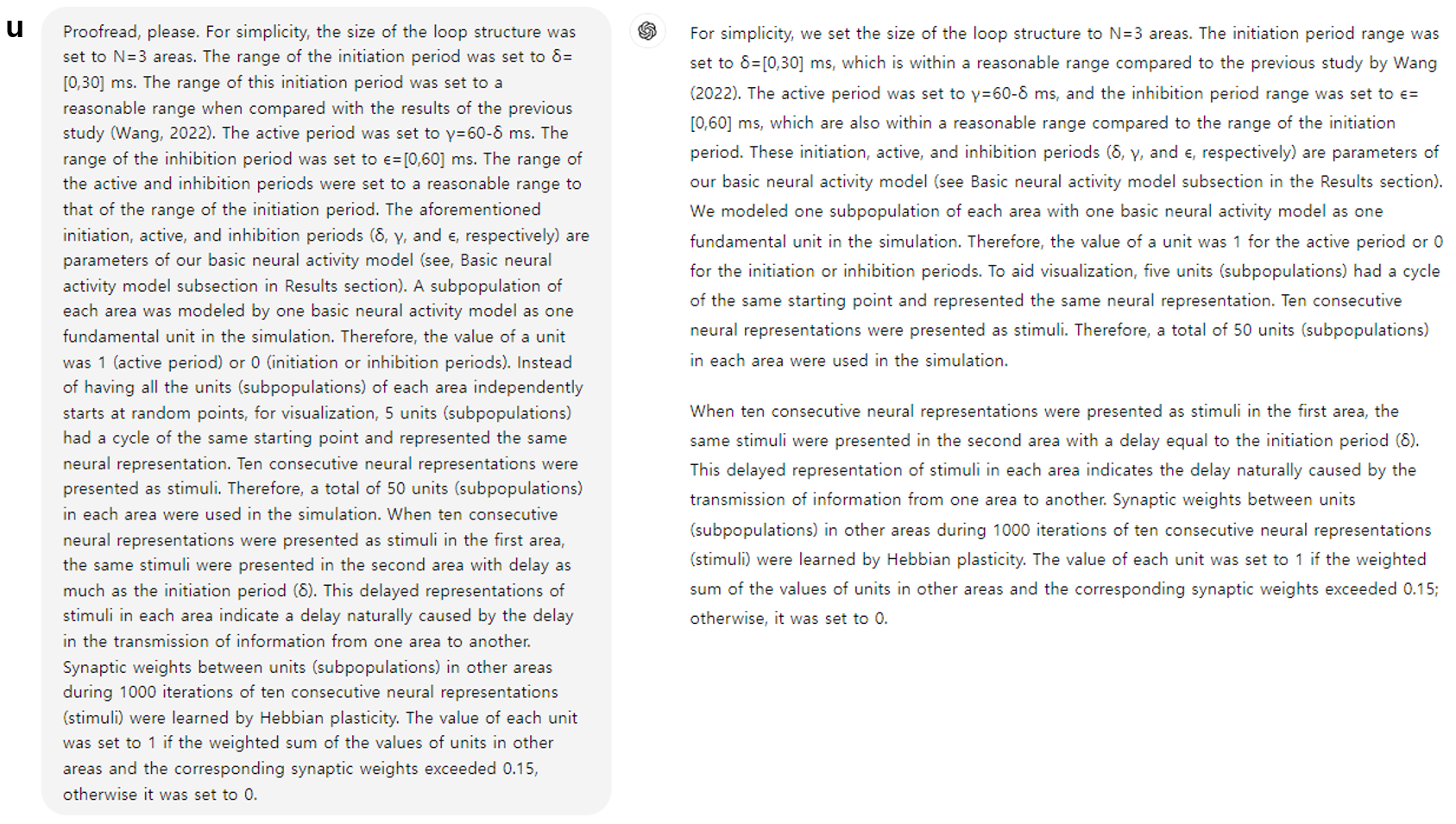


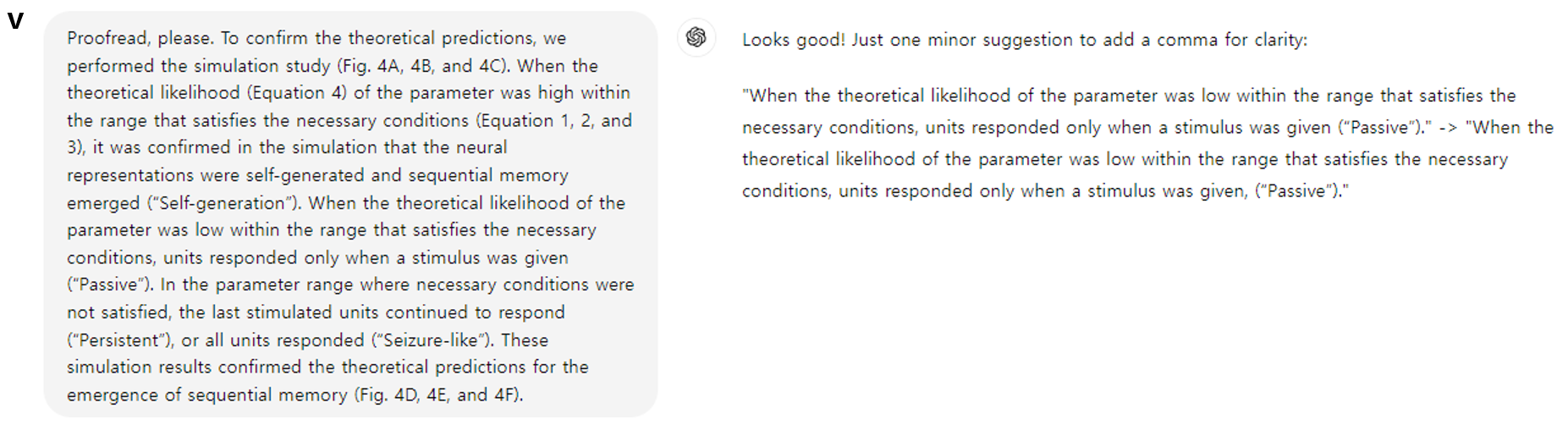


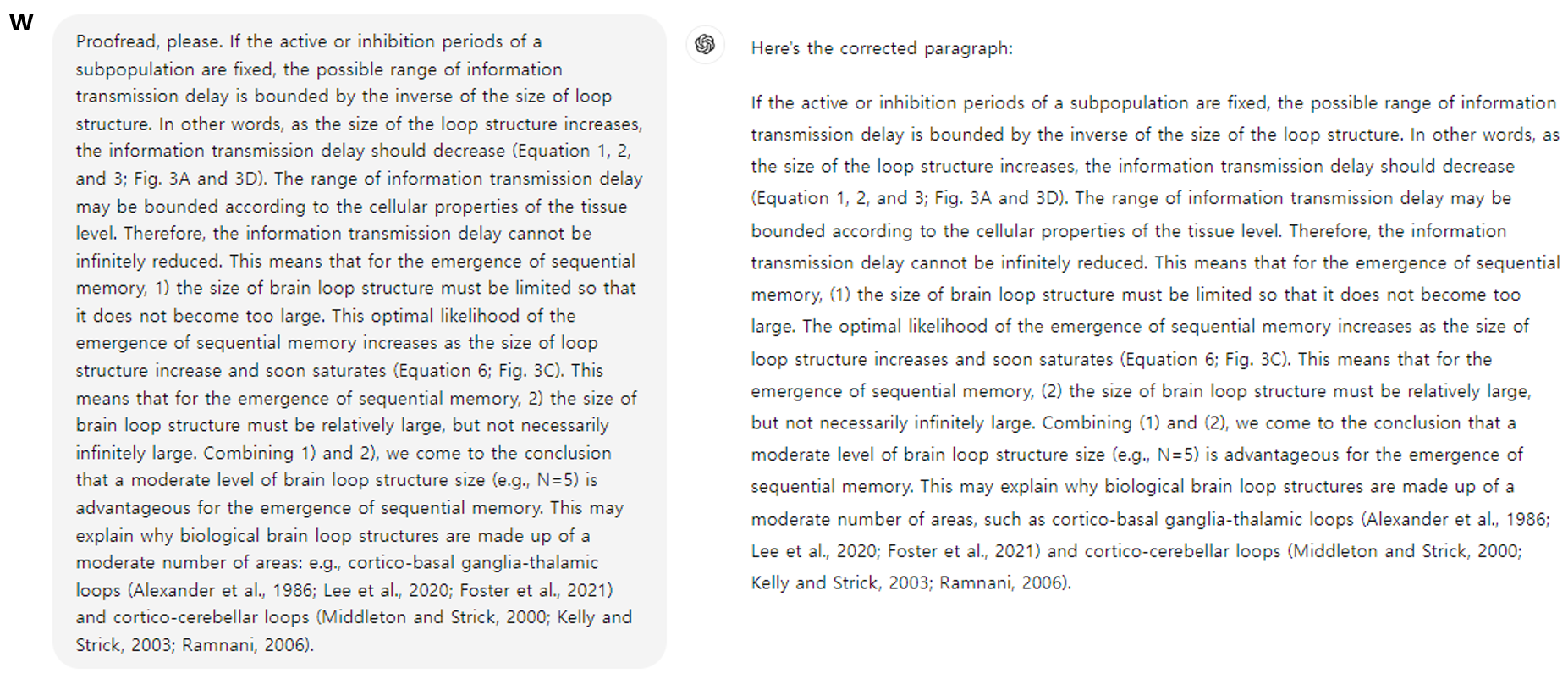


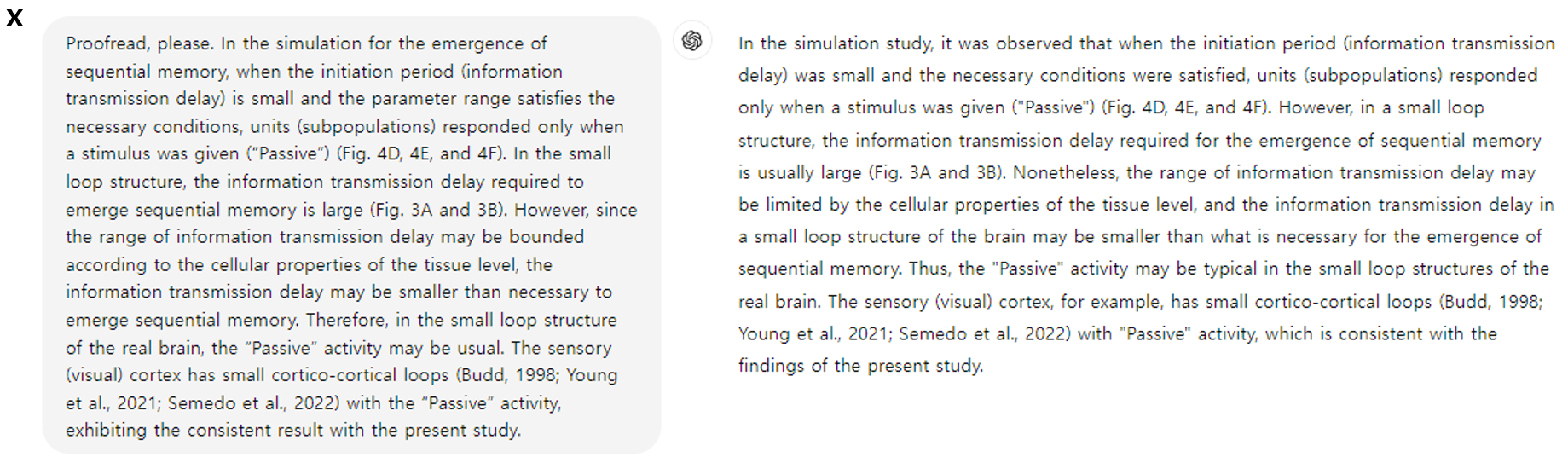


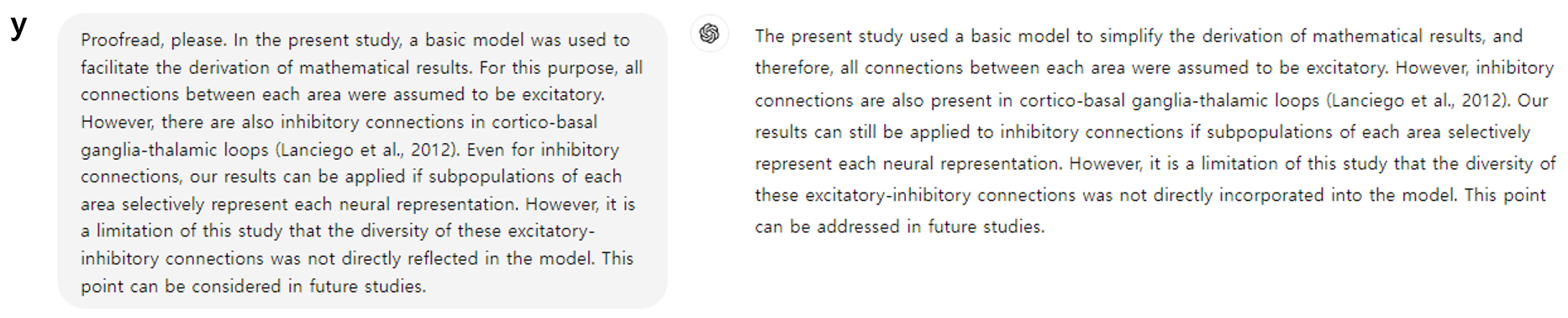


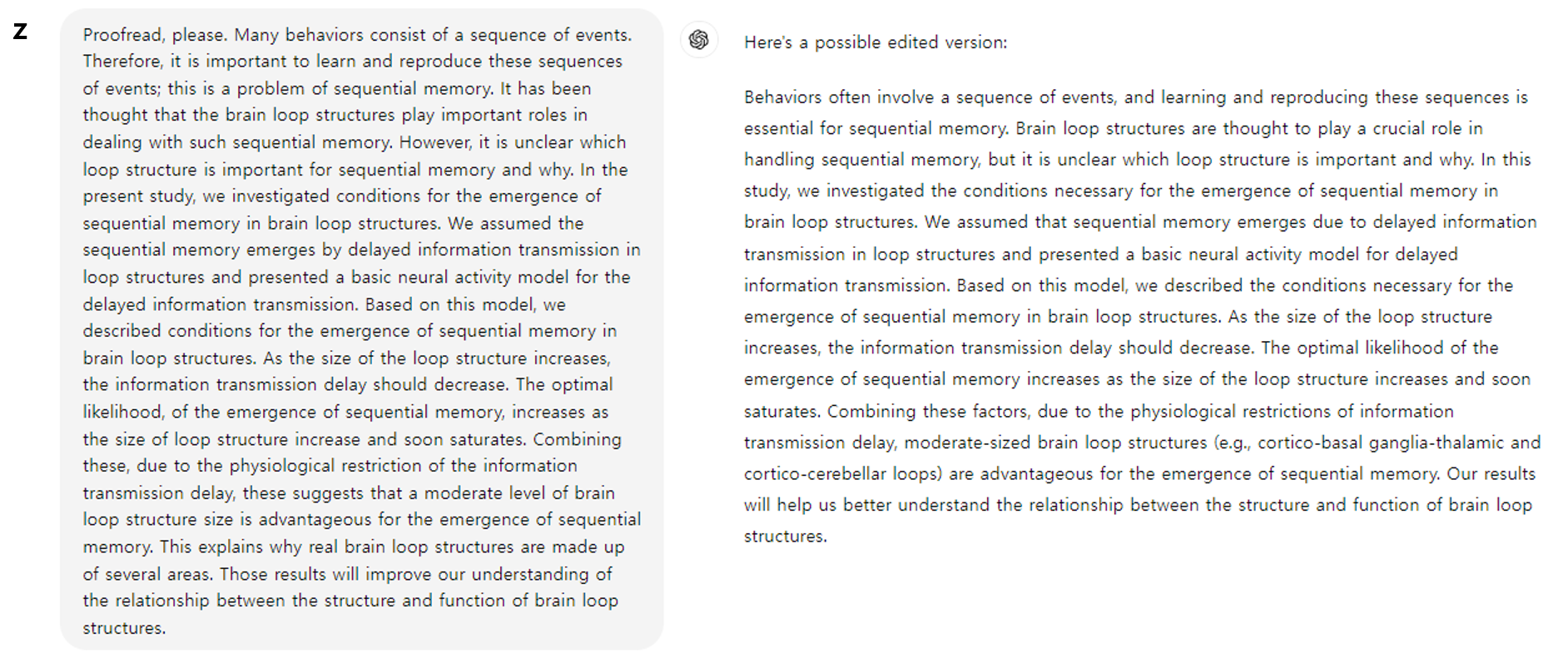


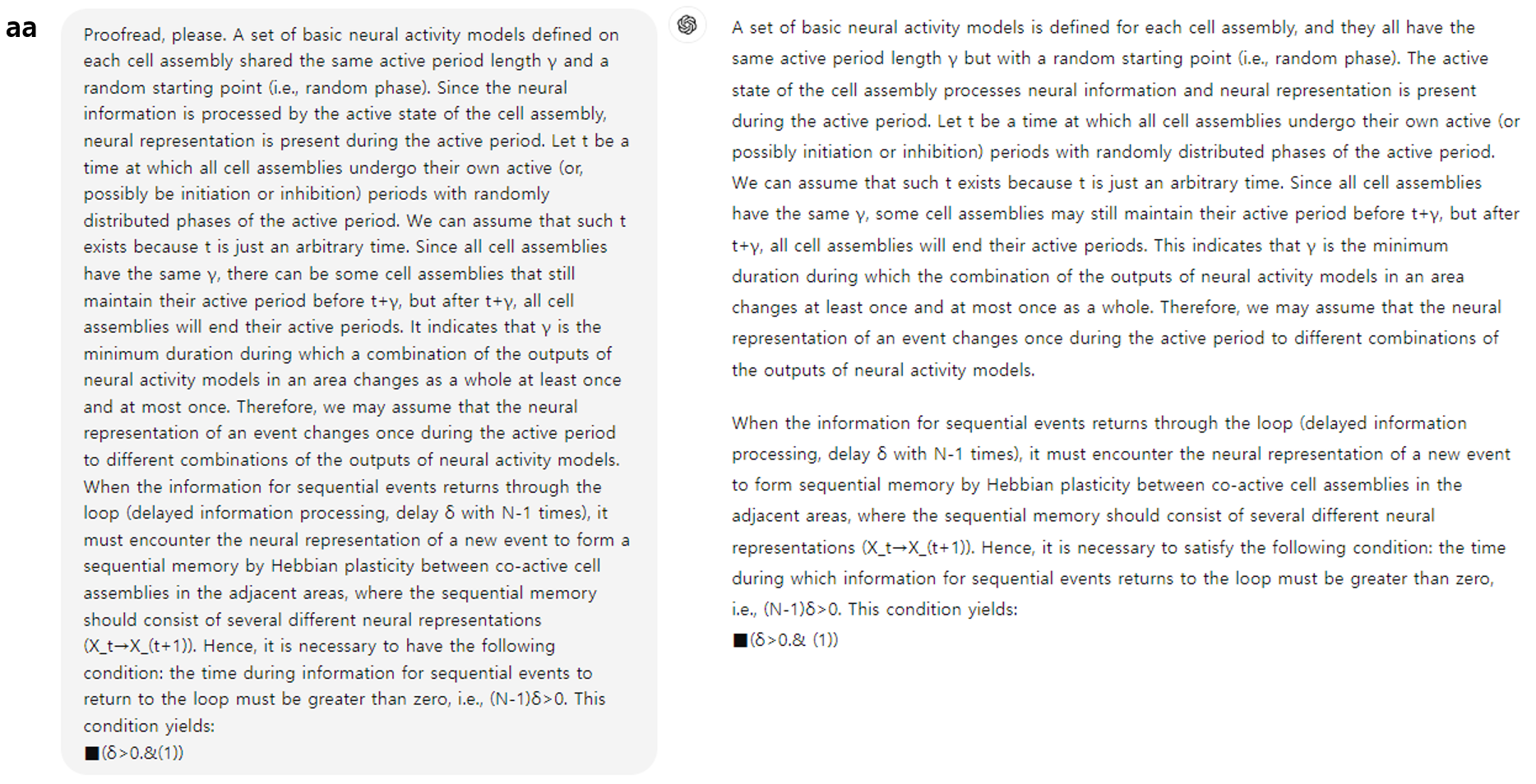


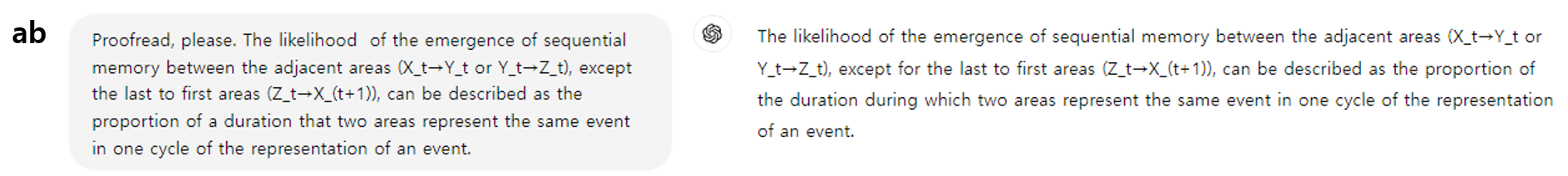


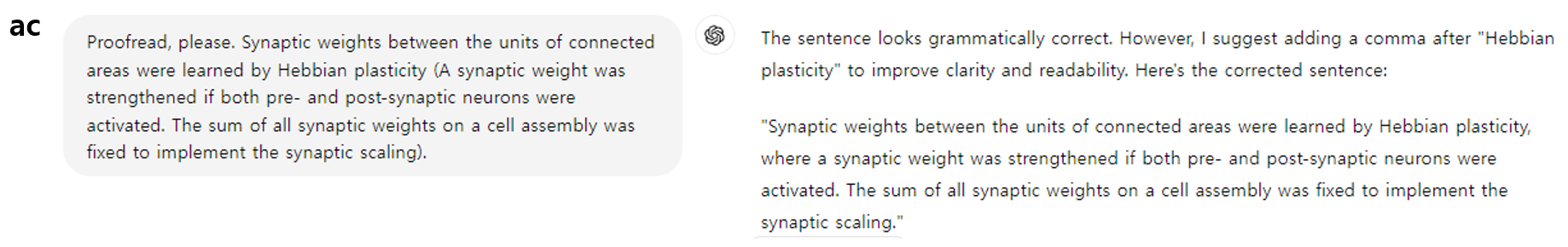


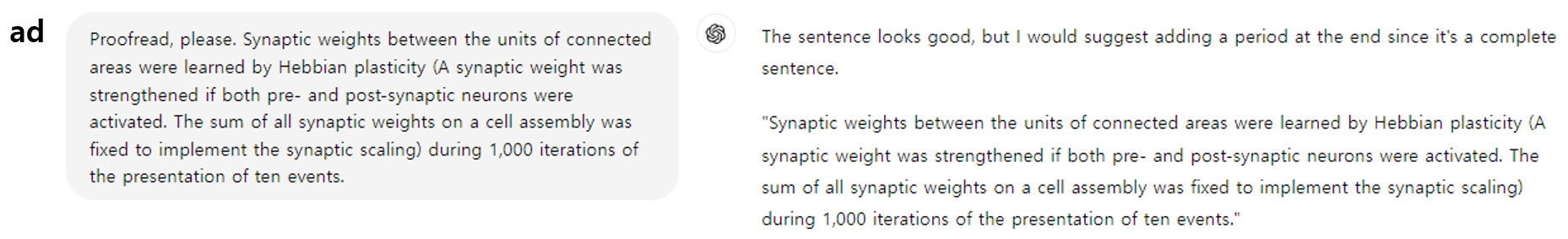


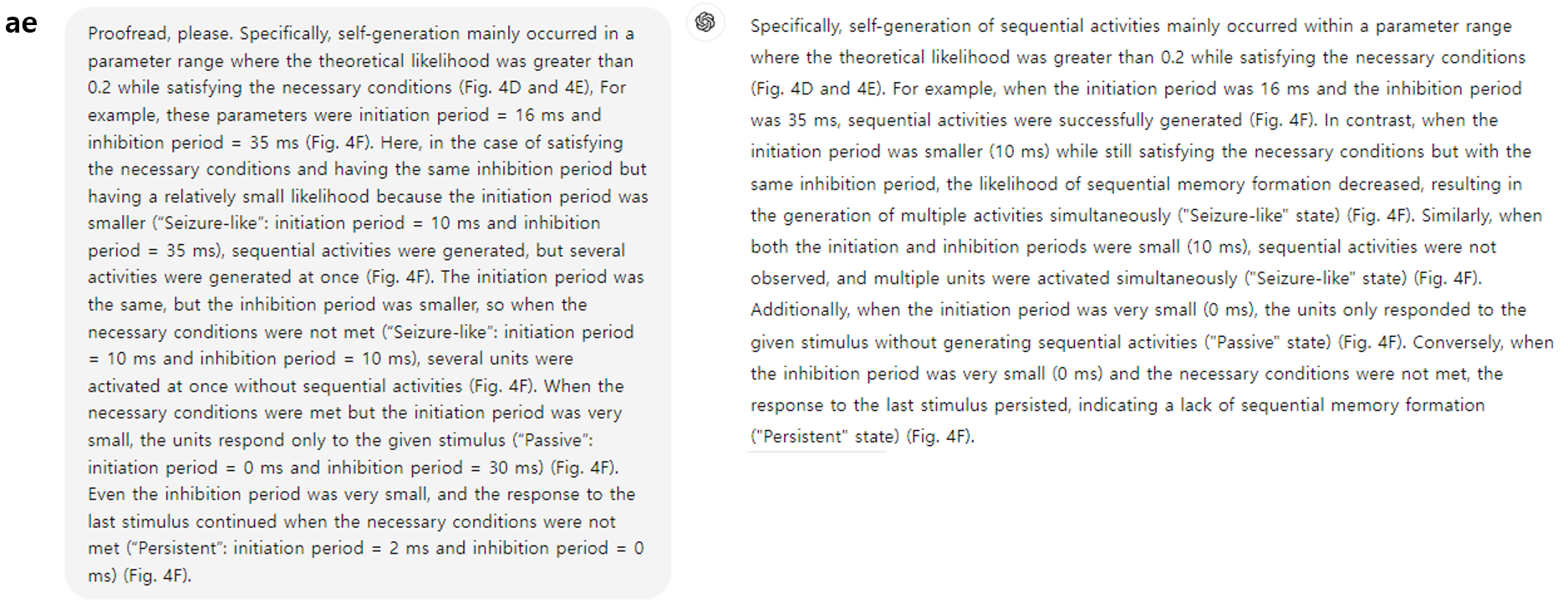


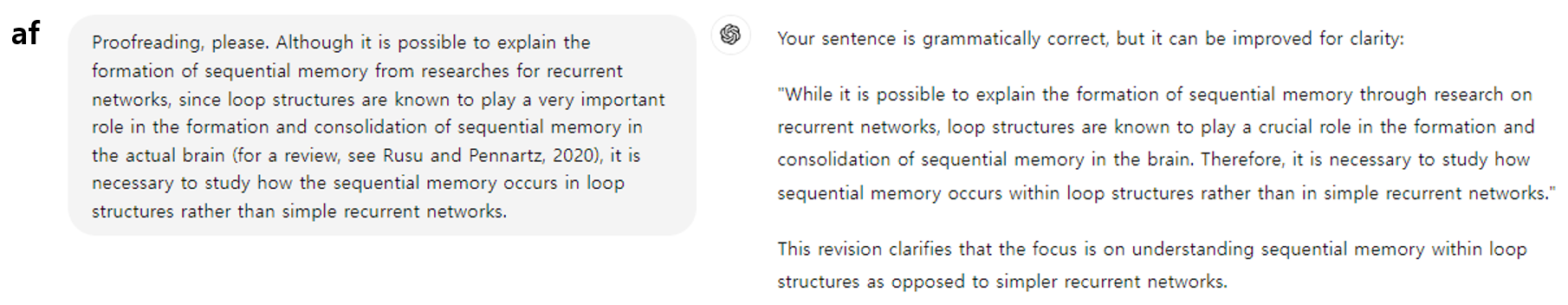


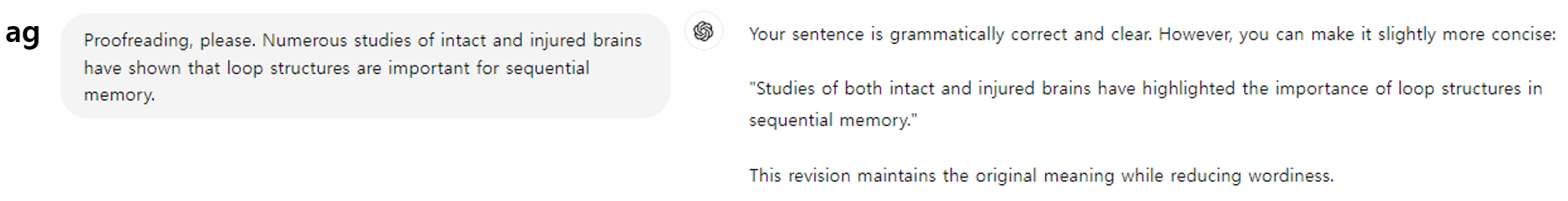


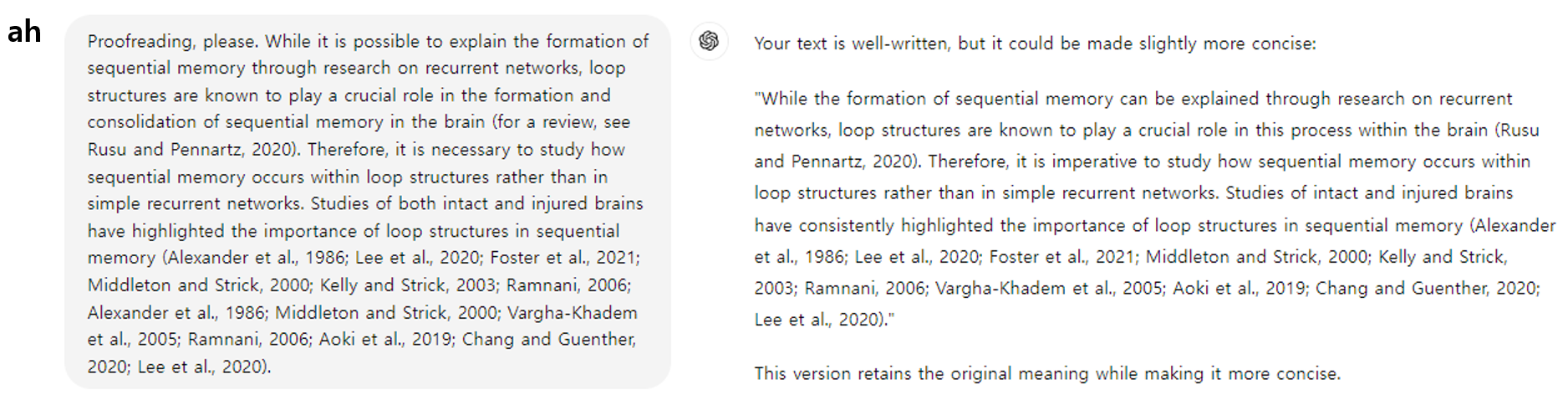


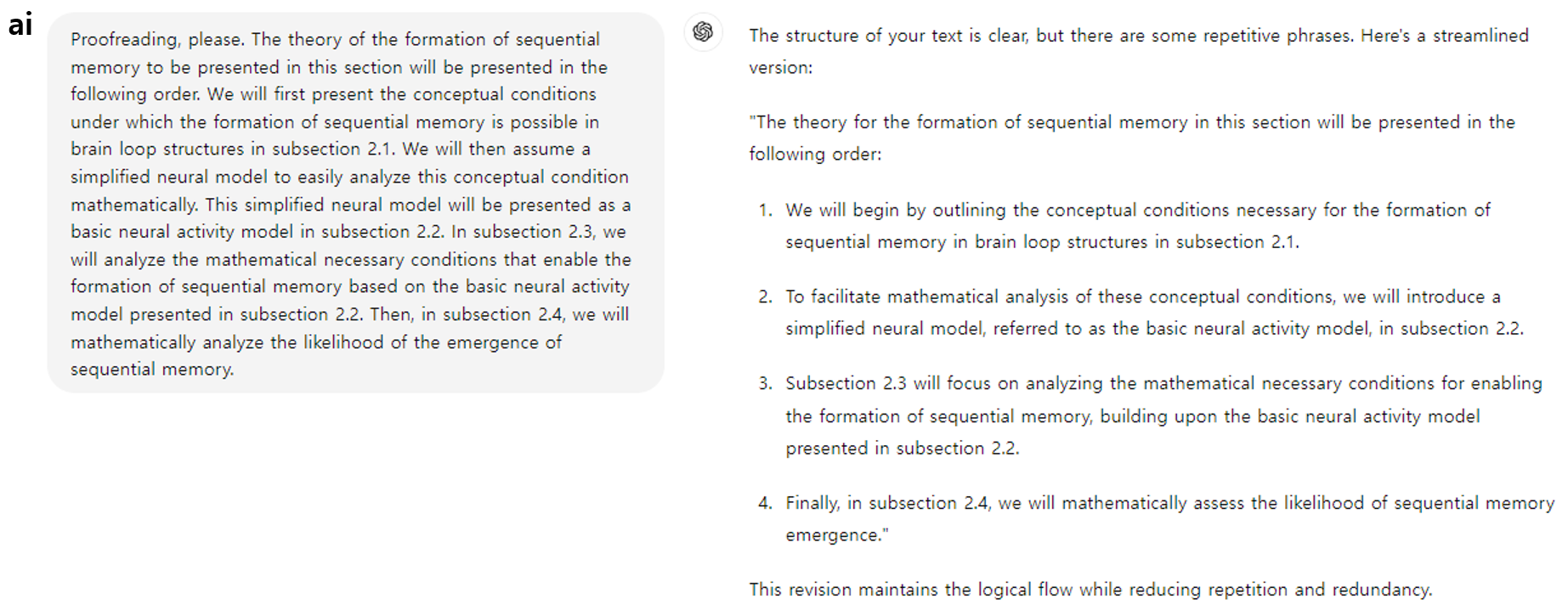


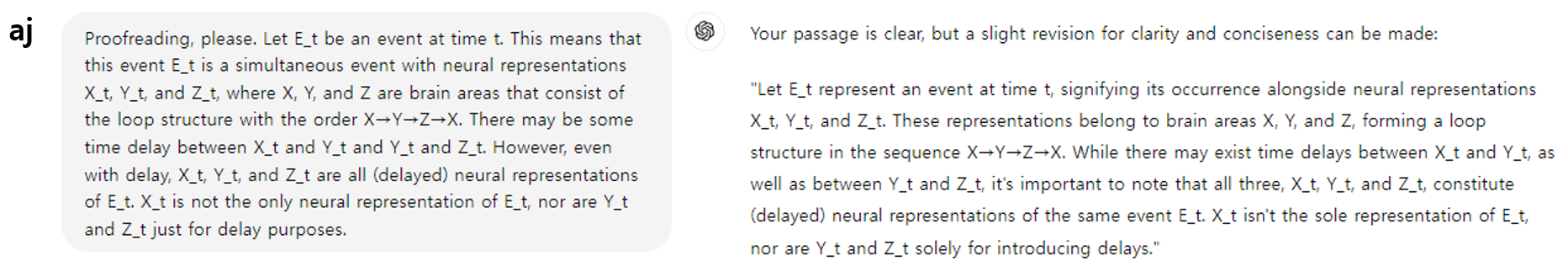


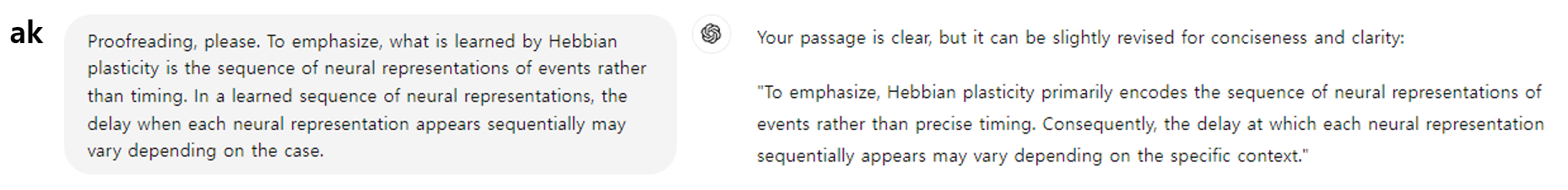


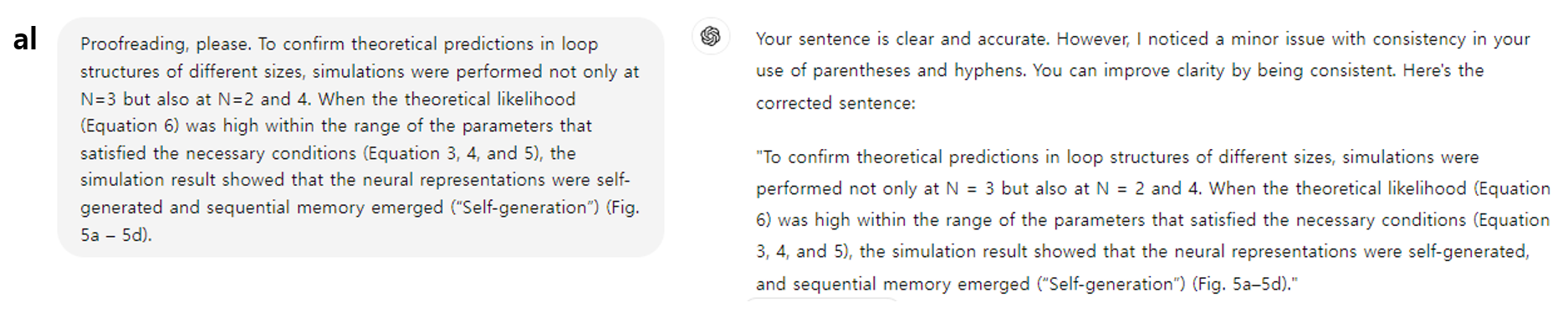


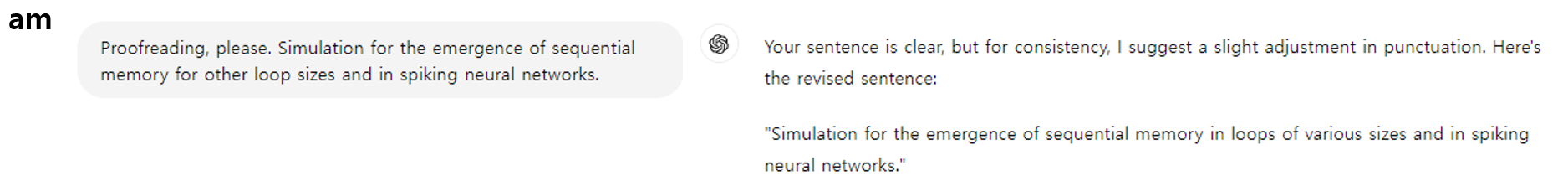


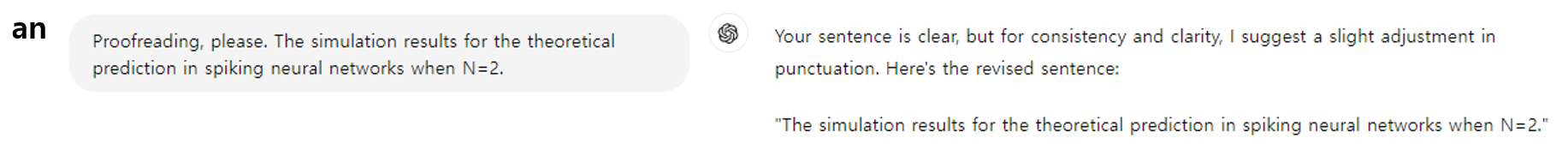


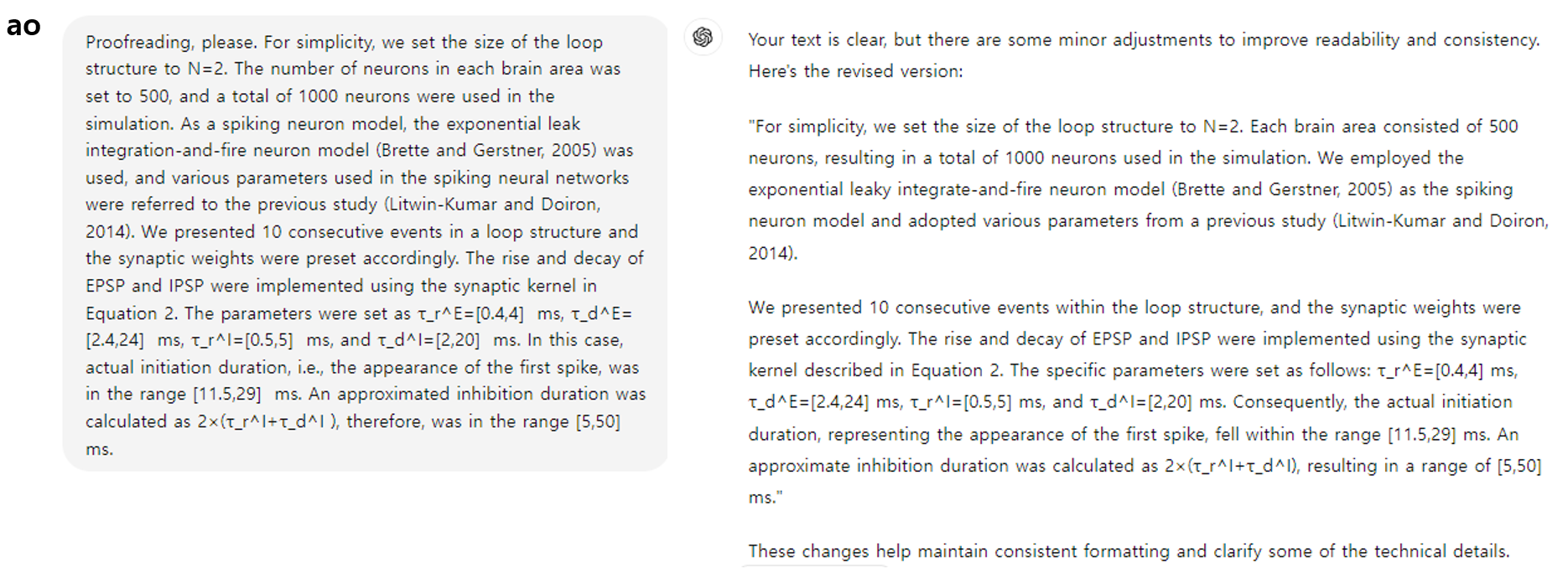


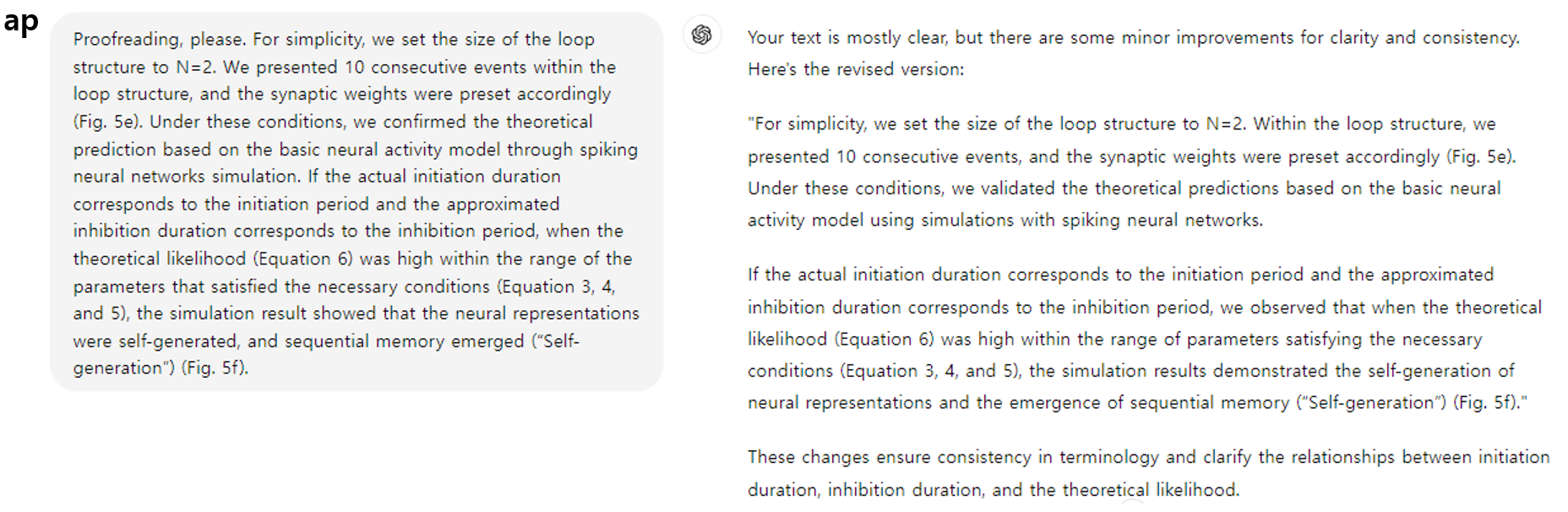


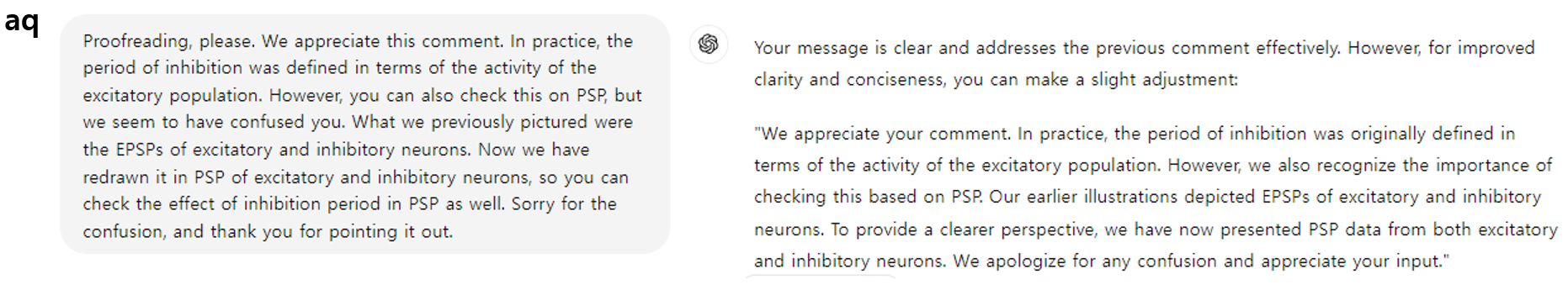


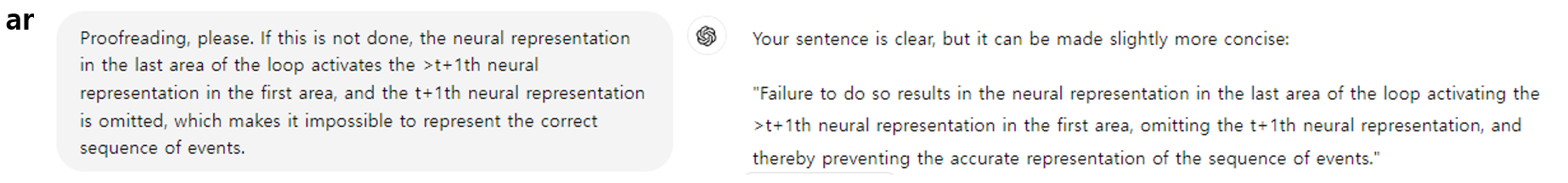


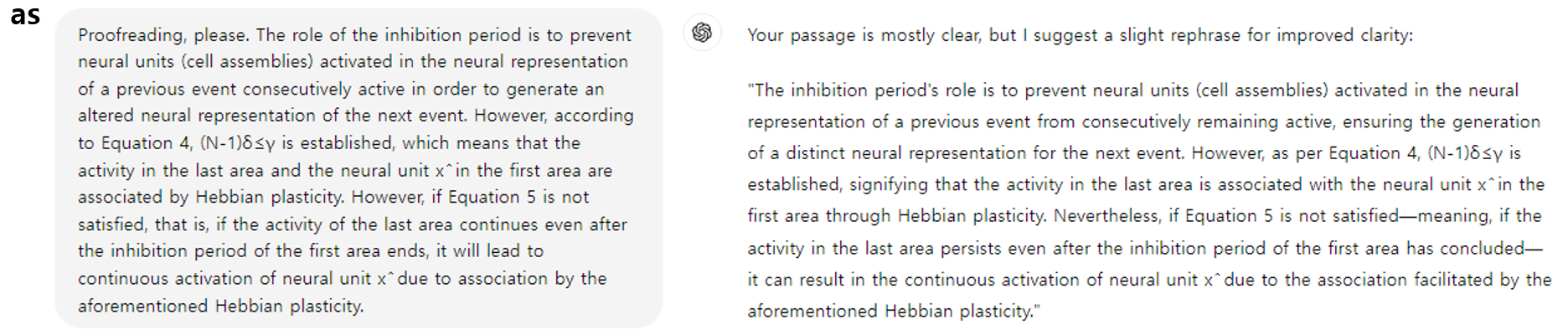


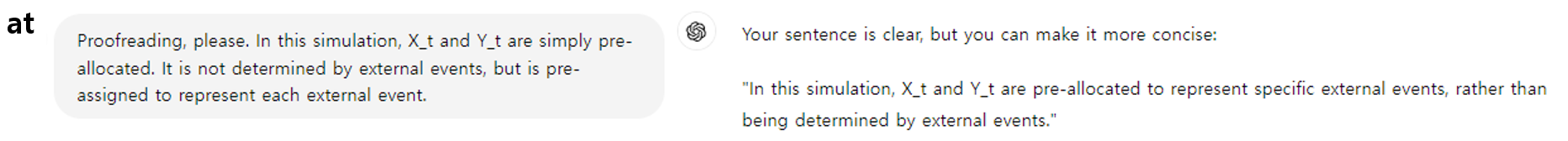


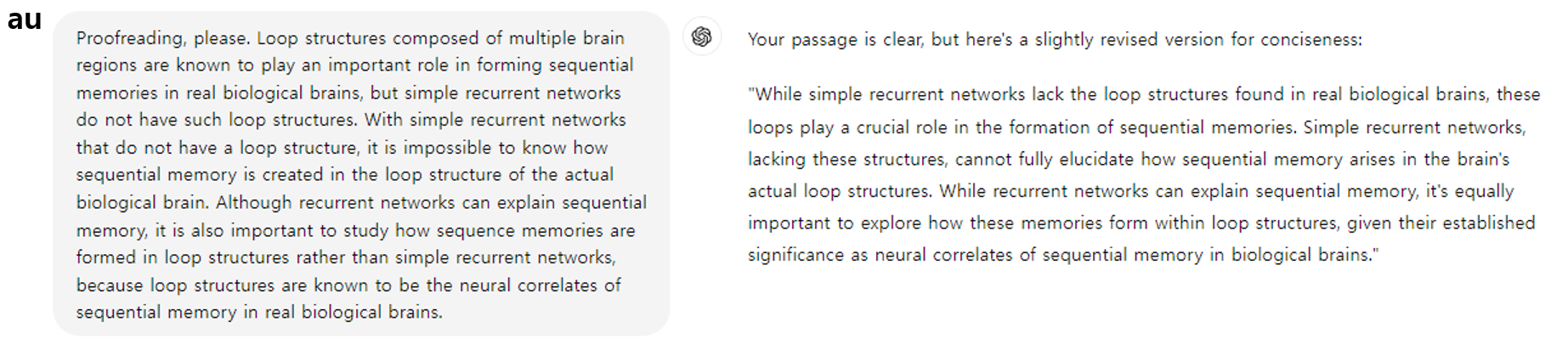


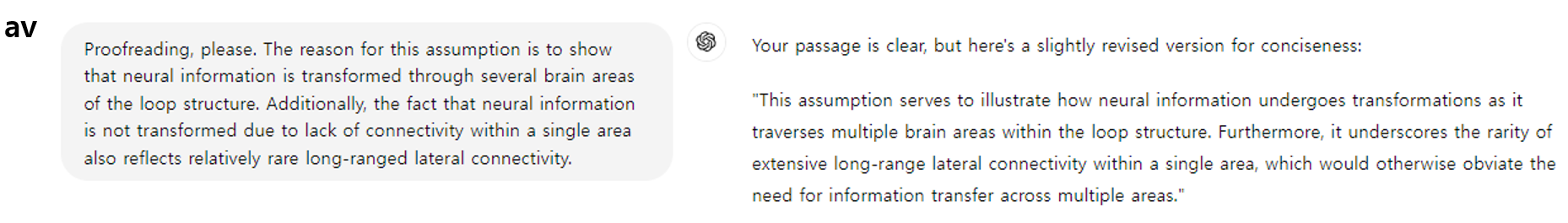


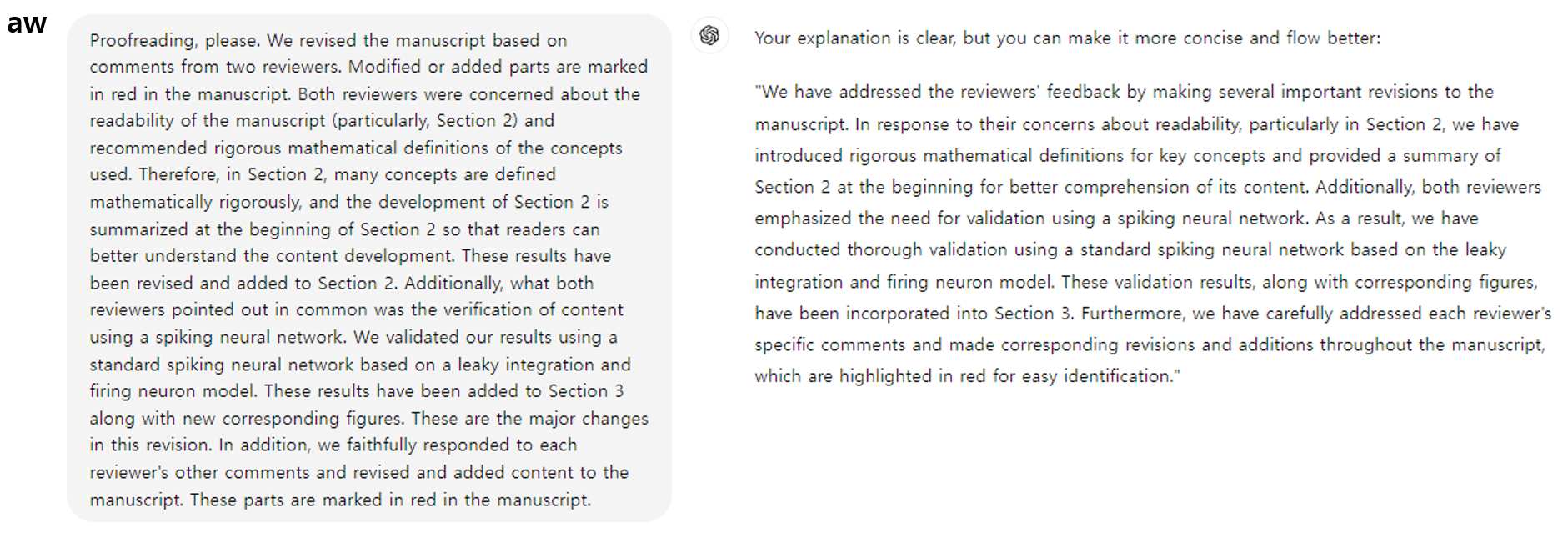


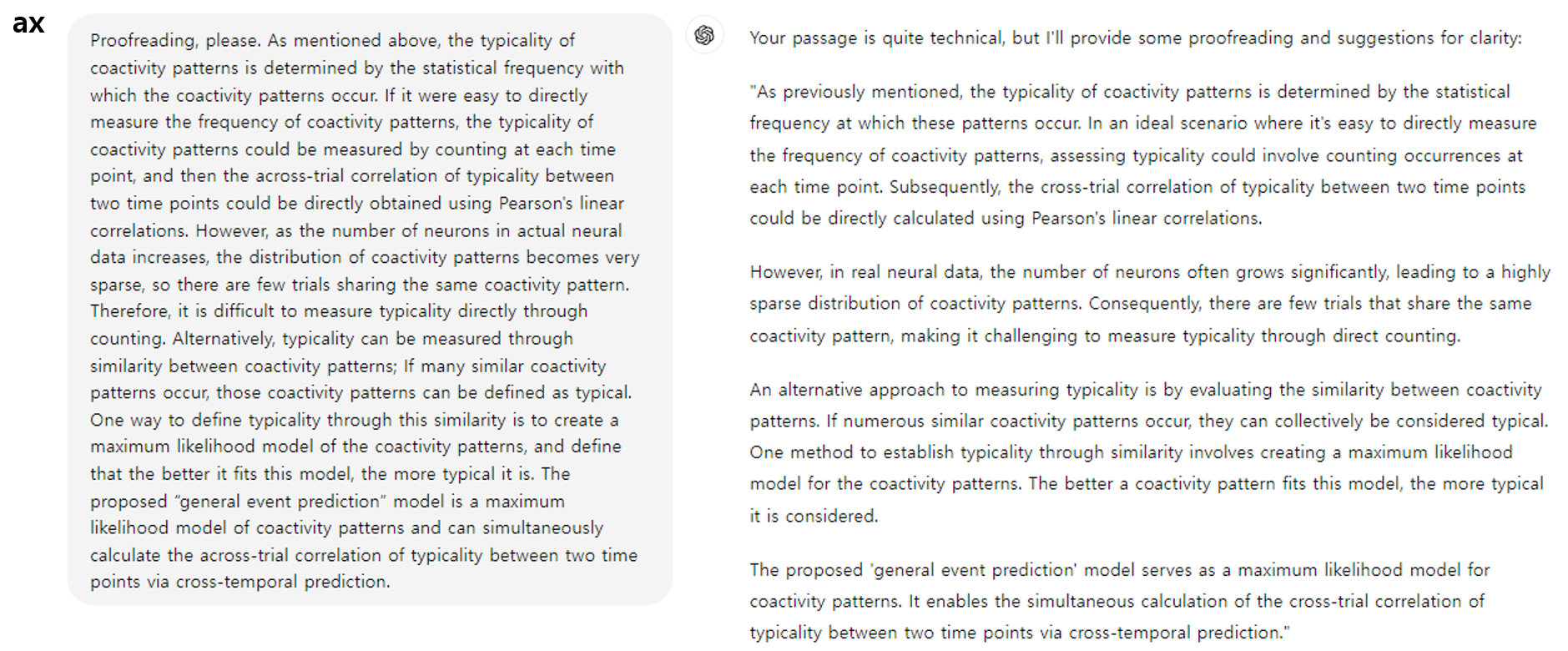


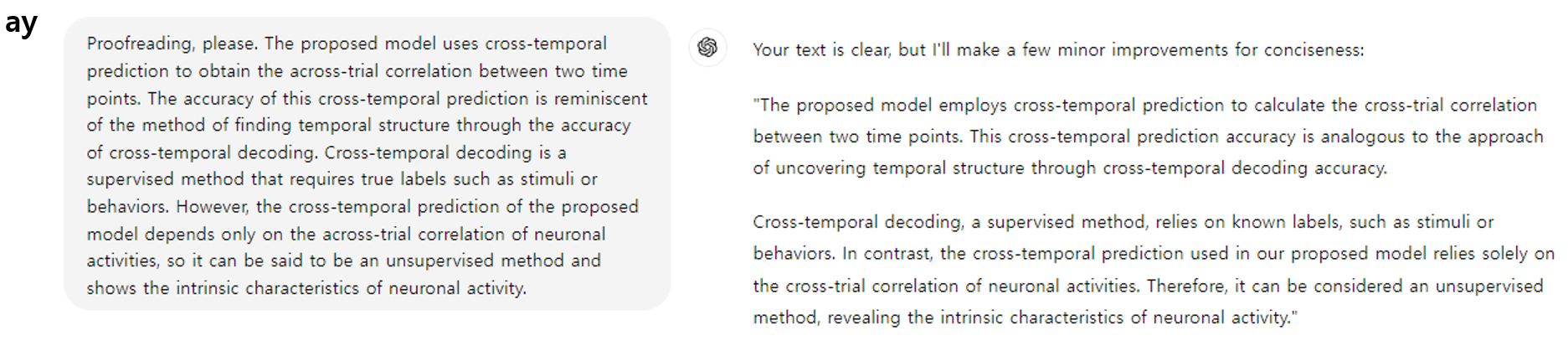


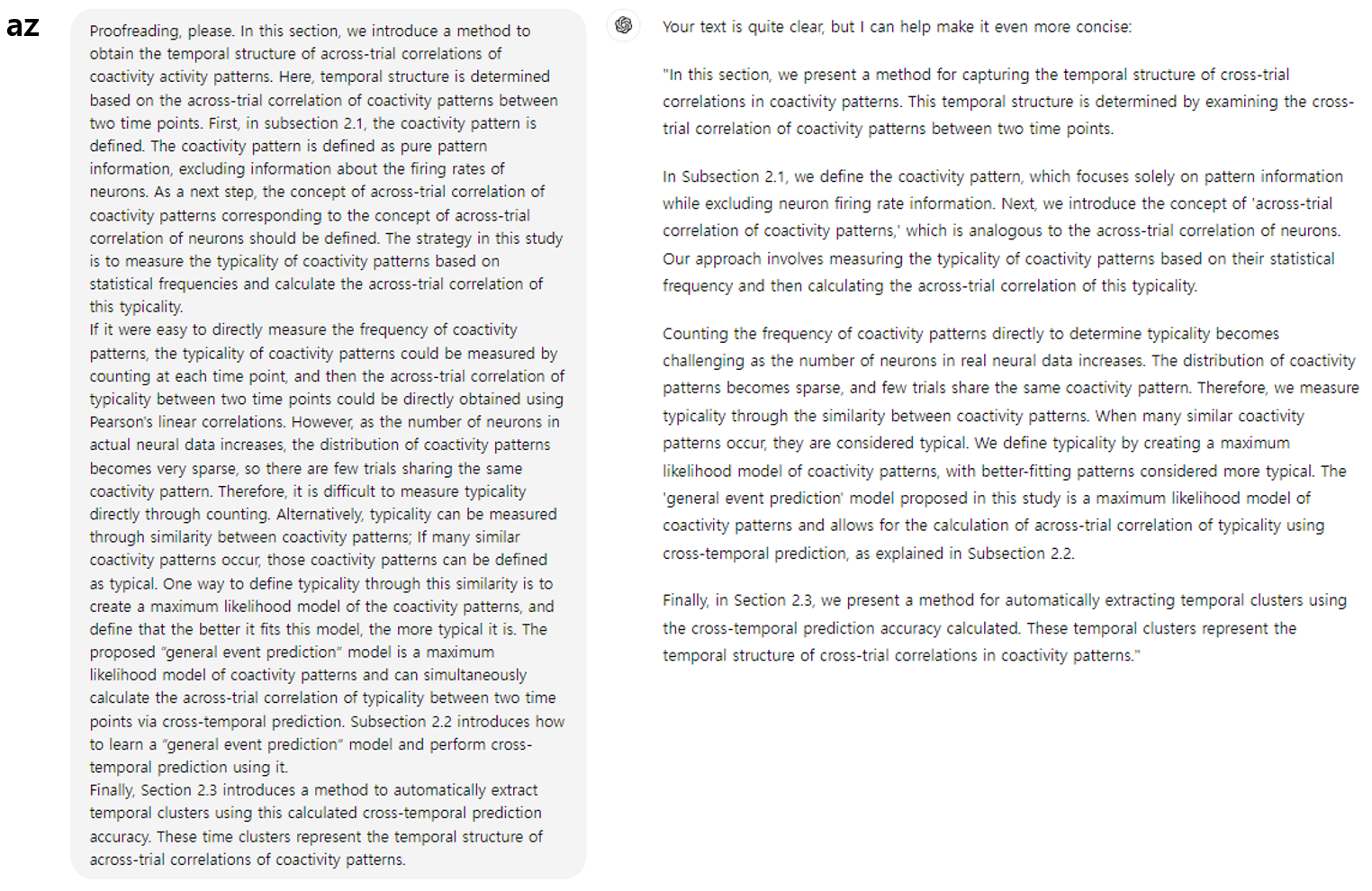


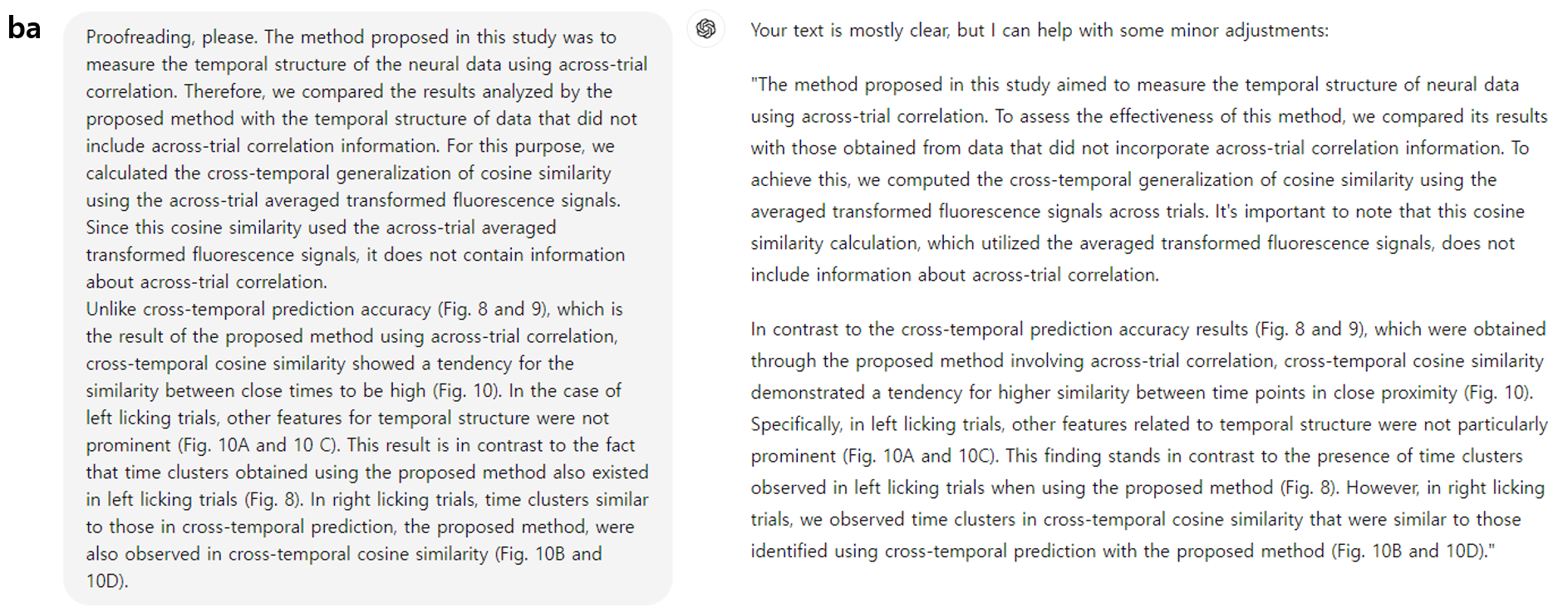


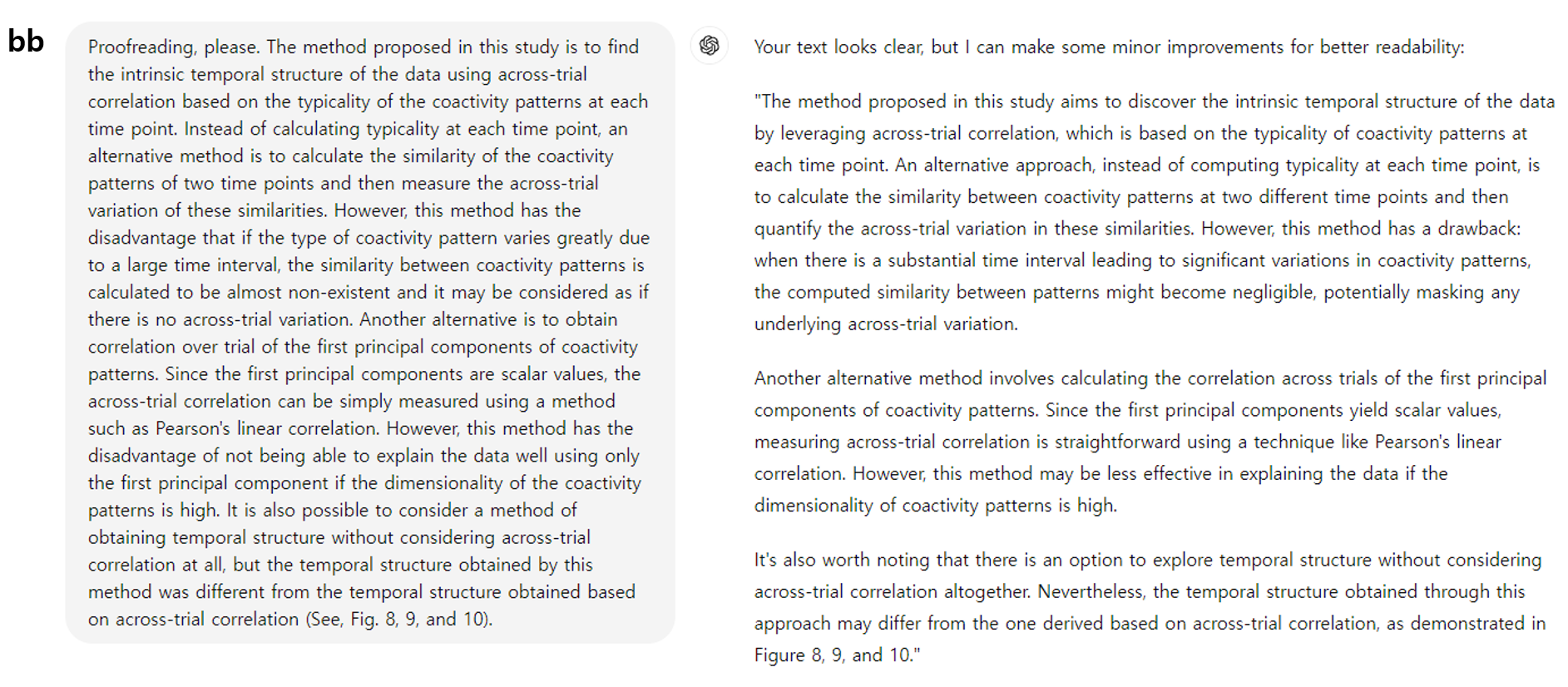


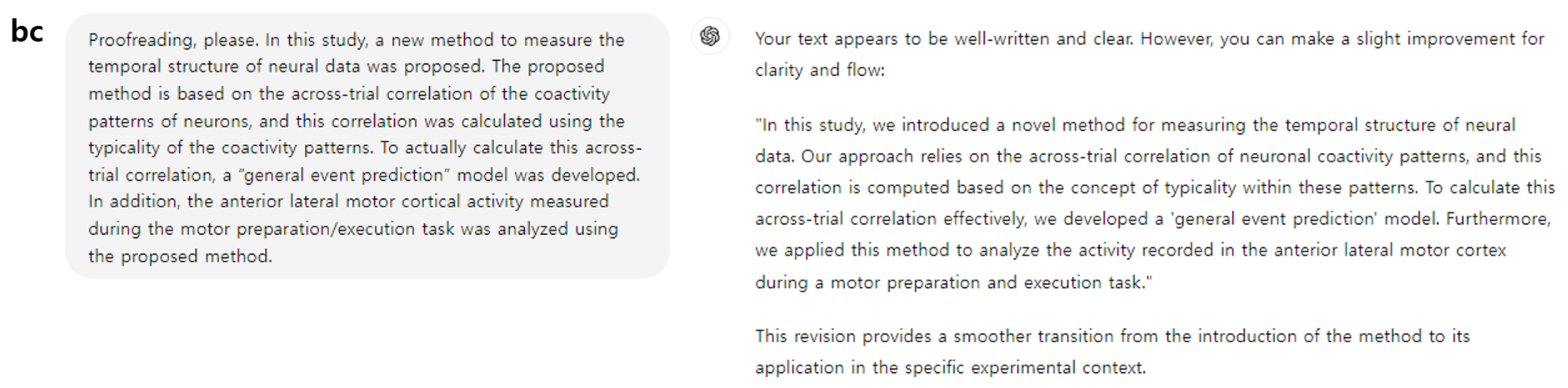


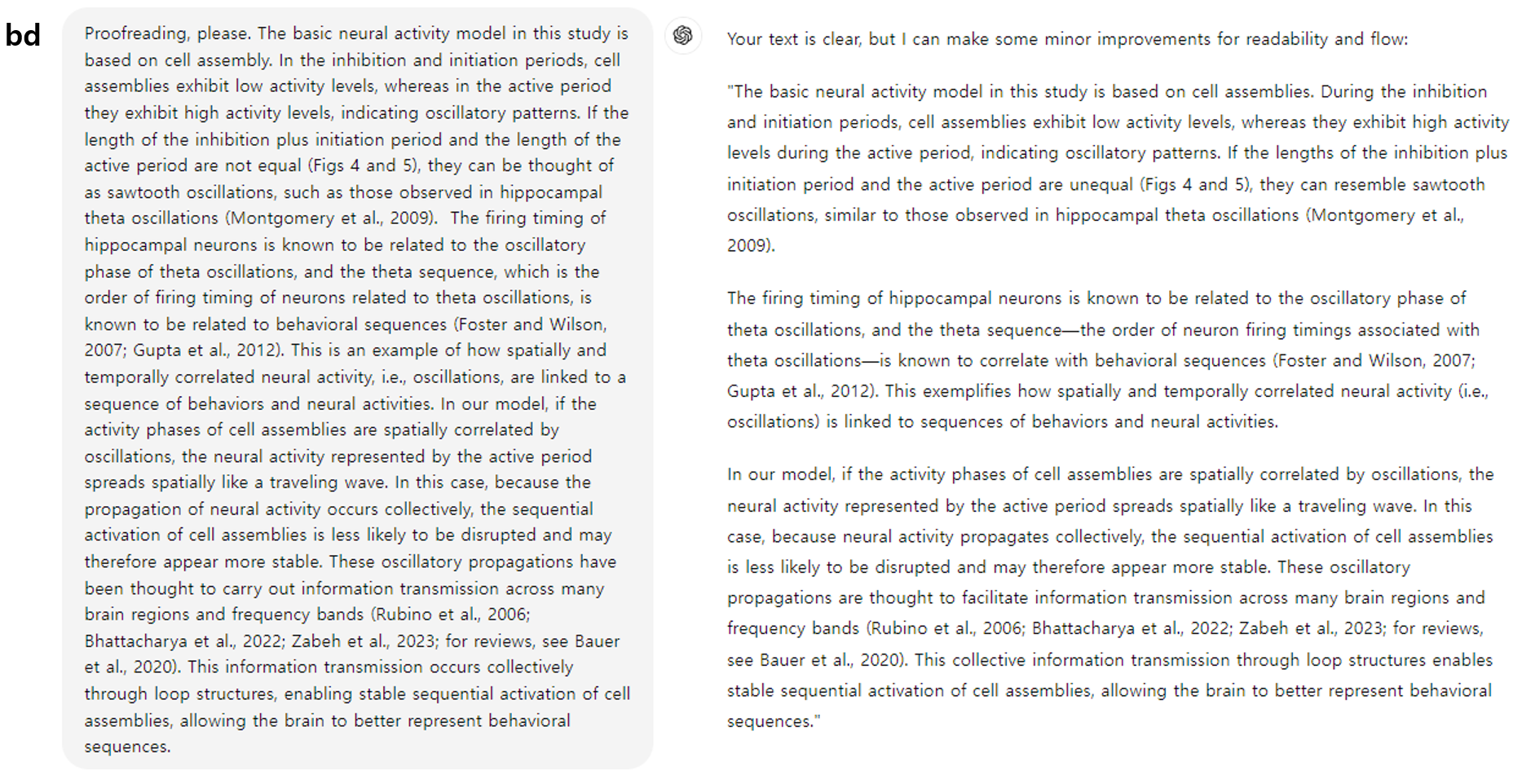


**Supplementary Figure 1.** All input prompts provided to a generative AI technology and all outputs received from a generative AI technology (ChatGPT 4.0).
